# A small RNA from *Streptococcus suis* epidemic ST7 strain promotes bacterial survival in host blood and brain by enhancing oxidative stress resistance

**DOI:** 10.1080/21505594.2025.2491635

**Published:** 2025-04-16

**Authors:** Zijing Liang, Shuoyue Wang, Xinchi Zhu, Jiale Ma, Huochun Yao, Zongfu Wu

**Affiliations:** a MOE Joint International Research Laboratory of Animal Health and Food Safety, College of Veterinary Medicine, Nanjing Agricultural University, Nanjing, China; b Key Lab of Animal Bacteriology, Ministry of Agriculture, Nanjing, China; c WOAH Reference Lab for Swine Streptococcosis, Nanjing, China; d Guangdong Provincial Key Laboratory of Research on the Technology of Pig-Breeding and Pig-Disease Prevention, Guangdong Haid Institute of Animal Husbandry & Veterinary, Guangzhou, China

**Keywords:** *Streptococcus suis*, small RNA, pathogenesis, aquaporin, oxidative stress resistance

## Abstract

*Streptococcus suis* is a Gram-positive pathogen causing septicaemia and meningitis in pigs and humans. However, how *S. suis* maintains a high bacterial load in the blood and brain is poorly understood. In this study, we found that a small RNA rss03 is predominantly present in *S. suis*, *Streptococcus parasuis*, and *Streptococcus ruminantium*, implying a conserved biological function. rss03 with a size of 303 nt mainly exists in *S. suis* sequence type (ST) 1 and epidemic ST7 strains that are responsible for human infections in China. Using MS2-affinity purification coupled with RNA sequencing (MAPS), proteomics analysis, and CopraRNA prediction, 14 direct targets of rss03 from an ST7 strain were identified. These direct targets mainly involve substance transport, transcriptional regulation, rRNA modification, and stress response. A more detailed analysis reveals that rss03 interacts with the coding region of *glpF* mRNA, and unexpectedly rss03 protects *glpF* mRNA from degradation by RNase J1. The GlpF protein is an aquaporin, contributes to *S. suis* oxidative stress resistance by H_2_O_2_ efflux, and facilitates bacterial survival in murine macrophages RAW264.7. Finally, we showed that rss03 and GlpF are required to maintain a high bacterial load in mouse blood and brain. Our study presents the first sRNA targetome in streptococci, enriches the knowledge of sRNA regulation in streptococci, and identifies pathways contributing to *S. suis* pathogenesis.

## Introduction

*Streptococcus suis* is a Gram-positive pathogen that can cause septicaemia, meningitis, and other diseases in pigs, leading to severe economic losses in the pig industry globally [1]. In addition, it is also an important zoonotic pathogen for humans with close contact with pigs or by-products [2]. Currently, 29 serotypes (SS1–19, 21, 23–25, 27–31, and 1/2) based on capsular polysaccharide (CPS) antigens, along with serotype Chz and several novel *cps* loci, have been identified [3,4]. Among these, *S. suis* serotype 2 (SS2) strains are the primary cause of infections in pigs and humans worldwide, posing a significant public health challenge [5]. In addition, strains can be genetically classified into sequence types (ST), which seem to have a geographical distribution [6]. Globally, ST1 strains are the most frequently isolated from human cases [7]. In China, two outbreaks of human infections caused by ST7 epidemic strains, which exhibited higher pathogenicity than ST1, occurred in Jiangsu Province in 1998 and Sichuan Province in 2005, both resulting in high mortality rates. Since 2005, ST1 and ST7 have been the predominant strains responsible for human infections in China [7]. A high bacterial load in the blood and brain is crucial for causing meningitis [8]. However, little is known about how *S. suis* survives in blood and maintains a high bacterial load in the brain.

Recent studies have highlighted the critical role of small RNAs (sRNA) as virulence regulators in pathogenic bacteria [9]. sRNAs play critical roles in adapting to complex stress conditions, such as nutrient availability [10], oxidative stress [11], and iron starvation [12]. By engaging in incomplete base-pairing interactions with target mRNAs, sRNAs can modulate target mRNA stability by blocking or recruiting RNases and regulate the exposure or occlusion of ribosomal binding sites (RBS) to influence target mRNA translation [13]. Compared with Gram-negative bacteria, research on sRNAs and their regulatory mechanisms in Gram-positive bacteria have lagged behind [14]. Although some sRNAs have been reported in *Streptococcus* species, such as *Streptococcus pneumoniae* [15,16], *Streptococcus pyogenes* [17,18], *Streptococcus mutans* [19,20], and *S. suis* [21,22], their specific regulatory mechanisms are still poorly understood. In our previous study, we first identified 29 sRNAs using a differential RNA-sequencing approach and RNAs from *S. suis* grown in rich medium, pig blood, or cerebrospinal fluid (CSF) [8]. Among these sRNAs, rss03 was up-regulated under CSF condition, and its expression was validated by northern blot analysis; furthermore, rss03 was found to enhance *S. suis* survival in pig blood, and the deletion of rss03 resulted in a reduction of *S. suis* virulence in zebrafish infection model [8]. However, its targets and specific roles in *S. suis* pathogenesis are unknown.

In this study, we employed a combination of experimental and computational approaches to identify the targets of rss03 from *S. suis* ST7 strain SC070731. We discovered that rss03 enhances the mRNA stability of its direct target mRNA *glpF*, which encodes an aquaporin, and found that GlpF facilitates H_2_O_2_ efflux. Furthermore, we observed that both rss03 and GlpF contribute to oxidative stress resistance of *S. suis*, bacterial survival in murine macrophages, and maintaining a high bacterial load in the blood and brain in mice. These findings not only enhance our understanding of the mechanism behind *S. suis* pathogenesis, but also provide the first sRNA targetome and valuable insights into the regulatory mechanisms of sRNAs in *Streptococcus* species.

## Materials and methods

### Bacterial strains, plasmids, and growth conditions

The strains and plasmids used in this study are listed in Table S1. *S. suis* strains were cultured in Todd-Hewitt broth (THB, Hopebio, HB0311–3) or defibrinated sheep blood-containing agar medium (6%, vol/vol, Solarbio, TX0030) at 37°C with 5% CO_2_. For *S. suis* mutant selection, the medium contained 100 μg/mL spectinomycin (Spc, Macklin, S6106) and 10% (wt/vol) sucrose (Macklin, S818049). *Escherichia coli* strains were cultured in Luria-Bertani (LB, Becton Dickinson, DF0446-17-3) broth or agar medium at 37°C. The medium for *E. coli* harbouring recombinant plasmids contained 50 μg/mL Spc, 50 μg/mL kanamycin (Aladdin, K331597), or 50 μg/mL ampicillin (Aladdin, A433388).

### Construction of deletion mutants and complemented strain

Deletion mutant *S. suis* strains were constructed using a two-step natural transformation method [23]. In the first step, *rss03* sequence was replaced with a *SacB-Spc* cassette, resulting in a strain that was sensitive to sucrose but resistant to spectinomycin. The second step replaced the cassette through negative selection on a sucrose THB agar plate. This method was applied to generate deletion mutants in strain SC070731 background for RNase III (*rnc*, *NJAUSS_RS05775*) and *glpF* (*NJAUSS_RS09410*), as well as in Δ*rss03* strain background for RNase III (Δ*rss03*Δ*rnc*). Primers are listed in Table S2. For complementation of *rss03* (CΔ*rss03*), a PCR fragment containing the *rss03* with its original putative promoter (−150 to +303 relative to the transcriptional start site (TSS)) was cloned into the pSET2 plasmid [24], and the recombinant plasmid was then transformed into Δ*rss03* strain.

### Sequence alignment, phylogenetic, and gene synteny analysis

A neighbour-joining phylogenetic tree was constructed in MEGA X 10.1.8 [25] using 178 nucleotide sequences of rss03 homologs from various *Streptococcus* species. A maximum-likelihood phylogenetic tree was constructed in MEGA X 10.1.8 using 21 amino acid sequences of GlpF homologs from various species. These sequences were aligned using MUSCLE [26]. The tree was visualized and annotated using Chiplot [27].

### 3’ RACE mapping

Rapid amplification of cDNA ends (RACE) was performed by referring to the SMARTer™ RACE cDNA Amplification Kit (Takara, 634858) instructions to determine the 3’ transcription termination site of rss03. A first PCR was performed using primers GSP2-rss03/UPM-Long, followed by a nested PCR with primers NGSP2-rss03/UPM-Short. Primers are listed in Table S2. The PCR products were cloned using the pMD19-T plasmid within *E. coli*, and samples were sequenced.

### Differential proteomics analysis

SC070731 strain (WT) and Δ*rss03* were grown at 37°C to exponential phase by inoculating 20 mL of THB medium with an overnight culture (1:100). Subsequently, strains were centrifuged. The pellets were resuspended in an SDT solution (4% SDS, 100 mM Tris-HCl pH 7.6, and 100 mM DTT) and then transferred to Lysis Matrix B Tubes (MP Biomedicals, 116911050). Lysis was performed with the FastPrep apparatus (MP Biomedicals 116005500). The supernatant was filtered, collected, and stored at −80°C. The samples were subsequently sent to Applied Shanghai Protein Technology Co., Ltd. (APTBIO, Shanghai, China) for the Liquid chromatography – tandem mass spectrometry (LC−MS/MS) analysis. Each strain was analysed with three biological replicates. The screening criteria for differentially expressed proteins were a fold change (FC) >1.5 and *p* < 0.05, or FC < 0.67 and *p* < 0.05 compared to the control group (WT).

### Candidate targets prediction by CopraRNA

The NCBI reference genome number and the conserved sequence of sRNA rss03 are listed in Table S3. The sRNA rss03 candidate targets were predicted in the *S. suis* strain SC070731 genome using CopraRNA [28]. The screening criterion of the candidate targets was based on *p*-value (*p* < 0.05) [28].

### RNA extraction and quantitative real-time PCR (RT-qPCR)

Detailed procedures for RNA extraction and RT-qPCR can be found in our previous study [29]. The bacterial RNA was extracted using the FastRNA Pro Blue Kit (MP Biomedicals, 116025050), following the manufacturer’s protocol. Genomic DNA was removed using DNase I (Takara, 2270A), and the RNA samples were purified using PCI (phenol:chloroform:isoamyl alcohol = 25:24:1) reagent (Solarbio, P1011) and then precipitated with three volumes of cold absolute ethanol in the presence of sodium acetate (0.3 M, Invitrogen, AM9740). The purified RNA samples were stored at −80°C. cDNA synthesis was performed using a reverse transcription kit (Vazyme, R323–01), following the manufacturer’s protocol. For RT-qPCR, ChamQ Universal SYBR qPCR Master Mix (Vazyme, Q711-02) was used, and the reactions were carried out on a QuantStudio 6 Flex instrument (Thermo Fisher, 4485697) according to the manufacturer’s instructions. The transcript levels of genes were normalized to the levels of *NJAUSS_RS04270* (*parC*), which remained constant under different stress conditions [8]. The primers for RT-qPCR are listed in Table S2. The relative fold change was calculated using the 2^−ΔΔCT^ method. Each group consisted of three biological replicates.

### RNA half-life determination assay

Strains grown to stationary phase at 37°C were treated with 0.3 mg/mL rifampicin (Aladdin, R105455). A 2 mL mixture was taken and centrifuged at 0, 2, 4, 8, and 16 min, then RNA was extracted, and RT-qPCR was used to determine RNA half-life [30,31]. The transcript levels of genes were normalized to 16S rRNA. Each group consisted of three biological replicates.

### MS2-affinity purification coupled with RNA sequencing (MAPS)

MAPS assays were performed as described previously [32]. The Δ*rss03* strain containing pSET2-MS2-*rss03* or WT strain containing pSET2-MS2-negative (used as a negative control) were grown in 1200 mL THB medium to exponential phase. Subsequently, strains were centrifuged, and the pellets were resuspended in 24 mL of pre-cooled Buffer A (20 mM Tris-HCl pH 8.0, Aladdin, T301502; 150 mM KCl, Aladdin, P112134; 1 mM MgCl_2_, Aladdin, M116336; and 1 mM DTT, Aladdin, D104860). The resuspended samples were then transferred to Lysis Matrix B Tubes (MP Biomedicals, 116911050) for lysis using the FastPrep apparatus (MP Biomedicals, 116005500). After centrifugation, the supernatants were collected and stored at 4°C until further use in the affinity chromatography step. The purification of MS2 coat protein fused to maltose binding protein (MS2-MBP) was performed as previously described [33]. During the affinity chromatography purification, all steps were carried out at 4°C. For each sample, 300 μL of amylose resin per column was used. The columns containing the amylose resin were washed three times with 10 mL Buffer A, and 0.616 μM MS2-MBP (dissolved in 6 mL of Buffer A) were then immobilized in each column. Next, each column received 8 mL of lysate and was washed with 10 mL of Buffer A three times. For elution, 2 mL of pre-cooled Buffer E (containing 20 mM Tris-HCl pH 8.0, Aladdin, T301502; 150 mM KCl, Aladdin, P112134; 1 mM MgCl_2_, Aladdin, M116336; 1 mM DTT, Aladdin, D104860; 0.1% Triton X-100, Macklin, T6328; and 12 mM maltose, Macklin, M874782) was used. The eluted RNAs were extracted and sent to Shanghai Realbio Technology Co., Ltd (Shanghai, China) for RNA sequencing. Each strain was analysed with two biological replicates. The screening criteria for differential expression were a |Log_2_FC| > 1 and *p* < 0.05 compared with the control group.

### *In vitro* transcription, RNA labelling, and gel retardation

DNA fragments with a T7 promoter were obtained through PCR amplification using primers listed in Table S2. These fragments were used for RNA production via *in vitro* transcription with T7 RNA polymerase (Thermo Fisher, K0441), which were then purified using DNase I (Takara, 2270A) and PCI reagent (Solarbio, P1011). The purified RNA samples were dissolved with 50 μL of nuclease-free water, and an equal volume of Urea blue (8 M urea, Macklin, U6209; 1% xylene cyanol, Solarbio, X8010; and 1% bromophenol blue, Solarbio, IB6630) was added. The mixture was denatured for 3 min at 95°C, chilled for 2 min on ice, and then electrophoresed in a 5% polyacrylamide denaturing gel at 4°C for 6 h (300 V). The target RNA was selected, placed in elution buffer (0.5 M ammonium acetate, Macklin, A801001; 1 mM EDTA, Innochem, B94680; and 0.2% SDS, Innochem, B38483) overnight, purified using PCI reagent (Solarbio, P1011), and stored at −80°C.

The 5’ ends of RNA were dephosphorylated using Antarctic phosphatase (NEB, M0289V). Subsequently, 5’ end-radiolabelled RNA was generated using T4 polynucleotide kinase (Thermo Fisher, EK0031) and [γ^32P^]-ATP (PerkinElmer, neg502A), and further filtered through Micro Bio-Spin 6 Tris Columns (BIO-RAD, 732-6221) and stored at −80°C.

The purified 5’ end-radiolabelled RNAs (27,000 cpm/sample) and non-radiolabelled RNAs were separately incubated in 5× Less MgCl_2_ Buffer (100 mM Tris-HCl pH 7.5, Aladdin, T301492; 300 mM KCl, Aladdin, P112134; 200 mM NH_4_Cl, Macklin, A801304; and 15 mM DTT, Aladdin, D104860) at 90°C for 1 min. After 1 min on ice, the sample with 10 mM MgCl_2_ was incubated at 25°C for 15 min. For each assay, the 5’ end-radiolabelled RNAs and varying amounts of the different RNAs (typically 0, 125, 250, 500, 1000 nM) were added to a final volume of 10 μL containing 5× Plus MgCl_2_ Buffer (100 mM Tris-HCl pH 7.5, Aladdin, T301492; 300 mM KCl, Aladdin, P112134; 200 mM NH_4_Cl, Macklin, A801304; 15 mM DTT, Aladdin, D104860; and 50 mM MgCl_2_, Aladdin, M116336), followed by the addition of an equal volume of Glycerol blue (40% glycerol, Hushi, 10010618; 1% xylene cyanol, Solarbio, X8010; and 1% bromophenol blue, Solarbio, IB6630). The mixture was incubated at 37°C for 15 min and then electrophoresed in a 5% polyacrylamide non-denaturing gel containing 10 mM MgCl_2_ (Aladdin, M116336) at 4°C for 4 h (300 V). Digital images of the radioactive complex samples were obtained using a storage phosphor screen (Cytiva, BAS-IP MS2025E) and a molecular imager instrument (GE, Typhoon Trio). The equilibrium dissociation constant (Kd), representing the radiolabelled RNA concentration showing 50% binding, was measured following the previously described method [34].

### RNases *in vitro* degradation assay

To express the recombinant RNase III, RNase J1, and RNase J2 proteins, the coding regions were amplified from genomic DNA and inserted into the pET28a plasmid. The recombinant proteins were expressed in BL21 cells from the pET28a-*rnc*, pET28a-*rnj1*, pET28a-*rnj2* plasmid and purified using nickel-nitrilotriacetic acid spin columns (Cytiva, 17524701). Primers are listed in Table S2. The protein concentration was determined using the BCA protein quantification kit (Vazyme, E112-01), and glycerol (10%, vol/vol, Hushi, 10010618) was added for storage at −80°C. The purified 5’ end-radiolabelled *glpF*-400 nt mRNA (*glpF*-400 nt*, 27,000 cpm/sample) and non-radiolabelled sRNA rss03 were separately incubated in 5× Less MgCl_2_ Buffer at 90°C for 1 min. After 1 min on ice, the sample was incubated at 25°C for 15 min with 10 mM MgCl_2_. For each assay, tubes containing *glpF*-400 nt*, 1×RNase Buffer (20 mM Tris-HCl pH 7.5, Aladdin, T301492; 8 mM MgCl_2_, Aladdin, M116336; 0.1 M NH_4_Cl, Macklin, A801304; and 0.1 mM DTT, Aladdin, D104860), and either non-radiolabelled sRNA rss03 (500 nM) or none. The mixtures were then incubated at 37°C for 15 min. Next, each tube, with a final volume of 10 μL, was supplemented with RNase III, RNase J1, or RNase J2 at a concentration of 800 nM. Subsequently, the samples were incubated at 37°C for 0, 15, 30, and 60 min. The samples were mixed with an equal amount of Urea blue and electrophoresed in 5% polyacrylamide denaturing gel at 4°C for 3 h (300 V). The digital images of the radioactive complex samples were captured using a storage phosphor screen (Cytiva, BAS-IP MS2025E) and a molecular imager instrument (GE, Typhoon Trio).

### β-galactosidase activity assay

Transcriptional fusions were constructed using pTCV, a low-copy-number plasmid lacking a promoter and encoding *lacZ* [35]. Primers are listed in Table S2. The strains containing the recombinant pTCV plasmid were grown at 37°C until reaching the stationary phase. Four millilitres of cultures were concentrated to 400 μL, while 400 μL of THB medium was a negative control. Sample preparation involved the addition of 8 μL of lysozyme (2.5 mg/mL, Biosharp, BS184), 25 μL of 4×Z-Buffer (240 mM Na_2_HPO_4_·2 H_2_O, Macklin, S821245; 160 mM NaH_2_PO_4_·2 H_2_O, Macklin, S817463; 40 mM KCl, Aladdin, P112134; and 4 mM MgSO_4_·7 H_2_O, Aladdin, M110771, pH 7.0), and 0.35 μL of β-mercaptoethanol (Aladdin, M301574), followed by incubation at 30°C for 30 min. Subsequently, 400 μL of 1×Z-Buffer (containing 0.8 mg of O-nitrophenyl-β-D-galactopyranoside, Solarbio, O8040) and 1.1 μL of β-mercaptoethanol (Aladdin, M301574) were added, and the mixture was incubated at 37°C until the colour changed to yellow. After the addition of 400 μL of 1 M Na_2_CO_3_ (Hushi, 10019260) and centrifugation, 200 μL of the supernatant was used to measure OD_420_. The activity of β-galactosidase was calculated using the following equation [36]: Activity (Miller Units) = (OD_420_ × 1000 × V_E_)/(Vs × RT × OD_600_), where RT represents the reaction time (min), V_E_ represents the end volume, and Vs represents the volume of each sample (400 µL). The relative β-galactosidase activity of a certain *S. suis* strain was calculated as the β-galactosidase activity of that strain divided by the β-galactosidase activity of the WT strain containing *glpF-lacZ*. Each experimental group comprised three biological replicates.

### Oxidative stress and intracellular H_2_O_2_ concentration measurement assays

To assess the role of the rss03 or GlpF in oxidative stress response, WT, Δ*rss03*, CΔ*rss03*, and Δ*glpF* were cultured in 5 mL of THB medium and grown at 37°C to the exponential phase. Subsequently, a final concentration of 40 mM H_2_O_2_ (Hushi, 10011218) was added, and the mixtures were incubated at 37°C with 5% CO_2_ for 25 min. After appropriate dilution, samples from the mixtures were spread onto THB agar plates and incubated at 37°C overnight to determine bacterial numbers. The survival rate was calculated by dividing the bacterial number after H_2_O_2_ treatment by the number before treatment. Each strain was tested in three biological replicates.

WT and Δ*glpF* mutant were cultured in 5 mL of THB medium and grown at 37°C to the exponential phase. Subsequently, a final concentration of 40 mM H_2_O_2_ was added, and the mixtures were incubated at 37°C with 5% CO_2_ for 25 min. After centrifugation and washing once with PBS, the pellets were resuspended in 1 mL acetone. The resuspended samples were then transferred to Lysis Matrix B Tubes (MP Biomedicals, 116911050) for lysis using the FastPrep apparatus (MP Biomedicals, 116005500). After centrifugation, the supernatants were collected. Finally, the concentration of H_2_O_2_ in the supernatant was measured using a hydrogen peroxide assay kit (Solarbio, BC3595) following the instructions provided. Each strain was tested in three biological replicates.

### Intracellular survival assays

Murine macrophages RAW264.7 were purchased from the Cell Bank of the Chinese Academy of Sciences (Shanghai, China) and cultured in DMEM medium supplemented with glucose (Gibco, 11965092) and 10% foetal bovine serum (Gibco, 10438026) and incubated at 37°C with 5% CO_2_. *S. suis* strains were then grown to the exponential phase at 37°C and washed thrice with PBS (Boster, PYG0021). The RAW264.7 cells at a density of 5 × 10^5^ cells per well were infected with *S. suis* strains at a 100:1 MOI (bacteria: macrophages). After 1 h of infection, the cells were washed thrice with DMEM medium containing glucose (Gibco, 11965092). The medium was subsequently supplemented with 100 mg/mL of gentamicin (Macklin, G6064) and 5 mg/mL of penicillin (Aladdin, P433334), and the cells were further incubated at 37°C for 3 h. At 1 h and 3 h, DMEM medium containing glucose was used again to wash the cells, and 1 mL of nuclease-free water was added for lysis. The resulting cell lysate was further subjected to serial dilutions and inoculated onto THB agar plates. The survival rate of *S. suis* strains was calculated as CFU at the 3-h time point divided by CFU at the 1-h time point. The relative survival rate of a certain *S. suis* strain was calculated as the survival rate of that strain divided by the survival rate of the WT strain. Each strain was tested in three biological replicates.

### Animal infection model

SPF BALB/c female mice (6 weeks old) were purchased from Shanghai SLAC Laboratory Animal Co. Ltd. (Shanghai, China). All mice were housed in standard plastic mouse cages and kept under constant room temperature (23 ± 3°C), humidity (55 ± 5%). The BALB/c mouse infection model was employed to evaluate the impact of rss03 or GlpF on the virulence of *S. suis* strain SC070731. *S. suis* strains were incubated until reaching an OD_600_ of 0.6, washed thrice in PBS, and adjusted to the appropriate doses. Five mice per group were intraperitoneally injected with 3 × 10^8^ CFU/mouse for each strain, as reported in our previous study [36]. After 12 h of infection, blood, brain, spleen, liver, and kidneys were collected. Blood samples were drawn from the heart, while the other tissues were homogenized in specific amounts of PBS. All samples were then suitably diluted and spread on THB agar plates cultured at 37°C with CO_2_ overnight. Colony counting was performed to determine bacterial load. For the intracranial subarachnoid infection assay, 18 mice per group were intracranially injected with 3 × 10^7^ CFU/mouse for each strain following a previously established protocol [37]. After 12 h of infection, only the brain was collected for further analysis. The flowchart of animal infection assays was shown in Figure S1.

### Statistical analysis

Data represent the mean ± standard error of the mean (SEM), and a two-tailed unpaired *t* test was used for the experiments. GraphPad Prism software was used to perform all the statistical analysis.

## Results

### rss03 is conserved in *S. suis* and other *Streptococcus* species

**Figure 1.**
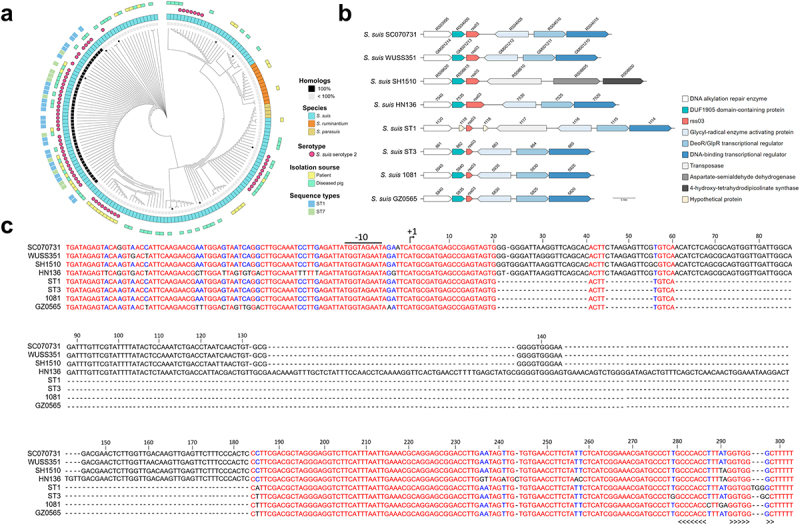
Distribution and conservative analysis of rss03. (a) rss03 homologs are restricted in *Streptococcus* genus. Homologs with 100% identity and coverage to rss03 of ST7 strain SC070731 are classified as 100% homologs, while those with less than 100% sequence identity or coverage were classified as <100% homologs. Clusters of diverse species and strains isolated from diseased pigs or humans are indicated with coloured boxes. *S. suis* strains with serotype 2, ST1, and ST7 are highlighted by different colours. Black dots represent *S. suis* strains selected for the analysis of genomic location, sequence, and predicted secondary structure. (b) Map of the location of rss03 homologs in genomes of various *S. suis* strains. Homologous genes are depicted using the same colour. Red arrows represent rss03 and its homologs. (c) Alignment of rss03 homologous sequences, including the promoter regions from various *S. suis* strains. Numbers indicate nucleotide positions. The −10 box, transcriptional start site (TSS, arrow), and Rho-independent terminator (brackets) are indicated. For these sRNAs, the regions conserved within *S. suis* have been shown by different colours: red indicating nucleotide identity 100%, blue indicating nucleotide identity ≥50%, and black indicating nucleotide identity <50%.

rss03 is located in intergenic regions, and its TSS was identified in our previous work [8]. The 3’ end of rss03 was determined by 3’ RACE assay (Figure S2a). Thus, the full length of rss03 is 303 nt in *S. suis* ST7 strain SC070731. Phylogenetic analysis revealed that rss03 homologs are predominantly present in *S. suis*, *Streptococcus parasuis*, and *Streptococcus ruminantium* (Figure 1a and Table S4). Notably, rss03 in *S. suis* ST1 and ST7 strains, sharing 100% identity, belong to the same branch with a size of 303 nt. Additionally, it was found that its homologs having identical sequences with that of ST7 strain SC070731 are primarily present in SS2 strains (39/45) and strains isolated from diseased pigs or humans (28/45) (Figure 1a), based on 178 strains whose complete genomes are available from NCBI database. The genomic location of rss03 homologs from the different branches in the evolutionary tree remains conserved in *S. suis* (Figure 1b). Furthermore, comparative sequence analysis found that the promoter region, 5’ end (1––20 nt, sharing 100% identity), 3’ end (184––303 nt, sharing 93.39––100% identity), and the putative Rho-independent terminator region of rss03 homologs exhibit a high degree of conservation among various *S. suis* strains (Figure 1c). The conserved regions of *S. suis* rss03 display a similar secondary structure predicted by RNAfold (Figure S2b). The length of *S. suis* rss03 ranges from 149 nt to 423 nt. Similarly, in *S. parasuis* and *S. ruminantium*, the genomic location, promoter, terminator region, and the predicted secondary structure of rss03 also show relative conservation (Figure S3). These findings suggest that rss03 may possess a conserved function and regulatory mechanism across *S. suis* and its closely related species.

### Characterization of rss03 targetome

**Figure 2.**
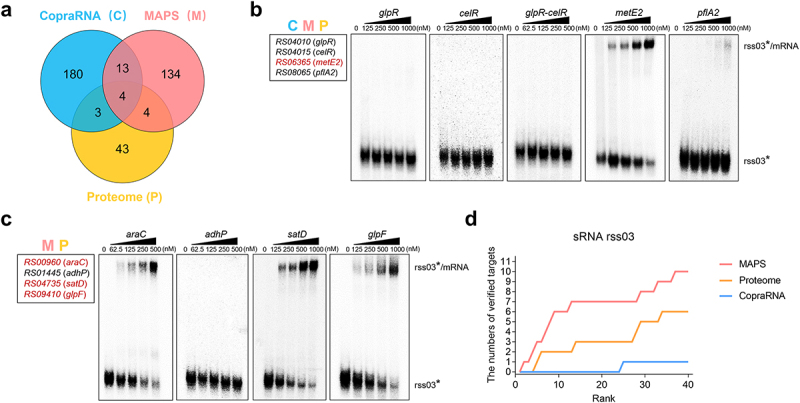
Integration of MAPS, CopraRNA, and proteomics analysis to identify rss03 targets. (a) The Venn diagram showing the overlap of the top 200 CopraRNA predictions, 155 candidates by MAPS, and 54 different expressed proteins by proteomics analysis. (b) Gel retardation assays using rss03 and candidate targets common to all three methods (C&M&P), C for CopraRNA, M for MAPS, and P for proteome. rss03* was incubated with increasing concentrations of *glpR*, *celR*, *metE2*, *pflA2* fragment, or *glpR-celR* operon, respectively. (c) Gel retardation assays using rss03 and candidate targets are common to M&P. rss03* was incubated with increasing concentrations of *araC*, *adhP*, *satD*, or *glpF* fragment, respectively. The genes marked red in the box indicate the direct targets based on gel retardation assays. For each assay, the first well is assigned as the negative control, where the corresponding candidate target of rss03 is not added. For gel source data, see Figure S4. (d) The cumulated number of well-characterized targets is plotted against the prediction rank for CopraRNA, MAPS, and proteome.

To predict potential targets of the rss03, we initially employed CopraRNA, a computational approach that integrates genomic-scale phylogenetic information [28]. The top 200 candidate targets obtained by CopraRNA are listed in Table S5. In addition, we utilized the MAPS, known for its accuracy in filtering sRNA targets in Gram-negative and Gram-positive bacteria [38,39]. Using this strategy, we identified 155 RNAs significantly enriched with MS2–rss03 (Table S6). Additionally, we employed a quantitative differential proteomics approach to compare the protein expression levels between WT and rss03 deletion mutant (Δ*rss03*), revealing significant differences in 54 proteins (Table S7). Among the candidate targets, four were identified by all three approaches, and an additional four were identified by both MAPS and proteomics (Figure 2a). Through gel retardation assays, we further demonstrated the formation of a stable complex between the 5’ end-radiolabelled of rss03 (rss03*) and the mRNA of *metE2*, *araC*, *satD*, and *glpF* (Figure 2b,c, and S4). To determine the most advantageous approach for screening rss03 direct targets, we randomly selected 15 candidate targets from the top 40 identified by each approach mentioned above. As the number of candidate targets increased, MAPS identified the highest direct targets (10 targets), while CopraRNA yielded the lowest (one target) (Figure 2d, S5, and S6). In total, we identified 14 mRNAs which formed stable complexes with sRNA rss03. They encode proteins involved in transcriptional regulation, genetic recombination, rRNA modification, stress response, and substance transport (Table 1). These findings indicate that *S. suis* sRNA rss03 has multiple direct targets, with MAPS proving to be the most effective method for identifying the direct targets of *S. suis* sRNAs.

**Table 1.** List of direct targets of sRNA rss03.

Number	ID	Gene	Function
1	NJAUSS_RS00960	*araC*	AraC family transcriptional regulator
2	NJAUSS_RS01765	*usp*	Universal stress protein
3	NJAUSS_RS03740	*rsuA*	rRNA pseudouridine synthase
4	NJAUSS_RS03755	*fepD*	Iron ABC transporter permease
5	NJAUSS_RS04735	*satD*	DNA-binding protein
6	NJAUSS_RS05510	-	Hypothetical protein
7	NJAUSS_RS06365	*metE2*	5-methyltetrahydropteroyltriglutamate-homocysteine methyltransferase
8	NJAUSS_RS06815	*nanR*	RpiR family transcriptional regulator
9	NJAUSS_RS08230	*xerD*	Site-specific tyrosine recombinase
10	NJAUSS_RS08625	*xerC*	Site-specific tyrosine recombinase
11	NJAUSS_RS09150	*araE*	Aromatic acid exporter family protein
12	NJAUSS_RS09285	*murR*	MurR family transcriptional regulator
13	NJAUSS_RS09410	*glpF*	Aquaporin family protein
14	NJAUSS_RS09450	*ssnA*	Deoxyribonuclease

### rss03 interacts with the coding region of *glpF*

**Figure 3.**
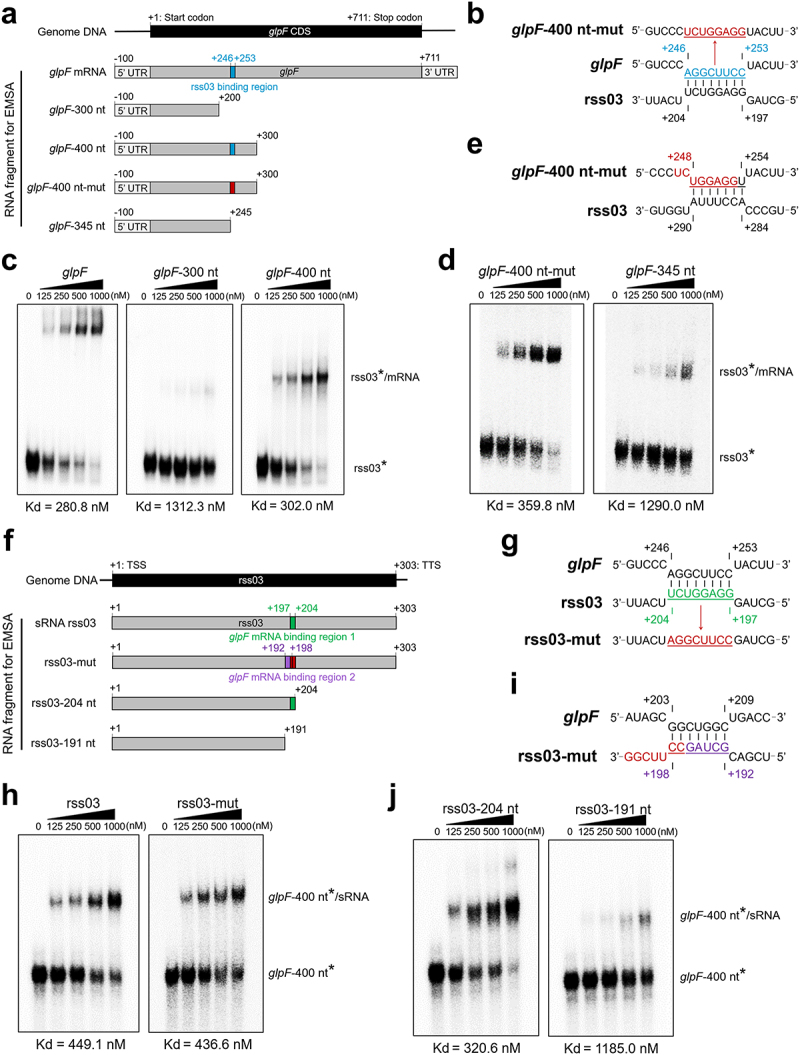
The binding regions between rss03 and *glpF* mRNA. (a) Schematic of the *glpF* transcript (*glpF* mRNA), the different truncated *glpF* transcript, and the mutant truncated *glpF* transcript (*glpF*-400 nt-mut). Numbers denote nucleotide positions relative to the start codon of *glpF*. (b) Schematic of predicted binding regions and mutant sites in *glpF* mRNA. Numbers denote nucleotide positions relative to the start codon of *glpF* or the TSS of rss03. (c) Gel retardation assays using rss03* and *glpF* mRNA (positive control), *glpF*-300 nt, or *glpF*-400 nt, respectively. (d) Gel retardation assays using rss03* and *glpF*-400 nt-mut, or *glpF*-345 nt, respectively. For each assay, the first well is assigned as the negative control, where the corresponding *glpF* mRNA variant is not added. (e) Schematic of predicted binding regions between rss03 and *glpF*-400 nt-mut. (f) Schematic of rss03 transcript (sRNA rss03), the mutant truncated rss03 transcript (rss03-mut), and the different truncated rss03 transcript. (g) Schematic of predicted binding regions and mutant sites in rss03. (h) Gel retardation assays using *glpF*-400 nt* and sRNA rss03 (positive control), or rss03-mut, respectively. (i) Schematic of the predicted binding region between *glpF* and rss03-mut. (j) Gel retardation assays using *glpF*-400 nt* and rss03–204 nt or rss03–191 nt, respectively. For each assay, the first well is assigned as the negative control, where the corresponding sRNA rss03 variant is not added. For gel source data, see Figure S8.

Recently, we discovered a novel aquaporin, Aagp, which promotes *S. suis* resistance to oxidative stress through hydrogen peroxide (H_2_O_2_) efflux and contributes to *S. suis* survival in the host [40]. GlpF, the direct target of rss03, is also a member of the aquaporin family protein and shares 34.07% amino acid identity with Aagp (Figure S7 and Table S8), so we selected GlpF for further investigation. Base pairing complementarities were predicted between nucleotides +246 to +253 of *glpF* mRNA and nucleotides +197 to +204 of rss03 (Figure 3a,b) using IntaRNA [41]. To test this prediction, we performed gel retardation assays using the rss03* and various truncated *glpF* transcripts. The results revealed the formation of a stable complex between rss03 and *glpF* (−100 ~ +711, Kd = 280.8 nM), or *glpF*-400 nt (−100 ~ +300, Kd = 302.0 nM), while the stability of the complex considerably dropped with *glpF*-300 nt (−100 ~ +200, Kd = 1312.3 nM) (Figure 3c and S8). These data support that the interaction region lies between +200 and +300 in the *glpF* transcript. To further validate the interaction sites, a mutated *glpF*-400 nt transcript (*glpF*-400 nt-mut, Figure 3a,b) was utilized. Unexpectedly, a stable complex was still able to form (Kd = 359.8 nM, Figure 3d and S8). IntaRNA predicted base pairing complementarity between the mutant region of *glpF*-400 nt-mut and another region of rss03 (Figure 3e). This result aligns with previous findings that altering the nucleotide sequence at the sRNA-RNA binding site might result in interactions with sequences exhibiting complementarity to the mutated binding site [42,43]. Therefore, a truncated *glpF*-345 nt transcript (−100 ~ +245) was constructed. As expected, only a very weak complex formation was observed (Kd = 1290.0 nM, Figure 3d). These results confirm that the coding sequence (+246 ~ +253) of the *glpR* transcript is essential for stable complex formation with rss03. To determine regions of rss03 binding to *glpF* mRNA, gel retardation assays were conducted using the *glpF*-400 nt* and the mutated rss03 (rss03-mut, Figure 3f,g). The results showed that a stable complex with rss03-mut was formed (Kd = 436.6 nM), similar to the complex formed with rss03 (Kd = 449.1 nM, Figure 3h and S8). Again, IntaRNA predicted that the mutant region of rss03-mut still contained base pairing complementarity with another region of *glpF* (Figure 3i). Therefore, truncated rss03–204 nt sRNA (+1 ~ +204) and rss03–191 nt sRNA (+1 ~ +191) were constructed (Figure 3f). While stable complexes were formed between rss03–204 nt and *glpF*-400 nt (Kd = 320.6 nM), only a very weak complex between *glpF*-400 nt and rss03–191 nt was observed (Kd = 1185.0 nM, Figure 3j and S8). Collectively, these findings support that base pairing interactions occurred between nucleotides +246 to +253 of *glpF* mRNA and nucleotides +197 to +204 of rss03.

### rss03 stabilizes the *glpF* transcript

**Figure 4.**
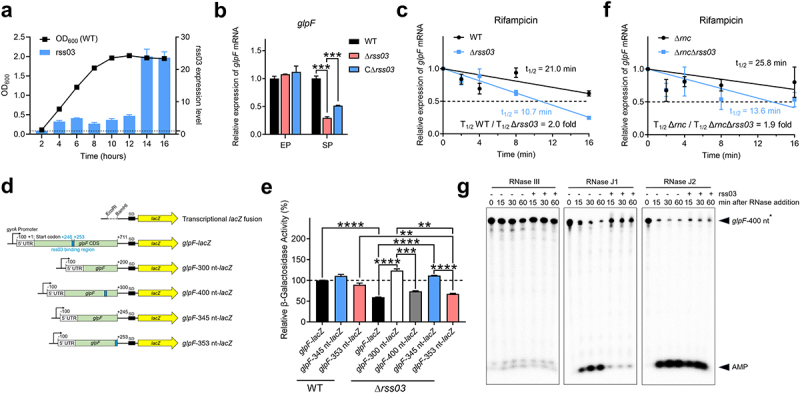
rss03 promotes the stability of *glpF* mRNA. (a) rss03 expression profile during a 16 h growth of WT strain. The growth curve of WT strain is presented by line, with the relative expression level of rss03 compared to housekeeping gene *parC*. (b) Relative expression level of *glpF* expression in WT, Δ*rss03*, and CΔ*rss03* during exponential phase (EP) or stationary phase (SP). (c) Decay curves of *glpF* mRNA in WT and Δ*rss03*. The signal obtained at 0 min was set to 1 for each strain, and the amount of RNA remaining at each timepoint was plotted on the y-axis versus time on the x-axis. The time at which 50% of the *glpF* mRNA decayed (dashed line) was calculated to determine the half-life (t_1/2_). Data represent the mean ± SEM, *n* = 3. (d) Schematic of all transcriptional *lacZ* fusions plasmid used in this study. SD, Shine-Dalgarno sequence. (e) β-galactosidase assays with the transcriptional *lacZ* fusion of the full-length (*glpF-lacZ*) or the different truncated *glpF* transcript in WT or Δ*rss03*. (f) Decay curves of *glpF* mRNA in Δ*rnc* and Δ*rnc*Δ*rss03*. (g) *In vitro* degradation of *glpF* mRNA by RNase III, J1, or J2 with or without the addition of sRNA rss03. For each assay, the first well is assigned as the negative control, where neither the corresponding RNase nor the sRNA rss03 is added. AMP is the first radiolabelled nucleotide. For gel source data, see Figure S9. Data represent the mean ± SEM, and asterisks indicate significantly different values (*n* = 3, ** indicates *p* < 0.01, *** indicates *p* < 0.001, **** indicates *p* < 0.0001, two-tailed unpaired *t* test).

In rich medium condition, compared with lag and exponential phases, the expression of *rss03* was highly increased in the stationary phase (Figure 4a). To investigate the impact of rss03 on *glpF* mRNA expression, we assessed the expression of *glpF* mRNA in WT, Δ*rss03*, and *rss03* complementary strain (CΔ*rss03*) at both the exponential and stationary phases. We found no significant difference in *glpF* mRNA expression in the three strains at the exponential phase. However, at the stationary phase, the expression of *glpF* mRNA in Δ*rss03* was significantly lower than that in WT and CΔ*rss03* (Figure 4b). To test whether rss03 affects *glpF* mRNA stability, we measured the half-life of *glpF* mRNA in both WT and Δ*rss03*. The results showed that *glpF* mRNA stability in Δ*rss03* was 2.0-fold lower than that in WT (Figure 4c), which provides evidence supporting the contribution of rss03 to *glpF* mRNA stability. To confirm the function of the binding site on *glpF* mRNA stability, we employed a transcriptional *lacZ* fusion plasmid, containing either the complete or truncated *glpF* mRNA under a constitutive promoter (P*gyrA*) (Figure 4d). We observed that the β-galactosidase activity of *glpF-lacZ* in Δ*rss03* was significantly lower than that in WT (Figure 4e) at the stationary phase, indicating that rss03 enhances *glpF* mRNA stability. Furthermore, the β-galactosidase activity of *glpF*-353 nt-*lacZ*, containing the interaction region, was significantly lower in Δ*rss03* than in WT. In contrast, there was no significant difference of β-galactosidase activity of *glpF*-345 nt-*lacZ*, excluding the interaction region, between WT and Δ*rss03* (Figure 4e). Additionally, the β-galactosidase activity of *glpF*-300 nt-*lacZ* or *glpF*-345 nt-*lacZ* was significantly higher than that of *glpF-lacZ*, *glpF*-400 nt-*lacZ*, or *glpF*-353 nt-*lacZ* in Δ*rss03* (Figure 4e). These results strongly suggested that *glpF* mRNA stability depends on the interaction sites between rss03 and *glpF* mRNA.

### rss03 protects glpF mRNA from *in vitro* degradation by RNase J1

In Gram-positive bacteria, the major enzymes responsible for endoribonuclease and 5’−3‘ exoribonuclease activities include RNase III (a double-stranded RNA-specific endoribonuclease), RNase Y (a single-stranded RNA-specific endoribonuclease), and RNase J1 and RNase J2 (both 5’−3’ exoribonuclease and endoribonuclease activities) [44]. To identify which RNase is responsible for attacking *glpF* mRNA in the absence of rss03, we examined the half-life of *glpF* mRNA in strains lacking RNase III in the presence or absence of rss03. Deletion of the gene encoding RNase III (Δ*rnc*) had no significant impact on *glpF* mRNA half-life in the presence (25.8 min) or absence (13.6 min) of rss03 (1.9-fold, Figure 4f), which is similar to that between WT and Δ*rss03* (2.0-fold, Figure 4c). These results indicate that RNase III is not the RNase responsible for *glpF* mRNA turnover. Despite several attempts, we were unable to construct deletion mutants for RNase J1, RNase J2, or RNase Y, nor could we express the recombinant RNase Y protein. Thus, we conducted *in vitro* cleavage assays to assess the potential involvement of RNase J1, RNase J2, or RNase III in *glpF* mRNA stability. After the treatment with RNase III, there was no significant difference in *glpF* mRNA degradation with or without rss03 (Figure 4g and S9). Interestingly, rss03 showed significantly strong protection of *glpF* transcript from RNase J1 degradation, reducing 5’ monophosphate generation. Conversely, RNase J2 retained the ability to degrade rss03-bound *glpF* transcripts (Figure 4g and S9). The results suggested that rss03 protects *glpF* mRNA from degradation by RNase J1.

### rss03 and GlpF contribute to *S. suis* oxidative stress resistance and bacterial survival in macrophages

**Figure 5.**
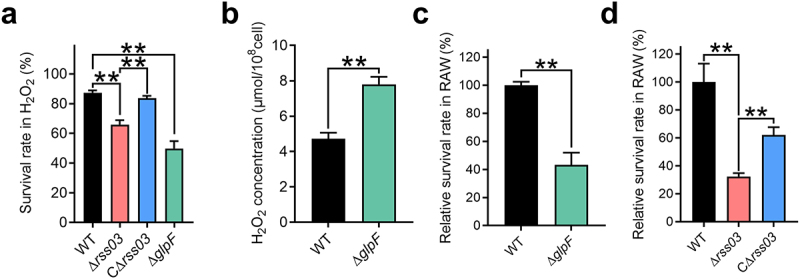
rss03 and GlpF contribute to *S. suis* oxidative stress resistance and bacterial survival in macrophages. (a) The survival rates of WT, Δ*rss03*, CΔ*rss03*, and Δ*glpF* under H_2_O_2_ stress condition. (b) The intracellular H_2_O_2_ content of WT and Δ*glpF* under H_2_O_2_ stress condition for 25 min. (c) The relative survival rate of WT and Δ*glpF* in macrophages RAW264.7. (d) The relative survival rate of WT, Δ*rss03*, and CΔ*rss03* in macrophages RAW264.7. Data represent the mean ± SEM, and asterisks indicate significantly different values (*n* = 3, ** indicates *p* < 0.01, two-tailed unpaired *t* test).

GlpF shares 34.07% amino acid identity with aquaporin Aagp, which promotes *S. suis* resistance to oxidative stress through H_2_O_2_ efflux [40]. Interestingly, we found that the survival rate of the Δ*glpF* was significantly lower than that of WT under H_2_O_2_ stress condition (Figure 5a). A similar phenotype was observed in Δ*rss03* compared with WT and CΔ*rss03* under H_2_O_2_ stress condition (Figure 5a). To investigate the involvement of GlpF in H_2_O_2_ diffusion, we measured the intracellular H_2_O_2_ concentration in Δ*glpF* under H_2_O_2_ stress conditions. The concentration of intracellular H_2_O_2_ in Δ*glpF* was significantly higher than that of WT (Figure 5b), indicating that GlpF, similar to Aagp, contributes to *S. suis* oxidative stress resistance by facilitating H_2_O_2_ efflux. Macrophages use reactive oxygen species (ROS) to eliminate pathogenic bacteria [45]. We found that the intracellular survival rate of Δ*glpF* in murine macrophages (RAW264.7) was significantly lower than that of the WT (Figure 5c). Similarly, Δ*rss03* showed a significantly reduced intracellular survival rate compared to WT and CΔ*rss03* (Figure 5d).

### rss03 is required for *S. suis* to maintain a high bacterial load in blood and brain

**Figure 6.**
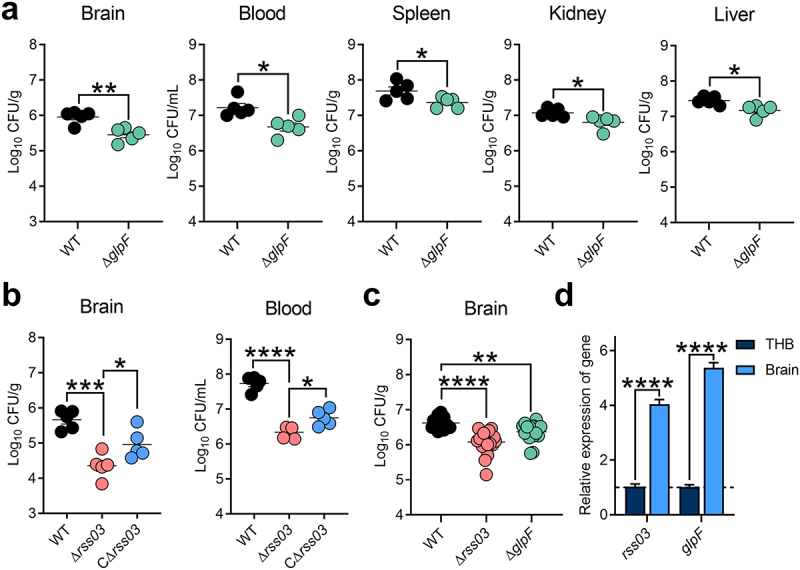
rss03 and *glpF* promote the survival of *S. suis* in the blood and bacterial load in the brain. (a) The bacterial load of WT and Δ*glpF* in the brain, blood, spleen, kidney, and liver after 12 h of intraperitoneal infection in mice (*n* = 5). (b) The bacterial load of WT, Δ*rss03*, or CΔ*rss03* in brain and blood after 12 h of intraperitoneal infection in mice (*n* = 5). (c) The bacterial load of WT, Δ*rss03*, and Δ*glpF* in the brain after 12 h of intracranial subarachnoidal infection in mice (*n* = 18). (d) Relative expression of *rss03* and *glpF* mRNA in brain after 12 h of intracranial subarachnoidal infection in mice compared to THB culture using RT-qPCR (*n* = 7). Data represent the mean ± SEM, and asterisks indicate significantly different values (* indicates *p* < 0.05, ** indicates *p* < 0.01, *** indicates *p* < 0.001, **** indicates *p* < 0.0001, two-tailed unpaired *t* test).

GlpF alleviates oxidative stress in *S. suis*, so we assessed its contribution to *S. suis* virulence by intraperitoneal infection in mice. Compared with WT infection groups, the number of Δ*glpF* in blood, brain, liver, spleen, and kidney was significantly reduced (Figure 6a). Since rss03 stabilizes the *glpF* transcript, we then investigated the role of rss03 in *S. suis* virulence using the same infection route. As shown in Figure 6b, compared with WT and CΔ*rss03* infection groups, the number of Δ*rss03* in blood and brain was significantly reduced, indicating that rss03 is required for *S. suis* to maintain a high bacterial load in blood and brain. However, the numbers of CFUs in mouse liver, spleen, and kidney were not significantly different when three infection groups were compared (Figure S10). We previously showed that *glpF* expression significantly increased during *S. suis* incubation with pig CSF [8] and its interaction with microglia, macrophage-like cells in the brain responsible for producing inflammation in response to invading pathogens [46]. To examine the roles of rss03 and GlpF on *S. suis* meningitis, we employed an intracranial subarachnoid route of infection in mice, which is suitable for investigating the mechanisms of *S. suis* meningitis [37]. As shown in Figure 6c, after 12 h of infection, the number of Δ*rss03* or Δ*glpF* in the brain was significantly lower compared to WT infection group. As anticipated, *rss03* and *glpF* mRNA expression were significantly higher in the brain compared to THB medium (Figure 6d).

## Discussion

The conservation of nucleotide sequence and genomic location among sRNA homologs across different species suggests that they may have similar functional roles. *Salmonella enterica* sRNA RfrA and *E. coli* sRNA RyhB, sharing 82% identity and encoded in the same genomic location, participate in iron availability [47]. Given the significant conservation of nucleotide sequence and genomic location of rss03 homologs among three streptococci (*S. suis*, *S. parasuis*, and *S. ruminantium*), it is likely that these sRNAs perform similar regulatory functions. Bacterial sRNAs from pathogens have been identified as potential biomarkers for infectious diseases [48]. rss03 from *S. suis* ST1 and ST7 strains shares 100% identity, and these two STs are responsible for human infections in China. We previously found that the expression of rss03 was significantly increased during *S. suis* incubation with pig CSF [8], and rss03 promotes *S. suis* survival in pig blood and virulence [8]. In this study, we have determined that rss03 is required for *S. suis* to maintain a high bacterial load in the blood and the brain. Therefore, sRNA rss03 could be a potential biomarker for *S. suis* pathogenesis.

Due to the diverse strengths of different approaches, combining multiple methods can effectively uncover the targets of sRNA [39]. In this study, we applied three approaches to identify the targetome of sRNA rss03 from an *S. suis* ST7 strain. Notably, MAPS identified a significantly higher number of verified targets of sRNA rss03 in *S. suis* compared to proteomics and CopraRNA. MAPS utilizes interest sRNA as baits, relying on the strong affinity between the MS2 RNA aptamer and the MS2 protein [49]. Consequently, the advantages of MAPS include its high sensitivity to low-expression targets, the ability to distinguish between direct and indirect targets, as well as the screening of base-paired sRNA targets [38]. MAPS was initially applied to sRNAs in *E. coli* [49] and has been successfully employed to effectively unveil the targetome of sRNAs in other Gram-negative bacteria [50,51]. However, among Gram-positive bacteria, MAPS has only been utilized in *S. aureus* [52]. In this study, we successfully applied MAPS to obtain the first targetome of *S. suis* sRNA, enabling us to understand sRNA regulatory mechanisms in *S. suis*.

sRNA rss03 enhances the stability of *glpF* mRNA through base-pairing interaction. In *S. suis* ST1 and ST7 strains, which show conservation of the sRNA rss03 homologs, the direct target *glpF* and its interaction region with rss03 are also conserved (Table S9). This suggests that the regulatory mechanism may be present across all *S. suis* ST1 and ST7 strains. So far, only a few cases have been reported in which sRNA promotes mRNA stability directly in Gram-positive bacteria. In *S. pyogenes*, sRNA FasX stabilizes *ska* mRNA by binding to its 5’ end, but it is not known which nuclease is involved [53]. In *Bacillus subtilis*, sRNA RoxS disrupts the exoribonuclease activity of RNase J1 by binding to the 5‘ end of *yflS* mRNA, increasing its stability [54]. Notably, we discovered that rss03 inhibits the exoribonuclease activity of RNase J1, effectively preventing the degradation of *glpF* transcripts. However, this protective effect is not observed with RNase J2. In *B. subtilis*, RNase J1 and RNase J2 were shown to have both endoribonuclease activity [55] and 5’-to-3‘ exoribonuclease [56]. However, RNase J1 exhibits stronger exoribonuclease activity than RNase J2 [56]. In *S. mutans*, an RNA *irvA* riboregulatory domain interacts with the coding region of target *gbpC* mRNA, sequesters RNase J2 cleavage site, and protects *gbpC* mRNA from degradation by RNase J2 [57]. In *S. suis* strain SC070731, RNase J1 shares only 37.23% of the amino acid identity with RNase J2. The *glpF*-400 nt transcript displays a predicted stem-loop secondary structure at the very 5’ end (Figure S11). This structural feature is similar to the *aprE* mRNA in *B. subtilis* [58], which may act as a barrier protecting mRNA from 5’-exonucleolytic degradation by RNase J1. Therefore, it is speculated that in the absence of rss03, RNase J1 may exhibit endoribonuclease activity, leading to a rearrangement of the secondary structure of the mRNA fragment. This rearrangement enables RNase J1 to access the 5’ end of *glpF* mRNA. Conversely, rss03 binding may interfere with RNase J1 endoribonuclease activity, allowing *glpF* mRNA to maintain a stable stem-loop structure, thereby protecting it from degradation by RNase J1 exoribonuclease activity.

*S. suis* GlpF is an aquaporin belonging to the glycerol-transporting aquaglyceroporins (AQGPs) subfamily, characterized by the WG(F/Y)R substrate selectivity motif. This distinguishes it from the atypical AQGPs with YVPR motif and water-transporting aquaporins (AQPs) with F(I/H)XR motif (Figure S7). AQGPs have exhibited diverse substrates, including glycerol and H_2_O_2_ [59]. Recently, we discovered that Aagp, an aquaporin capable of exporting H_2_O_2_ and transporting glycerol [40], shares 34.07% amino acid identity with GlpF. In *Lactobacillus plantarum*, three aquaporins (GlpF2, GlpF3, and GlpF4) were found to permeate H_2_O_2_ and glycerol, with amino acid identity to GlpF of 42.49%, 57.76%, and 38.63%, respectively [59]. In this study, we showed that GlpF contributes to oxidative stress resistance by facilitating H_2_O_2_ efflux and *S. suis* virulence. Similarly, the deletion of *Aagp* led to a significant reduction in the bacterial load of *S. suis* in various organs of mice, including the blood, brain, spleen, liver, and kidneys [40]. In *Vibrio alginolyticus*, the virulence regulatory sRNA Vvrr1 indirectly inhibits *glpF*, reducing biofilm formation and adhesion [60]. *Listeria monocytogenes* GlpF has also been implicated in the biofilm formation [61] and adhesion capacity [62]. We previously showed that the expression of *glpF* was significantly increased during *S. suis* incubation with pig CSF [8] and its interaction with microglia [46]. Macrophages and neutrophils, abundant in tissues and blood, produce ROS to eliminate pathogens [45,63]. During meningitis, many neutrophils typically infiltrate the brain [64]. Thus, we assume that GlpF promotes *S. suis* maintaining a high bacterial load in blood and brain by resistance to oxidative stress.

In this study, we identified 14 direct targets of rss03 (Table 1). In addition to *glpF*, we predicted the binding regions of sRNA rss03 with other direct targets (Figure S12). We found that, aside from potential binding to the RBS region of *araE* and the 5’ UTR region of *xerD*, rss03 primarily binds to the coding region of its direct targets (12 out of 14). Furthermore, we identified that rss03 contains a G/U-rich element (5’-GUGGGGGG-3’) (Figure S12) that may interact with most of its mRNA targets (9 out of 14). Therefore, we speculate that this region likely serves as the seed region of sRNA rss03. RNA modifications serve versatile functions in pathogenic bacteria [65]. The rRNA pseudouridine synthase A (RsuA) is responsible for the singular pseudouridine modification located at position 516 in *E. coli* 16S RNA [66]. RsuA is crucial in bacterial survival under various stress conditions [67] and antibiotic resistance [68]. In this study, *rsuA* is the rss03 direct target (Figure S5a), and RsuA shares a 32.62% amino acid identity with its homologs in *E. coli*. Consequently, it is conceivable that the rss03 may influence the rRNA modification of *S. suis* by regulating *rsuA*. Another direct target gene of rss03, *usp*, encodes a universal stress protein. We previously revealed a significant upregulation of *usp* expression in CSF, indicating its potential role in alleviating stress when *S. suis* transitions from a nutrient-rich environment to a nutrient-deprived condition [8]. Therefore, it can be hypothesized that rss03 may regulate the expression of *usp* and contribute to *S. suis* coping with various environmental stresses. Sialic acid is an important carbon source for pathogens [69]. Sialic acid is a capsule component of SS2 strains, and it has been shown to play a role in both virulence and resistance to phagocytosis [70]. NanR, a RpiR family transcriptional regulator, regulates the genes encoding enzymes required for sialic acid transport and metabolism [71]. In *S. suis*, NanR shares 48.86% and 29.72% amino acid identity with its homologs in *S. pneumoniae* [71] and *C. perfringens* [72], respectively. In this study, *nanR* is also the rss03 direct target (Figure S5a). Therefore, it is worth investigating whether rss03 is involved in the transport and metabolism of sialic acid in *S. suis* through the regulation of the NanR regulator, thereby enhancing its survival and virulence. The peptidoglycan, a major component of the cell wall, is composed of alternating N-acetylglucosamine (GlcNAc) and N-acetylmuramic acid (MurNAc)-pentapeptide linked by β-(1→4) glycosidic linkages. Disrupting peptidoglycan recycling can attenuate the virulence of pathogens [73]. In *E. coli*, the transcriptional repressor MurR controls the expression of *murP* and *murQ*, which transport and metabolize MurNAc during the cell wall recycling process [74]. In this study, rss03 directly binds to *murR* mRNA. Therefore, it is worth investigating whether rss03 is involved in *S. suis* cell wall recycling through the regulation of the MurR transcriptional regulator, thereby influencing its pathogenicity. In addition, two target genes of rss03, *xerD,* and *xerC*, encode site-specific tyrosine recombinases. In some pathogens, XerC and XerD are involved in the cutting and integrating horizontally transferred DNA [75,76]. Therefore, it is hypothesized that rss03 may regulate the expression of target genes *xerD* and *xerC*, potentially influencing the adaptability and evolution of *S. suis* through efficient integration of horizontally acquired DNA. Additionally, sRNA rss03 may impact the pathogenicity of *S. suis* by regulating target *ssnA* mRNA (Figure S5b), which encodes the deoxyribonuclease. SsnA has been reported to facilitate the evasion of neutrophil extracellular traps in *S. suis* [77] and play a key role in *S. suis* pathogenesis [78].

**Figure 7.**
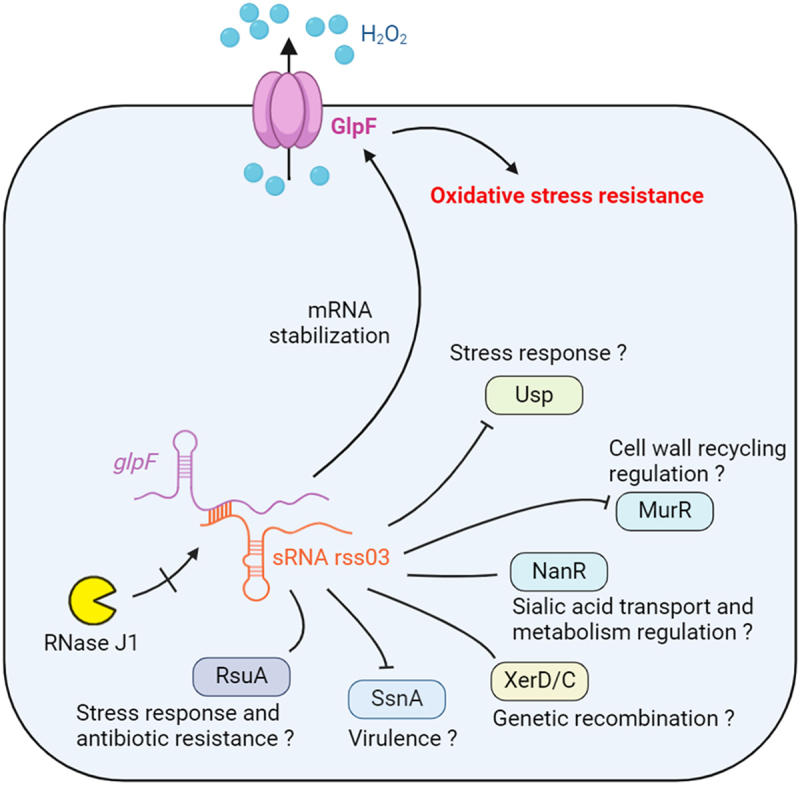
The regulatory networks involving rss03 and its mRNA targets. rss03 promotes the mRNA stability of its direct target, *glpF*, which encodes an aquaporin responsible for H_2_O_2_ efflux. Both rss03 and GlpF enhance *S. suis* oxidative stress resistance and survival in murine macrophages, sustaining an elevated bacterial load within the blood and brain. Moreover, by regulating its direct targets, rss03 potentially influences rRNA modification, antibiotic resistance, virulence, stress response, sialic acid transport and metabolism, cell wall recycling, and gene recombination. The arrows and T-shaped lines represent direct positive and negative regulation, respectively, according to proteomic analysis.

In this study, we employed the MAPS approach for the first time in *S. suis* to identify the direct targets of sRNA. Unexpectedly, rss03 protects *glpF* mRNA from degradation by RNase J1, and both rss03 and GlpF are required to maintain a high bacterial load in mouse blood and brain. However, the mechanism of the protection of *glpF* transcript from RNase J1 degradation by rss03 and the function of other direct targets remain to be investigated (Figure 7).

## Supplementary Material

Figure S4.tif

Figure S3.tif

Table S1.docx

Table S6.docx

Figure S5.tif

Table S8.docx

Figure S10.tif

Table S4.docx

Table S7.docx

Figure S11.tif

Table S2.docx

Figure S1.tif

Figure S9.tif

Figure S8.tif

Table S9.docx

Table S5.docx

Figure S6.tif

Table S3.docx

Figure S2.tif

Supplementary Figure Legends.docx

Figure S12.tif

Figure S7.tif

The ARRIVE Guidelines Checklist.pdf

## Data Availability

The mass spectrometry proteomics data were deposited in the ProteomeXchange Consortium (https://proteomecentral.proteomexchange.org) via the iProX partner repository with the dataset identifier PXD050638. The MAPS data were deposited in the NCBI SRA database with accession numbers SRR28341118, SRR28341119, SRR28341120, and SRR28341121 under the BioProject accession number PRJNA1087569. The data supporting the findings of this study are available at Figshare (https://doi.org/10.6084/m9.figshare.26490628).
