# Supplementary figures and images for: A small RNA from Streptococcus suis epidemic ST7 strain promotes bacterial survival in host blood and brain by enhancing oxidative stress resistance
Source: Virulence. 2025 Apr 16;16(1):2491635. doi: 10.1080/21505594.2025.2491635 (PMC12005413; doi:10.1080/21505594.2025.2491635)

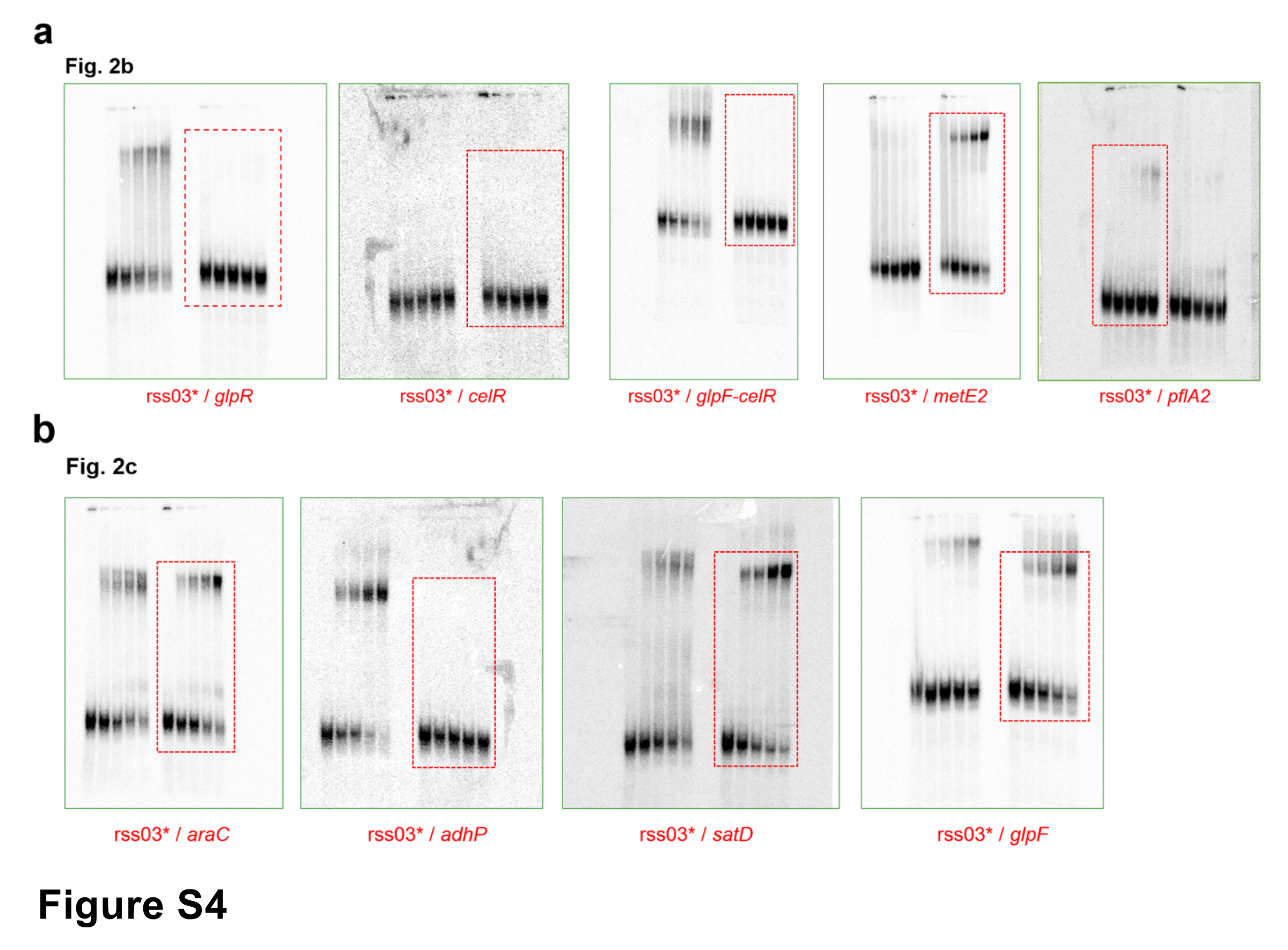

Supplement: Figure S4.tif [file KVIR_A_2491635_SM4079.tif]

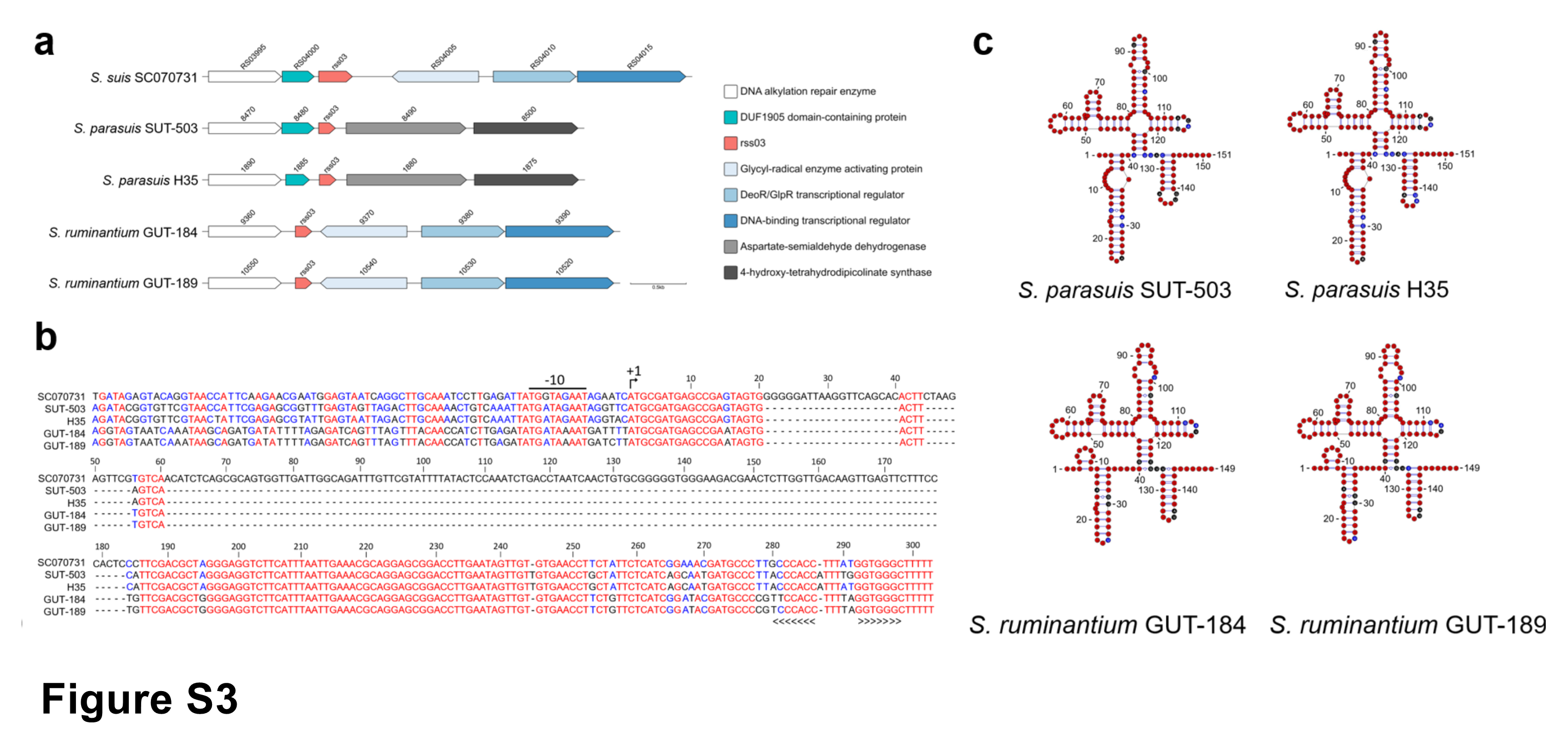

Supplement: Figure S3.tif [file KVIR_A_2491635_SM4078.tif]

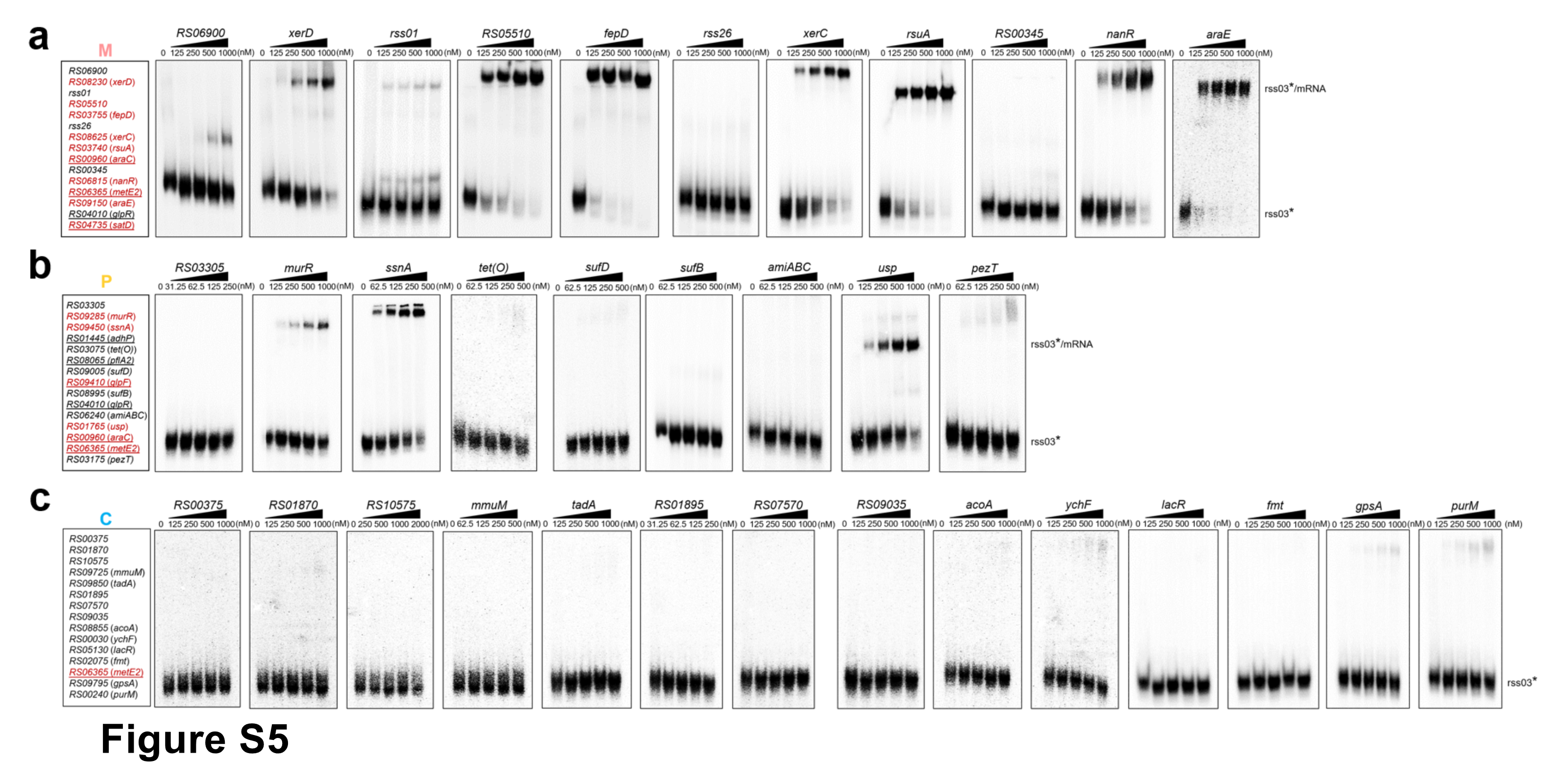

Supplement: Figure S5.tif [file KVIR_A_2491635_SM4075.tif]

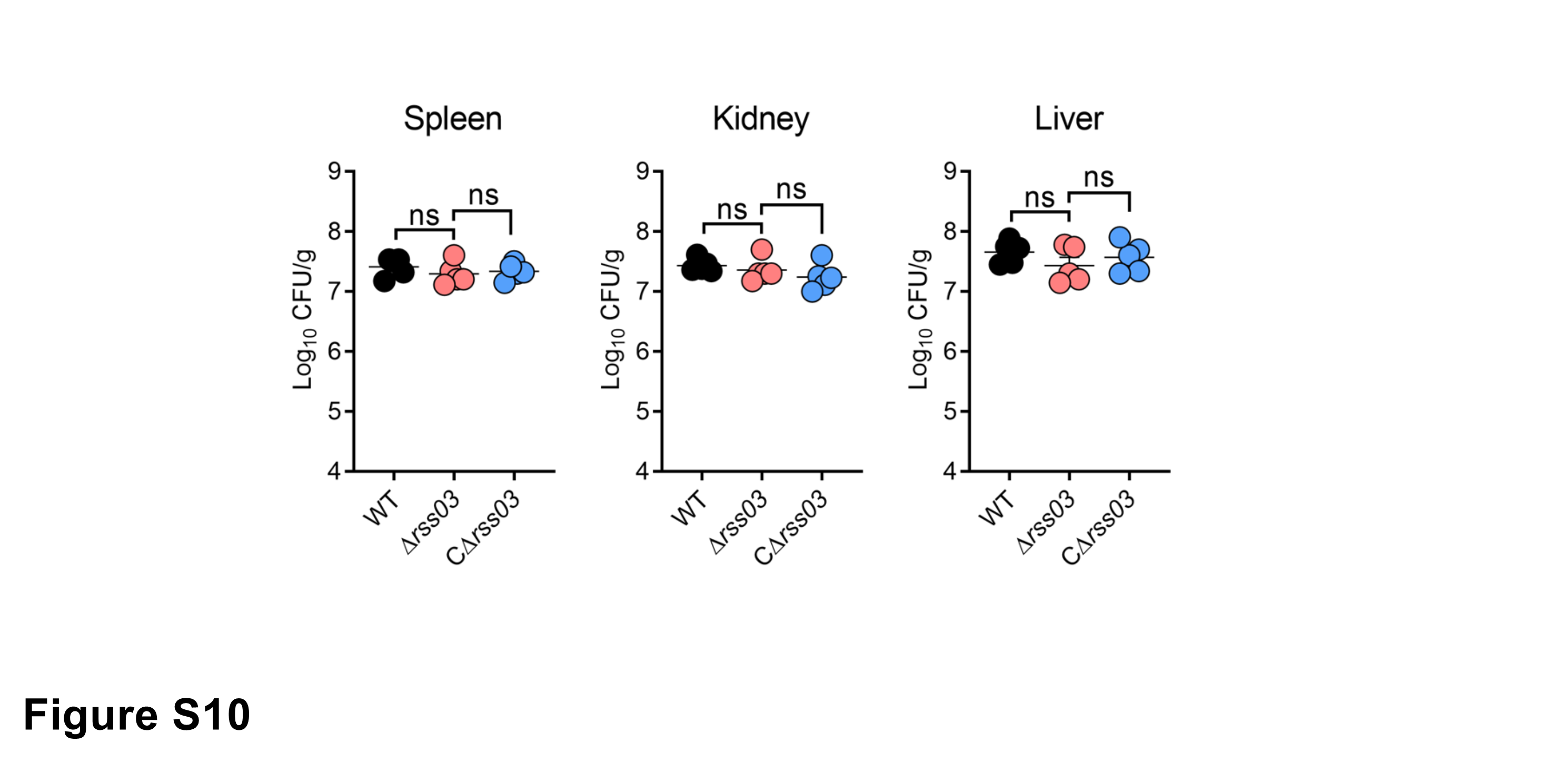

Supplement: Figure S10.tif [file KVIR_A_2491635_SM4073.tif]

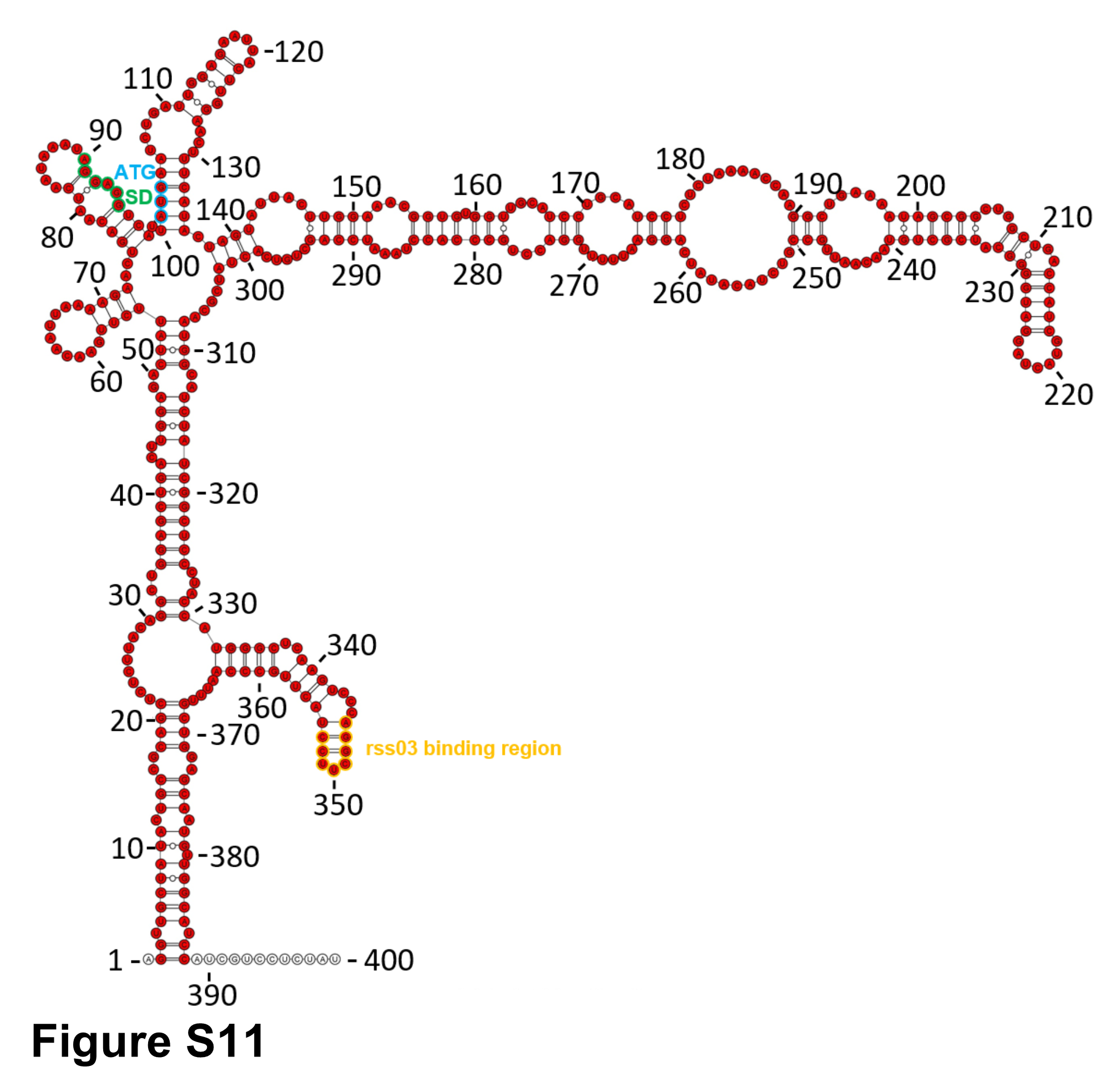

Supplement: Figure S11.tif [file KVIR_A_2491635_SM4070.tif]

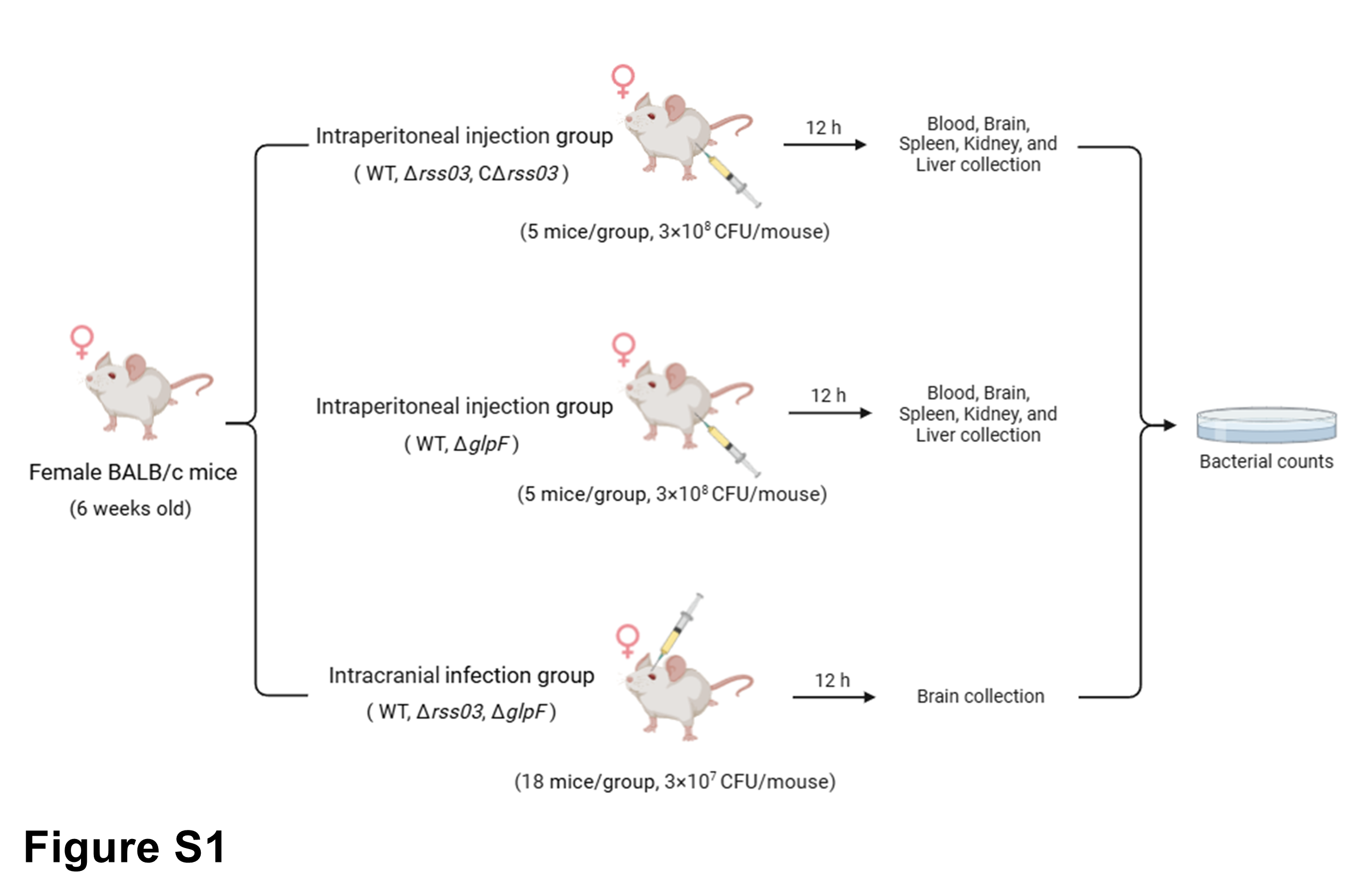

Supplement: Figure S1.tif [file KVIR_A_2491635_SM4068.tif]

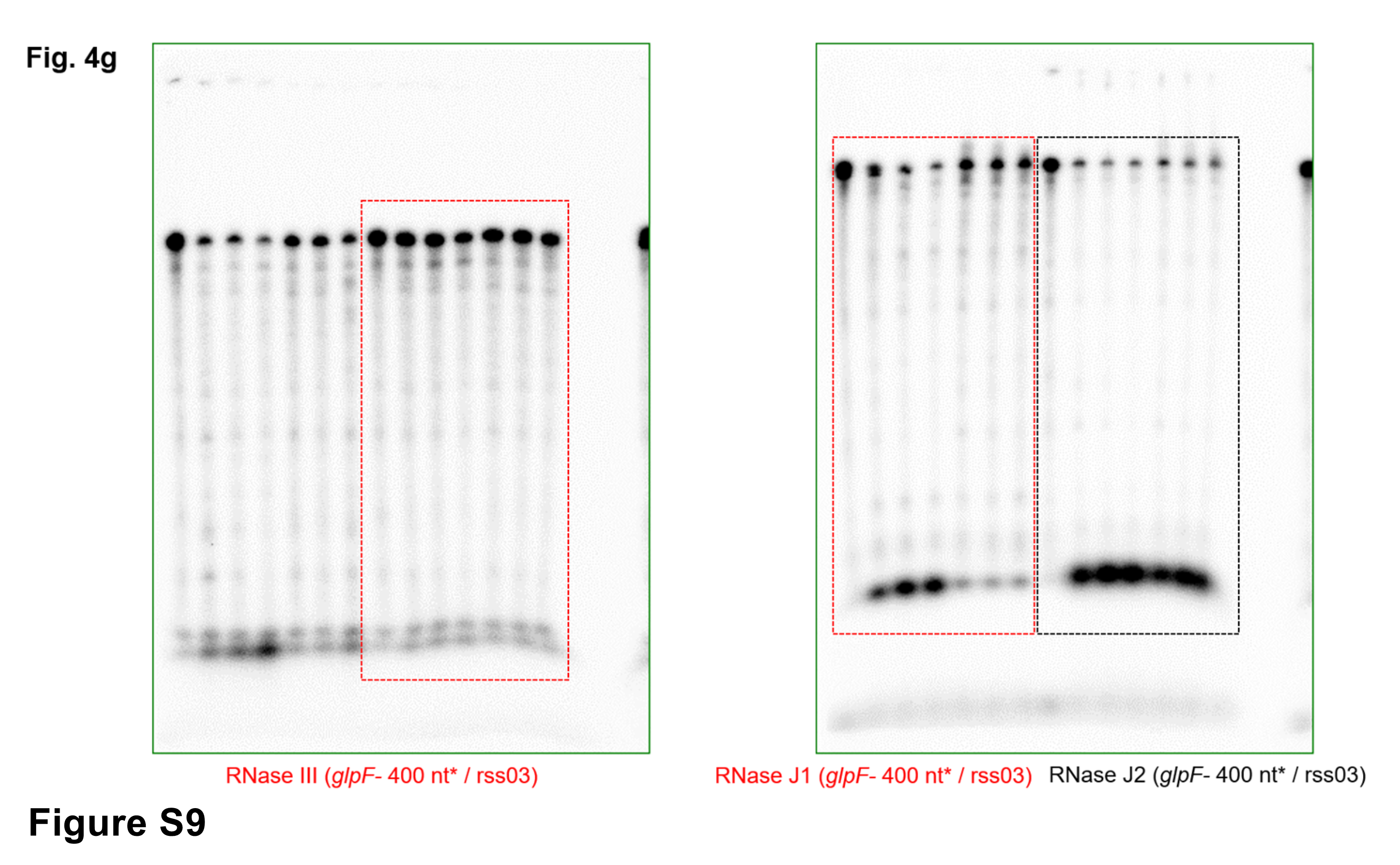

Supplement: Figure S9.tif [file KVIR_A_2491635_SM4067.tif]

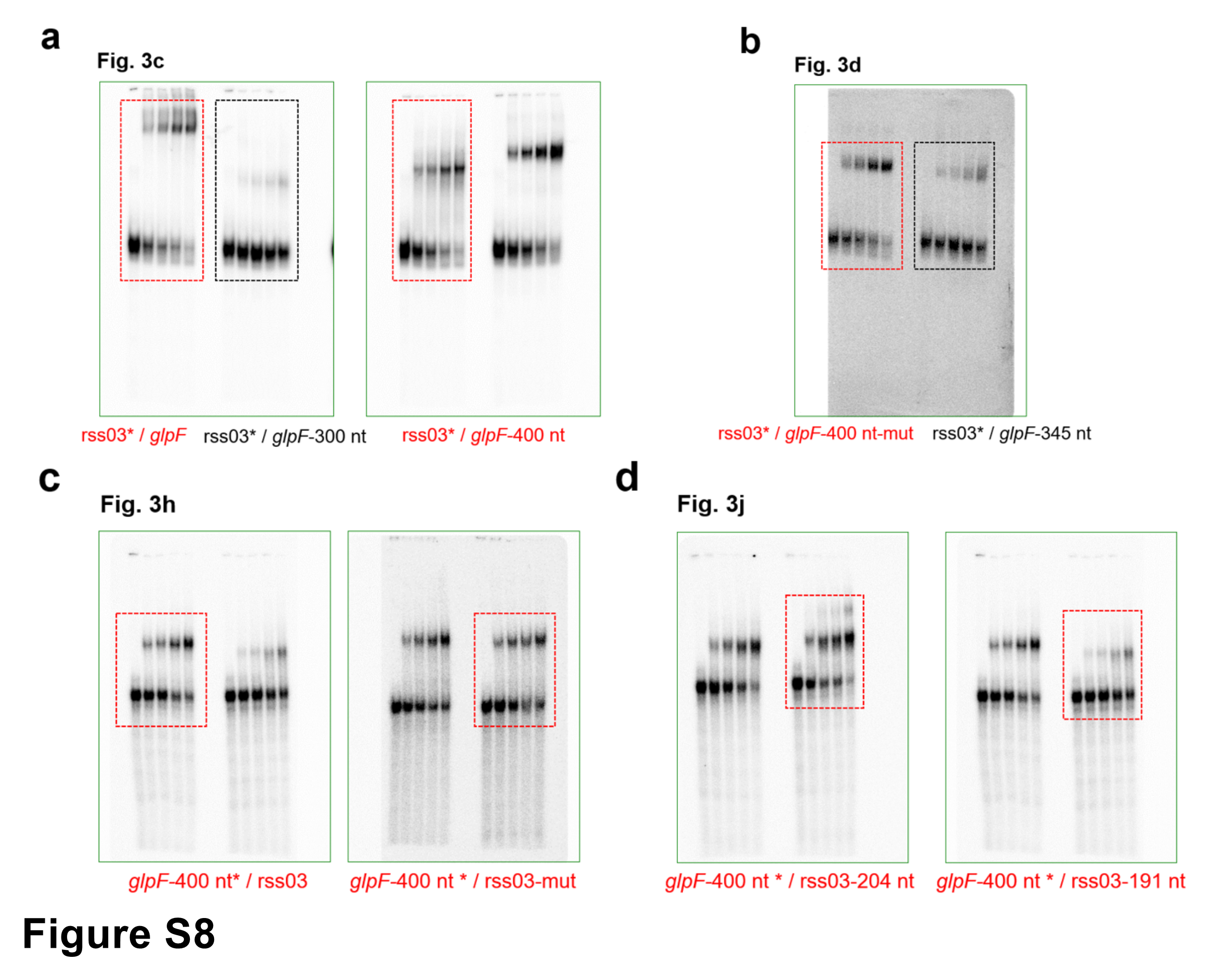

Supplement: Figure S8.tif [file KVIR_A_2491635_SM4066.tif]

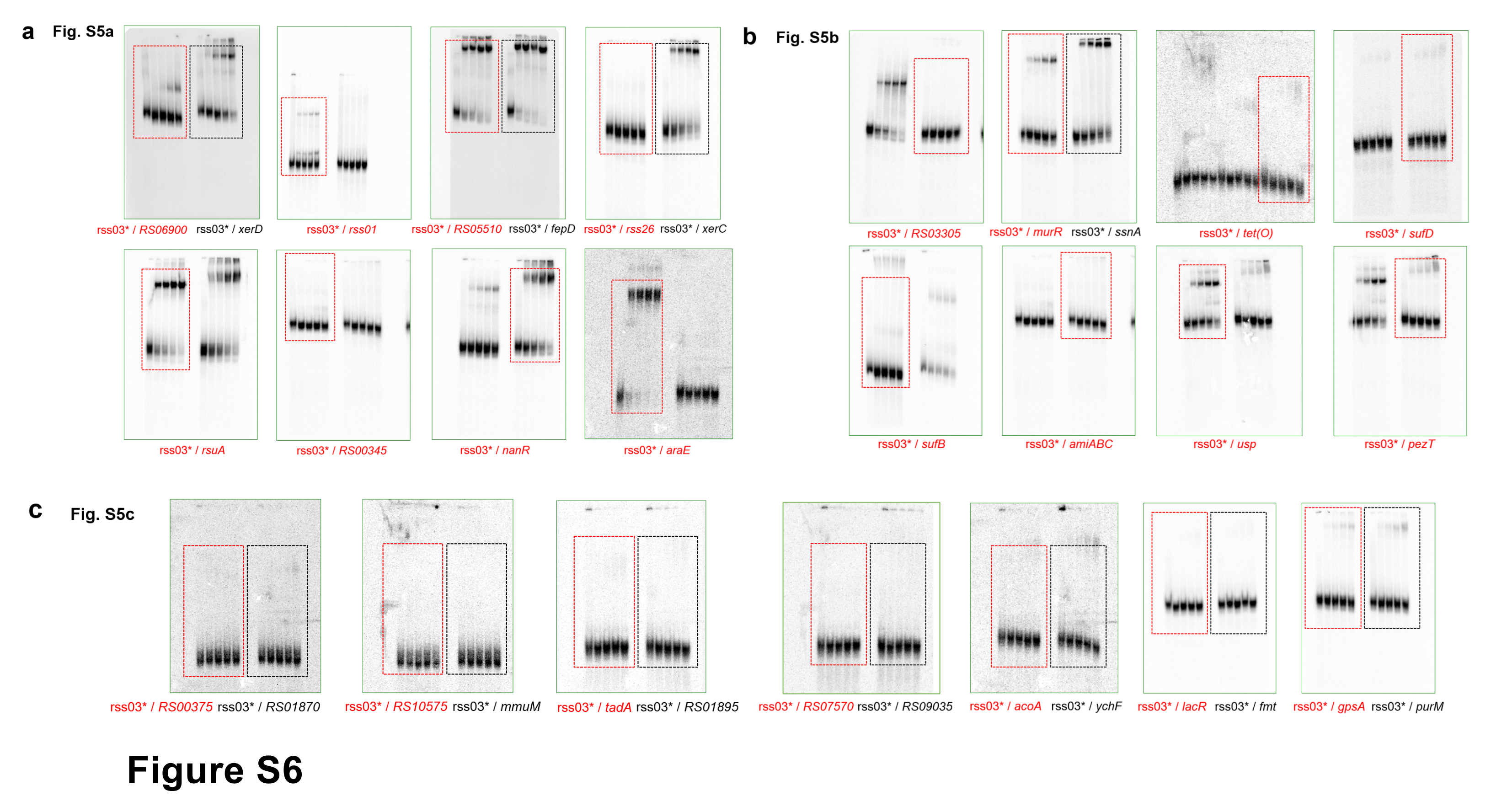

Supplement: Figure S6.tif [file KVIR_A_2491635_SM4063.tif]

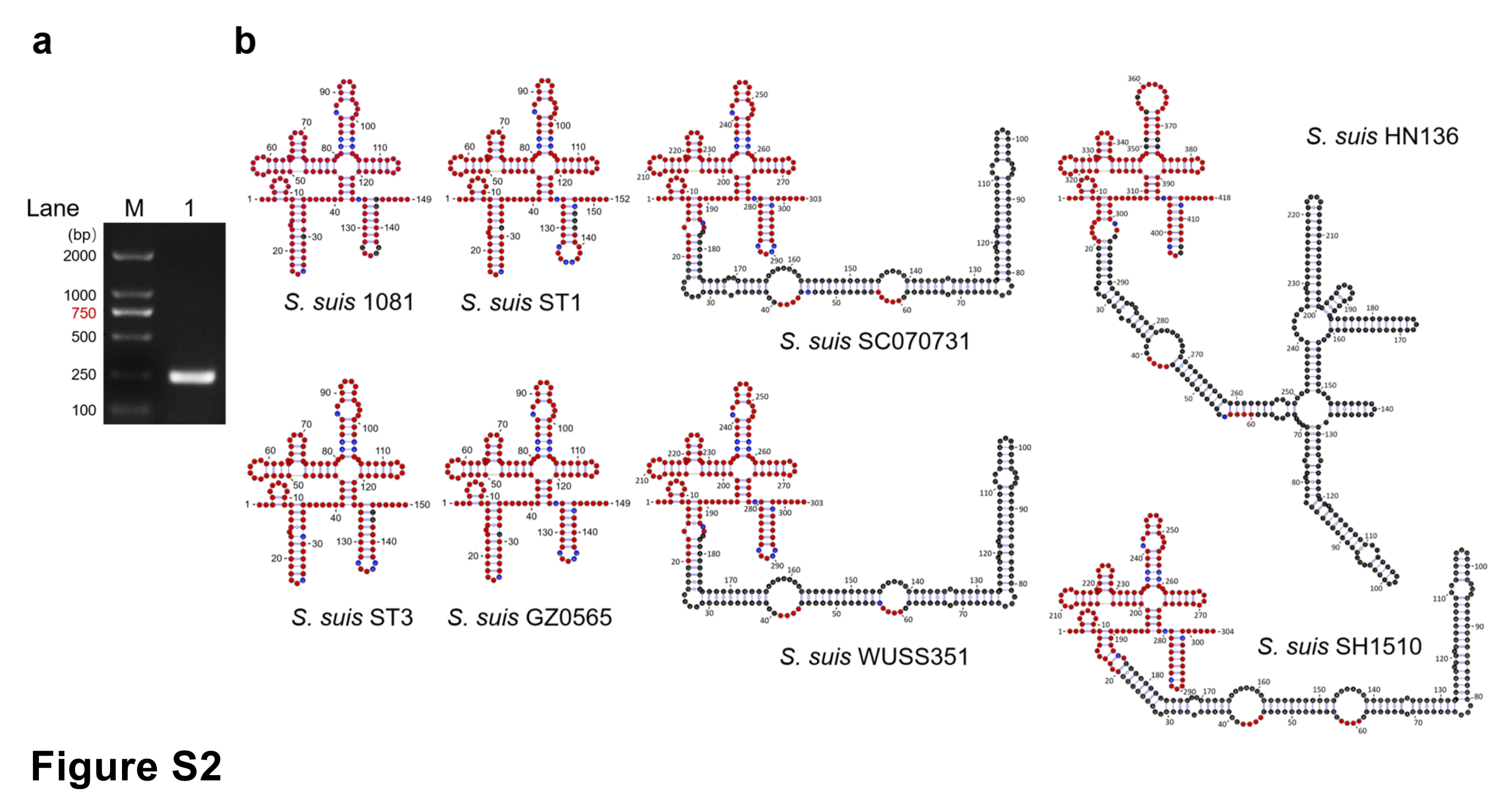

Supplement: Figure S2.tif [file KVIR_A_2491635_SM4061.tif]

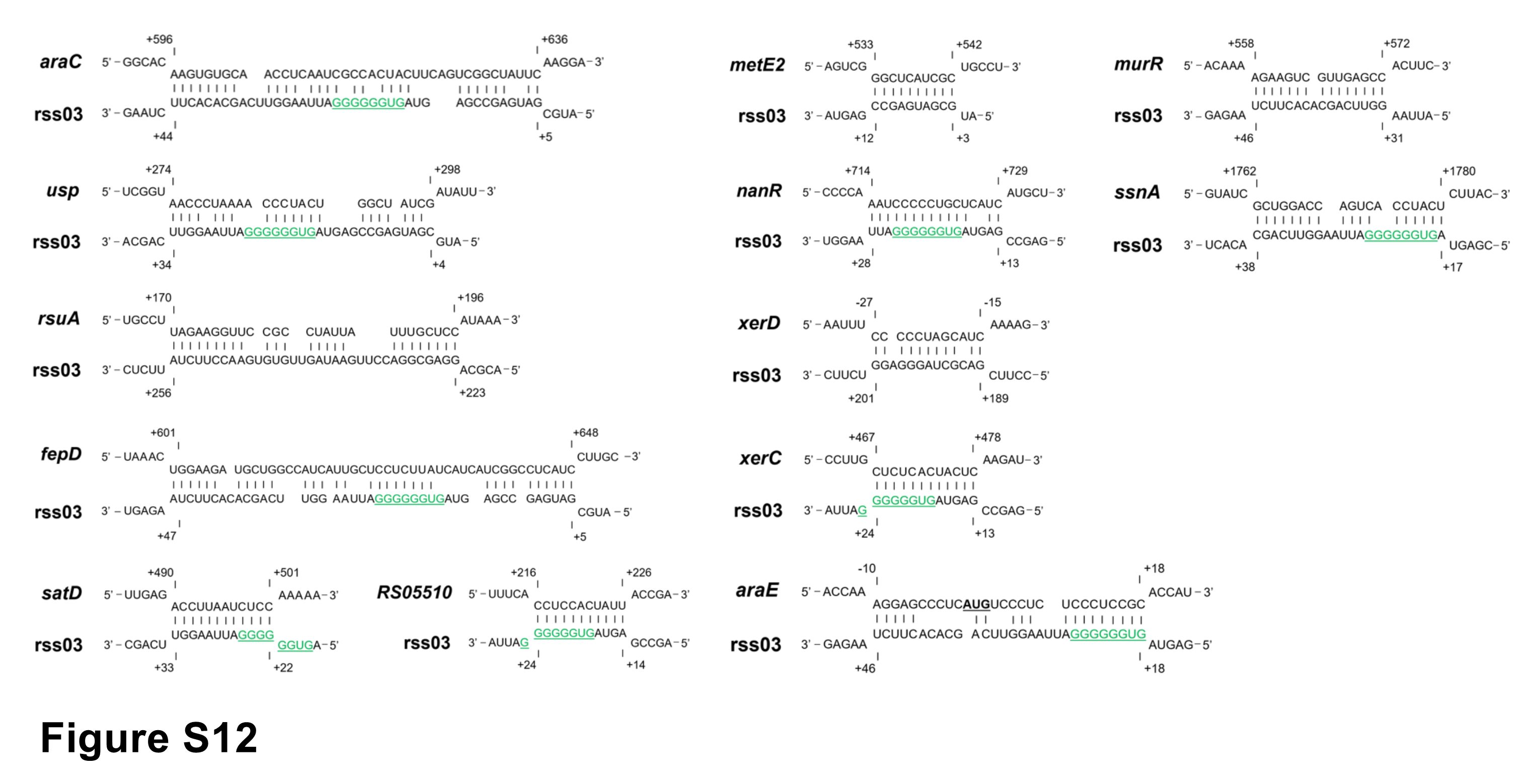

Supplement: Figure S12.tif [file KVIR_A_2491635_SM4059.tif]

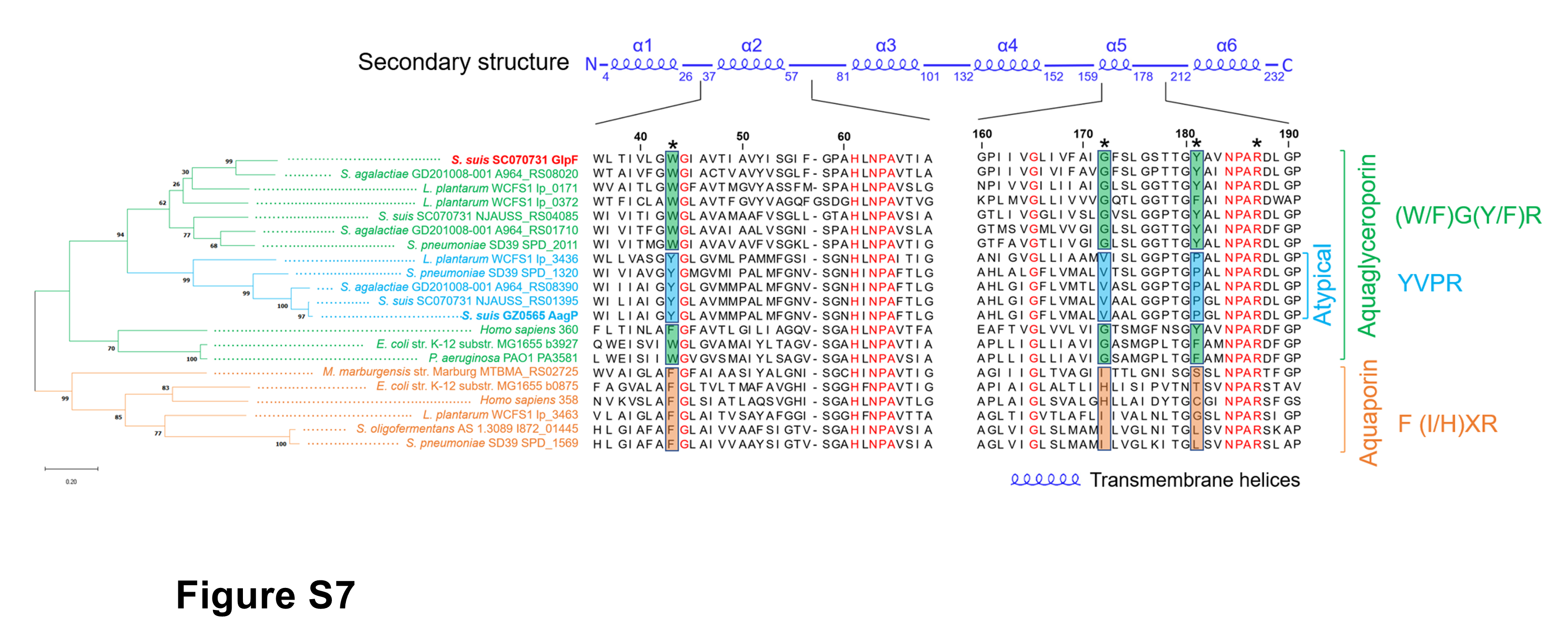

Supplement: Figure S7.tif [file KVIR_A_2491635_SM4058.tif]
