# Supplementary material for: A small RNA from Streptococcus suis epidemic ST7 strain promotes bacterial survival in host blood and brain by enhancing oxidative stress resistance
Source: Virulence. 2025 Apr 16;16(1):2491635. doi: 10.1080/21505594.2025.2491635 (PMC12005413; doi:10.1080/21505594.2025.2491635)
Supplement: Table S1.docx [file KVIR_A_2491635_SM4077.docx]

# Table S1. Strains and plasmids used in this study.

| **Strains or Plasmids** | **Characteristic(s)** | **Source** |
| --- | --- | --- |
| **Strains** | | |
| SC070731 | *S. suis* serotype 2 virulent strain SC070731 was isolated from a diseased pig in China | [[1](#_ENREF_1)] |
| Δ*rss03* | Deletion of 810,520-810,822 fragment in SC070731 strain background | This study |
| CΔ*rss03* | *S. suis* strain Δ*rss03* containing pSET2-rss03 plasmid | This study |
| WT-pSET2-MS2-rss03 | *S. suis* strain SC070731 containing pSET2-MS2-rss03 plasmid | This study |
| Δ*rss03*-pSET2-MS2-negative | *S. suis* strain Δ*rss03* containing pSET2-MS2-negative plasmid | This study |
| WT-pTCV-P*gyrA*-*glpF* | *S. suis* strain SC070731 containing pTCV-P*gyrA*-*glpF* plasmid | This study |
| WT-pTCV-P*gyrA*-*glpF*-345 nt | *S. suis* strain SC070731 containing pTCV-P*gyrA*-*glpF*-345 nt plasmid | This study |
| WT-pTCV-P*gyrA*-*glpF*-353 nt | *S. suis* strain SC070731 containing pTCV-P*gyrA*-*glpF*-353 nt plasmid | This study |
| Δ*rss03-*pTCV-P*gyrA*-*glpF* | *S. suis* strain Δ*rss03* containing pTCV-P*gyrA*-*glpF* plasmid | This study |
| Δ*rss03-*pTCV-P*gyrA*-*glpF*-300 nt | *S. suis* strain Δrss03 containing pTCV-P*gyrA*-*glpF*-300 nt plasmid | This study |
| Δ*rss03-*pTCV-P*gyrA*-*glpF*-345 nt | *S. suis* strain Δ*rss03* containing pTCV-P*gyrA*-*glpF*-345 nt plasmid | This study |
| Δ*rss03-*pTCV-P*gyrA*-*glpF*-353 nt | *S. suis* strain Δ*rss03* containing pTCV-P*gyrA*-*glpF*-353 nt plasmid | This study |
| Δ*rss03-*pTCV-P*gyrA*-*glpF*-400 nt | *S. suis* strain Δ*rss03* containing pTCV-P*gyrA*-*glpF*-400 nt plasmid | This study |
| Δ*rnc* | Deletion of *NJAUSS_RS05775* in SC070731 strain background | This study |
| Δ*rss03*Δ*rnc* | Deletion of *NJAUSS_RS05775* in Δ*rss03* strain background | This study |
| Δ*glpF* | Deletion of *NJAUSS_RS09410* in SC070731 strain background | This study |
| BL21 | *E. coli* strain for protein expression from the recombinant plasmids | Vazyme |
| BL21-pET28a-*rnc* | *E. coli* strain BL21 containing pET28a-*rnc* plasmid | This study |
| BL21-pET28a-*rnj1* | *E. coli* strain BL21 containing pET28a-*rnj1* plasmid | This study |
| BL21-pET28a-*rnj2* | *E. coli* strain BL21 containing pET28a-*rnj2* plasmid | This study |
| DH5α | *E. coli* strain for maintaining the recombinant plasmids | Vazyme |
| **Plasmids** | | |
| pSET2 | *S. suis* / *E. coli* shuttle plasmid, Spc^r^ | Daisuke Takamatsu |
| pSET2-*rss03* | pSET2 carrying *rss03* gene with rss03 promoter, Spc^r^ | This study |
| pMBP-MS2 | A plasmid used to express fusion protein MS2-MBP, Amp^r^ | Pascale Romby |
| pSET2-MS2-*rss03* | pSET2 carrying MS2 and *rss03* gene with rss03 promoter, Spc^r^ | This study |
| pSET2-MS2-negative | pSET2 carrying MS2 with rss03 promoter, Spc^r^ | This study |
| pTCV | A plasmid with *lacZ* for the construction of transcriptional fusions, Spc^r^ | [[2](#_ENREF_2)] |
| pTCV-P*gyrA*-*glpF*-300 nt | pTCV carrying 1,901,801-1,902,100 fragments with *gyrA* promoter, Spc^r^ | This study |
| pTCV-P*gyrA*-*glpF*-345 nt | pTCV carrying 1,901,801-1,902,145 fragments with *gyrA* promoter, Spc^r^ | This study |
| pTCV-P*gyrA*-*glpF*-353 nt | pTCV carrying 1,901,801-1,902,153 fragments with *gyrA* promoter, Spc^r^ | This study |
| pTCV-P*gyrA*-*glpF*-400 nt | pTCV carrying 1,901,801-1,902,400 fragments with *gyrA* promoter, Spc^r^ | This study |
| pTCV-P*gyrA*-*glpF* | pTCV carrying 1,901,801-1,902,611 fragments with *gyrA* promoter, Spc^r^ | This study |
| pET28a | A plasmid used to express a heterologous protein, His tag, Kan^r^ | Novagen |
| pET28a-*rnc* | pET28a carrying *NJAUSS_RS05775*, His tag, Kan^r^ | This study |
| pET28a-*rnj1* | pET28a carrying *NJAUSS_RS00935*, His tag, Kan^r^ | This study |
| pET28a-*rnj2* | pET28a carrying *NJAUSS_RS07180*, His tag, Kan^r^ | This study |
| pMD 19-T | A plasmid used to clone PCR products, Amp^r^ | Takara |
| pMD 19-T-rss03-3' | pMD 19-T carrying nested PCR product of 3' end of rss03, Amp^r^ | This study |

Spc^r^, spectinomycin resistance cassette. Kan^r^, kanamycin-resistant cassette. Amp^r^, ampicillin-resistant cassette.

# References

1. Wu ZF, Wang WX, Tang M, et al. Comparative genomic analysis shows that Streptococcus suis meningitis isolate SC070731 contains a unique 105 K genomic island. Gene. 2014 Feb 10;535(2):156-164.

2. Poyart C, Trieu-Cuot P. A broad-host-range mobilizable shuttle vector for the construction of transcriptional fusions to beta-galactosidase in gram-positive bacteria. Fems Microbiol Lett. 1997 Nov 15;156(2):193-8.
