# Supplementary material for: A small RNA from Streptococcus suis epidemic ST7 strain promotes bacterial survival in host blood and brain by enhancing oxidative stress resistance
Source: Virulence. 2025 Apr 16;16(1):2491635. doi: 10.1080/21505594.2025.2491635 (PMC12005413; doi:10.1080/21505594.2025.2491635)
Supplement: Table S6.docx [file KVIR_A_2491635_SM4076.docx]

# Table S6. The candidate targets obtained by MAPS.

| **Number** | **Gene_ID** | **Annotation** | **Log_2_FC*** | ***p*-value** |
| --- | --- | --- | --- | --- |
| 1 | NJAUSS_RS06900 | hypothetical protein | 6.301921206 | 0.016617377 |
| 2 | rss03 | sRNA detected from our previous work [[4](#_ENREF_4)] | 5.942484412 | 2.19E-12 |
| 3 | NJAUSS_RS08230 | site-specific tyrosine recombinase XerD | 4.493779709 | 1.74E-07 |
| 4 | rss01 | sRNA detected from our previous work [[4](#_ENREF_4)] | 3.900473596 | 0.007791085 |
| 5 | NJAUSS_RS05510 | hypothetical protein | 3.844878592 | 1.62E-05 |
| 6 | NJAUSS_RS03755 | iron ABC transporter permease | 3.771644469 | 0.003398776 |
| 7 | rss26 | sRNA detected from our previous work [[4](#_ENREF_4)] | 3.757046997 | 2.42E-06 |
| 8 | NJAUSS_RS08625 | site-specific tyrosine recombinase XerC | 3.714357372 | 0.047226524 |
| 9 | NJAUSS_RS03740 | rRNA pseudouridine synthase | 3.47769815 | 0.000279763 |
| 10 | NJAUSS_RS00960 | AraC family transcriptional regulator | 3.459728387 | 0.007120979 |
| 11 | NJAUSS_RS00345 | IS4/IS5 family transposase | 3.340996947 | 0.01111271 |
| 12 | NJAUSS_RS07095 | hypothetical protein | 3.285445526 | 0.02162869 |
| 13 | NJAUSS_RS03730 | low temperature requirement A protein | 3.230036849 | 0.0056625 |
| 14 | NJAUSS_RS06815 | RpiR family transcriptional regulator | 3.159807803 | 0.006335351 |
| 15 | NJAUSS_RS08500 | MATE family efflux transporter | 3.139149373 | 0.000465168 |
| 16 | NJAUSS_RS00750 | HIT family protein | 3.087155329 | 0.000258141 |
| 17 | NJAUSS_RS01815 | LacI family transcriptional regulator | 3.072325291 | 7.33E-05 |
| 18 | NJAUSS_RS08490 | potassium transporter Trk | 2.994474814 | 0.000880236 |
| 19 | NJAUSS_RS05530 | membrane protein | 2.989454055 | 0.02094843 |
| 20 | NJAUSS_RS08220 | segregation/condensation protein B | 2.977308323 | 0.000365874 |
| 21 | NJAUSS_RS01750 | asparaginase | 2.975437279 | 0.000560425 |
| 22 | NJAUSS_RS06570 | glutamine ABC transporter substrate-binding protein | 2.958883996 | 0.002624546 |
| 23 | NJAUSS_RS08485 | heptaprenyl diphosphate synthase subunit I | 2.952843379 | 0.008577649 |
| 24 | NJAUSS_RS10475 | copper ABC transporter permease | 2.904224262 | 0.000288813 |
| 25 | NJAUSS_RS05505 | DUF1700 domain-containing protein | 2.890476227 | 0.001959561 |
| 26 | NJAUSS_RS01590 | transcriptional repressor | 2.866143116 | 0.001577966 |
| 27 | NJAUSS_RS04435 | polysaccharide deacetylase family protein | 2.863613178 | 0.000723944 |
| 28 | NJAUSS_RS00350 | bifunctional folylpolyglutamate synthase/dihydrofolate synthase | 2.848219027 | 0.000830978 |
| 29 | NJAUSS_RS06430 | DUF3397 domain-containing protein | 2.77222774 | 0.013756845 |
| 30 | NJAUSS_RS06365 | 5-methyltetrahydropteroyltriglutamate-homocysteine methyltransferase | 2.720291094 | 0.001185009 |
| 31 | NJAUSS_RS05235 | peptidase | 2.718041165 | 0.019263893 |
| 32 | NJAUSS_RS05860 | hyaluronidase | 2.714338298 | 0.016160151 |
| 33 | NJAUSS_RS08830 | lipoate--protein ligase A | 2.669256052 | 0.00087273 |
| 34 | NJAUSS_RS09150 | aromatic acid exporter family protein | 2.584327979 | 0.005426565 |
| 35 | NJAUSS_RS04175 | CPBP family intramembrane metalloprotease | 2.556952273 | 0.000487848 |
| 36 | NJAUSS_RS04010 | DeoR/GlpR transcriptional regulator | 2.547682371 | 0.004853058 |
| 37 | NJAUSS_RS01375 | PadR family transcriptional regulator | 2.533855465 | 0.016850728 |
| 38 | NJAUSS_RS04735 | DNA-binding protein | 2.465719287 | 0.038345644 |
| 39 | NJAUSS_RS05310 | type I pantothenate kinase | 2.431017612 | 0.010963339 |
| 40 | NJAUSS_RS00455 | 30S ribosomal protein S19 | 2.39666855 | 0.007776554 |
| 41 | NJAUSS_RS05490 | permease | 2.394270047 | 0.047780331 |
| 42 | NJAUSS_RS01785 | 50S ribosomal protein L19 | 2.388186714 | 0.003255148 |
| 43 | NJAUSS_RS08430 | DUF3278 domain-containing protein | 2.368614485 | 0.032630086 |
| 44 | NJAUSS_RS05525 | threonine/serine exporter | 2.368598632 | 0.032615468 |
| 45 | NJAUSS_RS00240 | phosphoribosylformylglycinamidine cyclo-ligase | 2.327464986 | 0.012446155 |
| 46 | NJAUSS_RS01345 | membrane protein | 2.324701808 | 0.017411768 |
| 47 | NJAUSS_RS07275 | DUF1694 domain-containing protein | 2.321143394 | 0.02064237 |
| 48 | NJAUSS_RS07135 | DUF1727 domain-containing protein | 2.309368951 | 0.004978942 |
| 49 | NJAUSS_RS08455 | NusG domain II-containing protein | 2.291337314 | 0.005516621 |
| 50 | NJAUSS_RS02580 | DUF368 domain-containing protein | 2.25377573 | 0.002915184 |
| 51 | NJAUSS_RS08525 | DUF1398 domain-containing protein | 2.234424172 | 0.019887304 |
| 52 | NJAUSS_RS05015 | N-acetyltransferase | 2.210264461 | 0.02391967 |
| 53 | NJAUSS_RS06490 | ISL3 family transposase | 2.191664749 | 0.031043805 |
| 54 | NJAUSS_RS03570 | NAD(P)H-dependent oxidoreductase | 2.17107684 | 0.010726151 |
| 55 | NJAUSS_RS08860 | trimeric intracellular cation channel family protein | 2.168286089 | 0.029990342 |
| 56 | NJAUSS_RS07090 | serine/threonine transporter SstT | 2.165538169 | 0.027067329 |
| 57 | NJAUSS_RS06230 | sulfatase | 2.14536813 | 0.008353253 |
| 58 | NJAUSS_RS09550 | RluA family pseudouridine synthase | 2.137586566 | 0.003358871 |
| 59 | NJAUSS_RS02570 | histidine phosphatase family protein | 2.124942432 | 0.04033318 |
| 60 | NJAUSS_RS07020 | ribose-5-phosphate isomerase | 2.105243888 | 0.029540673 |
| 61 | NJAUSS_RS00440 | 50S ribosomal protein L4 | 2.093498754 | 0.002531248 |
| 62 | NJAUSS_RS01515 | multidrug ABC transporter permease/ATP-binding protein | 2.088716977 | 0.025367292 |
| 63 | NJAUSS_RS01340 | membrane protein | 2.079616925 | 0.025374717 |
| 64 | NJAUSS_RS07630 | hypothetical protein | 2.074205149 | 0.009880437 |
| 65 | NJAUSS_RS01370 | DUF1700 domain-containing protein | 2.065675353 | 0.011199816 |
| 66 | NJAUSS_RS05220 | cysteine desulfurase | 2.065061634 | 0.033841165 |
| 67 | NJAUSS_RS01615 | DUF1349 domain-containing protein | 2.021104411 | 0.012491804 |
| 68 | NJAUSS_RS09860 | L-ascorbate 6-phosphate lactonase | 2.010490666 | 0.020879 |
| 69 | NJAUSS_RS02720 | amino acid ABC transporter permease | 2.008136278 | 0.025423627 |
| 70 | NJAUSS_RS04195 | permease | 2.00299632 | 0.009627936 |
| 71 | NJAUSS_RS01365 | hypothetical protein | 1.99487237 | 0.019768574 |
| 72 | NJAUSS_RS08835 | hypothetical protein | 1.983297641 | 0.011197286 |
| 73 | NJAUSS_RS06195 | DUF2142 domain-containing protein | 1.976879041 | 0.012152209 |
| 74 | NJAUSS_RS08185 | acyltransferase | 1.973861396 | 0.005874947 |
| 75 | NJAUSS_RS06155 | hypothetical protein | 1.96623605 | 0.02985158 |
| 76 | NJAUSS_RS01385 | LrgB family protein | 1.944046696 | 0.031620934 |
| 77 | NJAUSS_RS07045 | tRNA uridine-5-carboxymethylaminomethyl(34) synthesis GTPase MnmE | 1.928219409 | 0.00751348 |
| 78 | NJAUSS_RS02545 | ABC transporter ATP-binding protein | 1.927783343 | 0.011584215 |
| 79 | NJAUSS_RS08235 | CBS domain-containing protein | 1.892491214 | 0.013670388 |
| 80 | NJAUSS_RS05405 | ion channel protein | 1.866157047 | 0.044552461 |
| 81 | NJAUSS_RS05270 | ABC transporter permease | 1.825248699 | 0.014672275 |
| 82 | NJAUSS_RS03680 | arginine regulator | 1.818573542 | 0.018946978 |
| 83 | NJAUSS_RS00935 | ribonuclease J | 1.813199787 | 0.008930168 |
| 84 | NJAUSS_RS04015 | sugar-binding transcriptional regulator | 1.800285821 | 0.048429242 |
| 85 | NJAUSS_RS09410 | aquaporin family protein | 1.793445713 | 0.013735826 |
| 86 | NJAUSS_RS06495 | peptide chain release factor 3 | 1.783090391 | 0.010979244 |
| 87 | NJAUSS_RS05480 | peptidase T | 1.734655607 | 0.011519592 |
| 88 | NJAUSS_RS04900 | hemolysin III | 1.731683077 | 0.025259972 |
| 89 | NJAUSS_RS10870 | hypothetical protein | 1.726445415 | 0.044575355 |
| 90 | NJAUSS_RS05175 | ABC transporter ATP-binding protein | 1.699943067 | 0.025220697 |
| 91 | NJAUSS_RS00890 | 30S ribosomal protein S12 | 1.694455273 | 0.024668426 |
| 92 | NJAUSS_RS04375 | hypothetical protein | 1.689972582 | 0.03843645 |
| 93 | NJAUSS_RS07940 | hydroxymethylglutaryl-CoA synthase | 1.641579298 | 0.030287951 |
| 94 | NJAUSS_RS07030 | type I restriction endonuclease subunit S | 1.64077178 | 0.043781582 |
| 95 | NJAUSS_RS01390 | formate transporter | 1.626042464 | 0.030249615 |
| 96 | NJAUSS_RS10670 | IS630 family transposase | 1.625750354 | 0.027120659 |
| 97 | NJAUSS_RS10620 | IS630 family transposase | 1.625747909 | 0.02710673 |
| 98 | NJAUSS_RS10855 | IS630 family transposase | 1.625744835 | 0.027089229 |
| 99 | NJAUSS_RS10750 | IS630 family transposase | 1.625634768 | 0.026468467 |
| 100 | NJAUSS_RS04430 | ABC transporter ATP-binding protein | 1.622677805 | 0.049527406 |
| 101 | NJAUSS_RS07905 | dehydrogenase | 1.611695349 | 0.030857077 |
| 102 | NJAUSS_RS04875 | sugar O-acetyltransferase | 1.608541957 | 0.046074212 |
| 103 | NJAUSS_RS06115 | LTA synthase family protein | 1.595850783 | 0.028866768 |
| 104 | NJAUSS_RS08265 | aminopeptidase C | 1.588924104 | 0.023426383 |
| 105 | NJAUSS_RS08850 | alpha-ketoacid dehydrogenase subunit beta | 1.583026182 | 0.024671099 |
| 106 | NJAUSS_RS05185 | phosphate acetyltransferase | 1.581592709 | 0.033827455 |
| 107 | NJAUSS_RS08460 | 1,4-dihydroxy-2-naphthoate polyprenyltransferase | 1.545377865 | 0.046176523 |
| 108 | NJAUSS_RS02385 | cell division protein FtsA | 1.541029411 | 0.04275048 |
| 109 | NJAUSS_RS07830 | type I methionyl aminopeptidase | 1.536667438 | 0.042736089 |
| 110 | NJAUSS_RS09545 | ferredoxin--NADP(+) reductase | 1.51770602 | 0.030381829 |
| 111 | NJAUSS_RS10470 | ABC-F family ATPase | 1.507962283 | 0.032334924 |
| 112 | NJAUSS_RS05160 | XRE family transcriptional regulator | 1.49905184 | 0.046787994 |
| 113 | NJAUSS_RS07005 | purine-nucleoside phosphorylase | 1.496437799 | 0.030109829 |
| 114 | NJAUSS_RS02665 | hypothetical protein | 1.475541732 | 0.033091367 |
| 115 | NJAUSS_RS10615 | IS5/IS1182 family transposase | 1.457487476 | 0.048529559 |
| 116 | NJAUSS_RS06440 | DNA translocase FtsK | 1.427201381 | 0.04804579 |
| 117 | NJAUSS_RS08390 | ATP-dependent helicase | -1.407674386 | 0.04805374 |
| 118 | NJAUSS_RS04530 | 1-phosphofructokinase | -1.46909459 | 0.046351913 |
| 119 | NJAUSS_RS07965 | HU family DNA-binding protein | -1.478032944 | 0.032695624 |
| 120 | NJAUSS_RS03645 | arginine deiminase | -1.516456917 | 0.043294176 |
| 121 | NJAUSS_RS02855 | N-acetylneuraminate synthase | -1.524669333 | 0.0426627 |
| 122 | NJAUSS_RS09160 | ABC transporter ATP-binding protein | -1.628172589 | 0.031338364 |
| 123 | NJAUSS_RS02630 | triose-phosphate isomerase | -1.689320871 | 0.015669288 |
| 124 | NJAUSS_RS05810 | phosphocarrier protein HPr | -1.711727107 | 0.036638817 |
| 125 | NJAUSS_RS08065 | pyruvate formate lyase-activating protein | -1.74181947 | 0.044807516 |
| 126 | NJAUSS_RS05355 | phosphate transport system regulatory protein PhoU | -1.781050358 | 0.027591993 |
| 127 | NJAUSS_RS05295 | 2-deoxyribose-5-phosphate aldolase | -1.814056075 | 0.019120953 |
| 128 | NJAUSS_RS06385 | 2-dehydropantoate 2-reductase | -1.856987906 | 0.031728983 |
| 129 | NJAUSS_RS04960 | orotidine-5'-phosphate decarboxylase | -1.885811152 | 0.029255595 |
| 130 | NJAUSS_RS08780 | elongation factor P | -1.901257592 | 0.014326357 |
| 131 | NJAUSS_RS04855 | 50S ribosomal protein L7/L12 | -1.903962404 | 0.019344514 |
| 132 | NJAUSS_RS05390 | inositol monophosphatase family protein | -1.915768292 | 0.045564154 |
| 133 | NJAUSS_RS07120 | phosphoglucosamine mutase | -1.916101083 | 0.010161581 |
| 134 | NJAUSS_RS00575 | DNA-directed RNA polymerase subunit alpha | -1.921611892 | 0.006212106 |
| 135 | NJAUSS_RS07455 | superoxide dismutase | -1.924784516 | 0.011181532 |
| 136 | NJAUSS_RS02060 | guanylate kinase | -1.948082586 | 0.038437813 |
| 137 | NJAUSS_RS06300 | adenine phosphoribosyltransferase | -1.991297447 | 0.016209467 |
| 138 | NJAUSS_RS05770 | chromosome segregation protein SMC | -2.003900081 | 0.012094347 |
| 139 | NJAUSS_RS04950 | orotate phosphoribosyltransferase | -2.037058935 | 0.035340933 |
| 140 | NJAUSS_RS04525 | DeoR/GlpR transcriptional regulator | -2.077257386 | 0.006951091 |
| 141 | NJAUSS_RS01140 | ROK family protein | -2.094103795 | 0.039665145 |
| 142 | NJAUSS_RS04940 | uracil-DNA glycosylase | -2.099345796 | 0.03788282 |
| 143 | NJAUSS_RS09280 | N-acetylmuramic acid 6-phosphate etherase | -2.249197403 | 0.010251058 |
| 144 | NJAUSS_RS04470 | spermidine/putrescine ABC transporter substrate-binding protein | -2.291421317 | 0.003796709 |
| 145 | NJAUSS_RS08715 | HAD family phosphatase | -2.294600738 | 0.00162913 |
| 146 | NJAUSS_RS06390 | S1 RNA-binding protein | -2.340680806 | 0.007387961 |
| 147 | NJAUSS_RS08820 | 30S ribosomal protein S6 | -2.443755594 | 0.002858787 |
| 148 | NJAUSS_RS00540 | 50S ribosomal protein L15 | -2.647783073 | 0.015204748 |
| 149 | rss14 | sRNA detected from our previous work [[4](#_ENREF_4)] | -2.738021646 | 0.006969615 |
| 150 | NJAUSS_RS03325 | DUF2800 domain-containing protein | -2.793946012 | 0.046872311 |
| 151 | NJAUSS_RS04860 | 50S ribosomal protein L10 | -2.970836309 | 0.000303464 |
| 152 | NJAUSS_RS01445 | alcohol dehydrogenase AdhP | -3.045009705 | 0.026745372 |
| 153 | NJAUSS_RS08815 | single-stranded DNA-binding protein | -3.093292833 | 6.14E-05 |
| 154 | rss16(Rnase_P) | sRNA detected from our previous work [[4](#_ENREF_4)] | -4.494038038 | 3.02E-05 |
| 155 | NJAUSS_RS00555 | translation initiation factor IF-1 | -5.049240091 | 0.001925474 |

* The order of the candidate targets is based on Log_2_ fold change (WT-pSET2-MS2-rss03/Δrss03-pSET2-MS2-negative). The red color indicates the direct targets identified by gel retardation assays. The green color indicates the indirect targets identified by gel retardation assays.

# References

4. Wu ZF, Wu CY, Shao J, et al. The Streptococcus suis transcriptional landscape reveals adaptation mechanisms in pig blood and cerebrospinal fluid. Rna. 2014 Jun;20(6):882-898.
