# Supplementary material for: A small RNA from Streptococcus suis epidemic ST7 strain promotes bacterial survival in host blood and brain by enhancing oxidative stress resistance
Source: Virulence. 2025 Apr 16;16(1):2491635. doi: 10.1080/21505594.2025.2491635 (PMC12005413; doi:10.1080/21505594.2025.2491635)
Supplement: Table S8.docx [file KVIR_A_2491635_SM4074.docx]

# Table S8. The information of the different strains used for GlpF homologs analysis.

| **Number** | **Genbank accession** | **Strain** | **Species** | **Gene_ID** | **Coverage (%)** | **Homologs (%)** |
| --- | --- | --- | --- | --- | --- | --- |
| 1 | NC_020526.1 | SC070731 | *Streptococcus suis* | NJAUSS_RS09410 | 100 | 100 |
| 2 | NC_020526.1 | SC070731 | *Streptococcus suis* | NJAUSS_RS04085 | 92 | 58.45 |
| 3 | NC_020526.1 | SC070731 | *Streptococcus suis* | NJAUSS_RS01395 | 98 | 35.16 |
| 4 | NZ_CP017142.1 | GZ0565 | *Streptococcus suis* | BFP66_RS01250 | 98 | 34.07 |
| 5 | NC_018646.1 | GD201008-001 | *Streptococcus agalactiae* | A964_RS08020 | 99 | 71.06 |
| 6 | NC_018646.1 | GD201008-001 | *Streptococcus agalactiae* | A964_RS01710 | 98 | 53.88 |
| 7 | NC_018646.1 | GD201008-001 | *Streptococcus agalactiae* | A964_RS08390 | 98 | 36.26 |
| 8 | NC_008533.1 | SD39 | *Streptococcus pneumoniae* | SPD_2011 | 97 | 58.87 |
| 9 | NC_008533.1 | SD39 | *Streptococcus pneumoniae* | SPD_1320 | 87 | 34.77 |
| 10 | NC_008533.1 | SD39 | *Streptococcus pneumoniae* | SPD_1569 | 96 | 28.14 |
| 11 | NC_002516.2 | PAO1 | *Pseudomonas aeruginosa* | PA3581 | 98 | 33.33 |
| 12 | NC_021175.1 | AS 1.3089 | *Streptococcus oligofermentans* | 1872_01445 | 96 | 28.76 |
| 13 | NC_004567.2 | WCFS1 | *Lactiplantibacillus plantarum* | lp_0171 | 98 | 57.76 |
| 14 | NC_004567.2 | WCFS1 | *Lactiplantibacillus plantarum* | lp_0372 | 97 | 42.49 |
| 15 | NC_004567.2 | WCFS1 | *Lactiplantibacillus plantarum* | lp_3436 | 97 | 41.00 |
| 16 | NC_004567.2 | WCFS1 | *Lactiplantibacillus plantarum* | lp_3463 | 97 | 29.79 |
| 17 | NC_000913.3 | K-12 substr. MG1655 | *Escherichia coli* | b3927 | 98 | 34.14 |
| 18 | NC_000913.3 | K-12 substr. MG1655 | *Escherichia coli* | b0875 | 42 | 34.65 |
| 19 | NC_014408.1 | Marburg | *Methanothermobacter marburgensis* | MTBMA_RS02725 | 97 | 30.49 |
| 20 | / | / | *Homo sapiens* | 360 | 44 | 45.28 |
| 21 | / | / | *Homo sapiens* | 358 | 87 | 27.14 |
