# Supplementary material for: A small RNA from Streptococcus suis epidemic ST7 strain promotes bacterial survival in host blood and brain by enhancing oxidative stress resistance
Source: Virulence. 2025 Apr 16;16(1):2491635. doi: 10.1080/21505594.2025.2491635 (PMC12005413; doi:10.1080/21505594.2025.2491635)
Supplement: Table S4.docx [file KVIR_A_2491635_SM4072.docx]

# Table S4. The information of the different strains used for rss03 homologs analysis.

| **Number** | **Genbank accession** | **Strain** | **Species** | **Sequence types** | **Homologs** | **Serotype** | **Isolation sourse** |
| --- | --- | --- | --- | --- | --- | --- | --- |
| 1 | NZ_LR738722.1 | 861160 | *Streptococcus suis* | ST20 | 100% | SS2 | Patient |
| 2 | NC_017618.1 | JS14 | *Streptococcus suis* | ST7 | 100% | SS14 | Diseased pig |
| 3 | NZ_CP065431.1 | yp20190405 | *Streptococcus suis* | ST1 | 100% | SS2 | / |
| 4 | NZ_CP082198.1 | cNJ3 | *Streptococcus suis* | ST1 | 100% | SS2 | / |
| 5 | NZ_CP082199.1 | cFJSM5 | *Streptococcus suis* | ST1 | 100% | SS2 | / |
| 6 | NZ_CP082200.1 | cDY107 | *Streptococcus suis* | ST1 | 100% | SS2 | / |
| 7 | NZ_CP082201.1 | cAKJ18 | *Streptococcus suis* | ST1 | 100% | SS2 | / |
| 8 | NZ_CP091423.1 | LSM29 | *Streptococcus suis* | ST242 | 100% | SS2 | Patient |
| 9 | NZ_CP091422.1 | LSM157 | *Streptococcus suis* | ST665 | 100% | SS2 | Patient |
| 10 | NZ_CP102137.1 | M104300_S20 | *Streptococcus suis* | ST1 | 100% | SS2 | Diseased pig |
| 11 | NZ_CP102154.1 | SS15055_N2_C15 | *Streptococcus suis* | ST1 | 100% | SS2 | Healthy pig |
| 12 | NZ_CP095463.1 | TJS56 | *Streptococcus suis* | ST1 | 100% | SS2 | Healthy pig |
| 13 | NZ_LS483418.1 | NCTC10234 | *Streptococcus suis* | ST1 | 100% | SS2 | Diseased pig |
| 14 | NZ_CP082948.1 | SZ1908 | *Streptococcus suis* | ST7 | 100% | SS2 | Patient |
| 15 | NZ_CP030022.1 | ISU2714 | *Streptococcus suis* | ST1 | 100% | SS2 | Diseased pig |
| 16 | NZ_CP030017.1 | ISU1606 | *Streptococcus suis* | ST1 | 100% | SS2 | Diseased pig |
| 17 | NZ_CP024050.1 | CS100322 | *Streptococcus suis* | ST7 | 100% | SS2 | / |
| 18 | NC_012926.1 | BM407 | *Streptococcus suis* | ST1 | 100% | SS2 | Patient |
| 19 | NZ_LR738721.1 | S10 | *Streptococcus suis* | ST1 | 100% | SS2 | Diseased pig |
| 20 | CP000408.1 | 98HAH33 | *Streptococcus suis* | ST7 | 100% | SS2 | Patient |
| 21 | NZ_CP058742.1 | 10 | *Streptococcus suis* | ST1 | 100% | SS2 | Healthy pig |
| 22 | NC_018526.1 | S735 | *Streptococcus suis* | ST1 | 100% | SS2 | Diseased pig |
| 23 | NC_012925.1 | P1/7 | *Streptococcus suis* | ST1 | 100% | SS2 | Diseased pig |
| 24 | NC_017617.1 | GZ1 | *Streptococcus suis* | ST1 | 100% | SS2 | Patient |
| 25 | NC_017622.1 | A7 | *Streptococcus suis* | ST7 | 100% | SS2 | Diseased pig |
| 26 | NZ_CP018908.1 | SS2-1 | *Streptococcus suis* | ST7 | 100% | SS2 | Diseased pig |
| 27 | NZ_CP007497.1 | ZY05719 | *Streptococcus suis* | ST7 | 100% | SS2 | Diseased pig |
| 28 | NC_012924.1 | SC84 | *Streptococcus suis* | ST7 | 100% | SS2 | Patient |
| 29 | NZ_CP020863.1 | SC19 | *Streptococcus suis* | ST7 | 100% | SS2 | Diseased pig |
| 30 | CP000407.1 | 05ZYH33 | *Streptococcus suis* | ST945 | 100% | SS2 | Patient |
| 31 | NZ_CP047248.1 | LSM178 | *Streptococcus suis* | ST1005 | 100% | SS2 | Patient |
| 32 | NZ_CP016175.1 | LSM102 | *Streptococcus suis* | ST658 | 100% | SS2 | Patient |
| 33 | NC_020526.1 | SC070731 | *Streptococcus suis* | ST7 | 100% | SS2 | Diseased pig |
| 34 | NZ_CP085088.1 | Ssuis_MA1 | *Streptococcus suis* | ST1 | 100% | SS1 | Diseased pig |
| 35 | NZ_CP109941.1 | ID38828 | *Streptococcus suis* | ST105 | 100% | SS1 | Patient |
| 36 | NZ_CP102152.1 | DNS11 | *Streptococcus suis* | ST16 | ＜100% | SS9 | Diseased pig |
| 37 | NZ_LR738723.1 | GD-0088 | *Streptococcus suis* | ST16 | ＜100% | SS9 | Diseased pig |
| 38 | NZ_CP039462.1 | WUSS351 | *Streptococcus suis* | ST2618 | ＜100% | NCL4 | Healthy pig |
| 39 | NZ_CP100432.1 | T15 | *Streptococcus suis* | ST19 | ＜100% | SS2 | Healthy pig |
| 40 | NZ_CP030124.1 | SH1510 | *Streptococcus suis* | ST94 | ＜100% | SS4 | Diseased pig |
| 41 | NZ_CP058740.1 | 16085/3b | *Streptococcus suis* | ST94 | ＜100% | SS9 | Diseased pig |
| 42 | NZ_CP032064.1 | YSJ17 | *Streptococcus suis* | ST1071 | ＜100% | NCL1 | Pig |
| 43 | CP025095.1 | HN136 | *Streptococcus suis* | ST264 | ＜100% | CHz | Pig |
| 44 | NZ_CP025043.1 | AH681 | *Streptococcus suis* | ST475 | ＜100% | CHz | Pig |
| 45 | NZ_CP024974.1 | CZ130302 | *Streptococcus suis* | ST383 | ＜100% | CHz | Diseased pig |
| 46 | NZ_CP008921.1 | 6407 | *Streptococcus suis* | ST54 | ＜100% | SS4 | Diseased pig |
| 47 | NZ_CP017142.1 | GZ0565 | *Streptococcus suis* | ST243 | ＜100% | SS9 | Diseased pig |
| 48 | NZ_CP015557.1 | DN13 | *Streptococcus suis* | ST243 | ＜100% | SS9 | Diseased pig |
| 49 | NZ_LR738724.1 | 9401240 | *Streptococcus suis* | ST220 | ＜100% | SS9 | Diseased pig |
| 50 | NC_017950.1 | ST1 | *Streptococcus suis* | ST13 | ＜100% | SS1 | / |
| 51 | NZ_LR594043.1 | NCTC10237 | *Streptococcus suis* | ST13 | ＜100% | SS1 | Pig |
| 52 | NC_017621.1 | D12 | *Streptococcus suis* | ST619 | ＜100% | SS9 | / |
| 53 | NC_021213.1 | TL13 | *Streptococcus suis* | ST664 | ＜100% | SS16 | Healthy pig |
| 54 | NZ_CP030125.1 | HA1003 | *Streptococcus suis* | ST1006 | ＜100% | SS4 | Healthy pig |
| 55 | NZ_CP017667.1 | 1081 | *Streptococcus suis* | ST2619 | ＜100% | SS31 | Healthy pig |
| 56 | NZ_CP017666.1 | 61 | *Streptococcus suis* | ST2619 | ＜100% | SS31 | Healthy pig |
| 57 | NZ_CP079193.1 | ID41570 | *Streptococcus suis* | ST221 | ＜100% | SS5 | Patient |
| 58 | NZ_CP024126.1 | HA0609 | *Streptococcus suis* | ST28 | ＜100% | SS1/2 | Pig |
| 59 | NZ_CP025419.1 | SH0104 | *Streptococcus suis* | ST28 | ＜100% | SS1/2 | Pig |
| 60 | NZ_CP102140.1 | DNC49 | *Streptococcus suis* | ST28 | ＜100% | SS1/2 | Healthy pig |
| 61 | NZ_CP031377.1 | ISU2614 | *Streptococcus suis* | ST28 | ＜100% | SS2 | Diseased pig |
| 62 | NZ_CP030020.1 | ISU2514 | *Streptococcus suis* | ST25 | ＜100% | SS2 | Diseased pig |
| 63 | NZ_CP031379.1 | ISU2660 | *Streptococcus suis* | ST787 | ＜100% | SS2 | Diseased pig |
| 64 | NZ_CP030023.1 | ISU2414 | *Streptococcus suis* | ST620 | ＜100% | SS2 | Diseased pig |
| 65 | NZ_CP002007.2 | 05HAS68 | *Streptococcus suis* | ST28 | ＜100% | SS2 | Healthy pig |
| 66 | NZ_CP012731.1 | 90-1330 | *Streptococcus suis* | ST28 | ＜100% | SS2 | Diseased pig |
| 67 | NZ_CP012911.1 | NSUI060 | *Streptococcus suis* | ST25 | ＜100% | SS2 | Diseased pig |
| 68 | NZ_CP011419.1 | NSUI002 | *Streptococcus suis* | ST28 | ＜100% | SS2 | Pig |
| 69 | NZ_CP095182.1 | 89-1591 | *Streptococcus suis* | ST25 | ＜100% | SS2 | Diseased pig |
| 70 | NZ_CP102143.1 | DNR43 | *Streptococcus suis* | ST28 | ＜100% | SS2 | Diseased pig |
| 71 | NZ_CP095162.1 | TJS75 | *Streptococcus suis* | ST25 | ＜100% | SS2 | Pig |
| 72 | CP002633.1 | ST3 | *Streptococcus suis* | ST35 | ＜100% | SS3 | / |
| 73 | NZ_CP078543.1 | YB51 | *Streptococcus suis* | ST105 | ＜100% | SS3 | / |
| 74 | NZ_CP041994.1 | INT-01 | *Streptococcus suis* | ST35 | ＜100% | SS3 | Diseased pig |
| 75 | NZ_CP058741.1 | 13-00283-02 | *Streptococcus suis* | ST29 | ＜100% | SS7 | Diseased pig |
| 76 | NZ_CP017092.1 | ISU2812 | *Streptococcus suis* | ST76 | ＜100% | / | Pig |
| 77 | NZ_CP082205.1 | AKJ18 | *Streptococcus suis* | ST2832 | ＜100% | / | Healthy pig |
| 78 | NZ_CP065430.1 | YZDH1 | *Streptococcus suis* | ST2833 | ＜100% | / | Pig |
| 79 | NZ_CP116393.1 | SS/UPM/MY/F001 | *Streptococcus suis* | ST2105 | ＜100% | / | Cat |
| 80 | NZ_CP140109.1 | 2022WUSS148 | *Streptococcus suis* | ST1942 | ＜100% | / | Diseased pig |
| 81 | NZ_CP135087.1 | ID26102 | *Streptococcus suis* | ST236 | ＜100% | / | Healthy pig |
| 82 | NZ_CP071697.1 | 1112S | *Streptococcus suis* | ST1615 | ＜100% | / | Diseased pig |
| 83 | NZ_CP102136.1 | M105052_S26 | *Streptococcus suis* | ST2834 | ＜100% | / | Diseased pig |
| 84 | NZ_CP134473.1 | TMW_SS028 | *Streptococcus suis* | ST792 | ＜100% | / | Pig |
| 85 | NZ_CP071806.1 | GX69 | *Streptococcus suis* | ST373 | ＜100% | / | Patient |
| 86 | NZ_CP135090.1 | NZ_CP135090.1 | *Streptococcus suis* | ST236 | ＜100% | / | Patient |
| 87 | NZ_CP134488.1 | NLS50 | *Streptococcus suis* | ST16 | ＜100% | / | Diseased pig |
| 88 | NZ_CP071305.1 | SC183 | *Streptococcus suis* | ST316 | ＜100% | / | Pig |
| 89 | NZ_CP141824.1 | HB18 | *Streptococcus suis* | ST850 | ＜100% | / | Pig |
| 90 | NZ_AP023392.1 | DAT300 | *Streptococcus suis* | ST115 | ＜100% | / | Healthy pig |
| 91 | NZ_CP100431.1 | 1521251 | *Streptococcus suis* | ST1175 | ＜100% | / | Pig |
| 92 | NZ_CP102141.1 | DNR48 | *Streptococcus suis* | ST1770 | ＜100% | / | Diseased pig |
| 93 | NZ_CP109937.1 | TRG6 | *Streptococcus suis* | ST94 | ＜100% | / | Diseased pig |
| 94 | NZ_CP109940.1 | ID34572 | *Streptococcus suis* | ST1689 | ＜100% | / | Healthy pig |
| 95 | NZ_CP082202.1 | SS389 | *Streptococcus suis* | ST2835 | ＜100% | / | Pig |
| 96 | NZ_CP100334.1 | STC83 | *Streptococcus suis* | ST1656 | ＜100% | SS2 | Patient |
| 97 | NZ_CP100418.1 | STC104 | *Streptococcus suis* | ST1656 | ＜100% | SS2 | Patient |
| 98 | NZ_CP100340.1 | STC78 | *Streptococcus suis* | ST1656 | ＜100% | SS2 | Patient |
| 99 | NZ_CP100338.1 | STC80 | *Streptococcus suis* | ST1656 | ＜100% | SS2 | Patient |
| 100 | NZ_CP100326.1 | STC90 | *Streptococcus suis* | ST1656 | ＜100% | SS2 | Patient |
| 101 | NZ_CP100332.1 | STC84 | *Streptococcus suis* | ST1656 | ＜100% | SS2 | Patient |
| 102 | NZ_CP100336.1 | STC81 | *Streptococcus suis* | ST1656 | ＜100% | SS2 | Patient |
| 103 | NZ_CP100330.1 | STC85 | *Streptococcus suis* | ST1656 | ＜100% | SS2 | Patient |
| 104 | NZ_CP100328.1 | STC86 | *Streptococcus suis* | ST1656 | ＜100% | SS2 | Patient |
| 105 | NZ_CP101844.1 | 2018WUSS151 | *Streptococcus suis* | ST1241 | ＜100% | / | Diseased pig |
| 106 | NZ_CP102135.1 | M106471_S40 | *Streptococcus suis* | ST2836 | ＜100% | / | Diseased pig |
| 107 | NZ_CP110141.1 | WUSS030 | *Streptococcus suis* | ST25 | ＜100% | / | Healthy pig |
| 108 | NZ_CP135093.1 | 1652329 | *Streptococcus suis* | ST483 | ＜100% | / | Patient |
| 109 | NZ_CP109939.1 | ID36054 | *Streptococcus suis* | ST94 | ＜100% | / | Patient |
| 110 | NZ_CP102148.1 | DNC15 | *Streptococcus suis* | ST2837 | ＜100% | / | Pig |
| 111 | NZ_CP134472.1 | 1522228 | *Streptococcus suis* | ST483 | ＜100% | / | Diseased pig |
| 112 | NZ_CP102094.1 | 12RC1 | *Streptococcus suis* | ST2838 | ＜100% | / | Pig |
| 113 | NZ_CP029398.1 | HN105 | *Streptococcus suis* | ST498 | ＜100% | / | Diseased pig |
| 114 | NZ_CP082203.1 | NJ3 | *Streptococcus suis* | ST240 | ＜100% | / | Diseased pig |
| 115 | NZ_CP135094.1 | 1547095 | *Streptococcus suis* | ST1197 | ＜100% | / | Patient |
| 116 | NZ_CP100430.1 | LSS42 | *Streptococcus suis* | ST878 | ＜100% | / | Pig |
| 117 | NZ_CP076517.1 | 39565 | *Streptococcus suis* | ST221 | ＜100% | / | Patient |
| 118 | NZ_CP149804.1 | YA | *Streptococcus suis* | ST1801 | ＜100% | / | Diseased pig |
| 119 | NZ_CP085085.1 | Ssuis_MA8 | *Streptococcus suis* | ST28 | ＜100% | / | Healthy pig |
| 120 | NZ_CP102145.1 | DNS20 | *Streptococcus suis* | ST2839 | ＜100% | / | Diseased pig |
| 121 | NZ_OX352806.1 | 861160_dxerD_hsdSA | *Streptococcus suis* | ST20 | ＜100% | SS2 | / |
| 122 | NZ_OX352831.1 | 861160_dxerD_hsdSE | *Streptococcus suis* | ST20 | ＜100% | SS2 | / |
| 123 | NZ_OX352941.1 | 861160_WT | *Streptococcus suis* | ST20 | ＜100% | SS2 | / |
| 124 | NZ_OX352944.1 | 861160_LM_H | *Streptococcus suis* | ST20 | ＜100% | SS2 | / |
| 125 | NZ_OX352940.1 | 861160_LM_A | *Streptococcus suis* | ST20 | ＜100% | SS2 | / |
| 126 | NZ_OX352996.1 | 861160_dhsdS | *Streptococcus suis* | ST20 | ＜100% | SS2 | / |
| 127 | NZ_CP068708.1 | ID33329 | *Streptococcus suis* | ST221 | ＜100% | / | Patient |
| 128 | NZ_CP082778.1 | ID32098 | *Streptococcus suis* | ST234 | ＜100% | / | Patient |
| 129 | NZ_AP023391.1 | DAT299 | *Streptococcus suis* | ST114 | ＜100% | / | Healthy pig |
| 130 | NZ_CP085087.1 | Ssuis_MA2 | *Streptococcus suis* | ST17 | ＜100% | / | Healthy pig |
| 131 | NZ_CP102138.1 | NZ_CP102138.1 | *Streptococcus suis* | ST123 | ＜100% | / | Diseased pig |
| 132 | NZ_LR738720.1 | GD-0001 | *Streptococcus suis* | ST20 | ＜100% | SS2 | Diseased pig |
| 133 | NZ_CP135089.1 | ID34567 | *Streptococcus suis* | ST235 | ＜100% | / | Healthy pig |
| 134 | NZ_CP109942.1 | ID35541 | *Streptococcus suis* | ST237 | ＜100% | / | Healthy pig |
| 135 | NZ_CP135063.1 | ID32563 | *Streptococcus suis* | ST235 | ＜100% | / | Patient |
| 136 | NZ_CP085086.1 | Ssuis_MA6 | *Streptococcus suis* | ST17 | ＜100% | / | Diseased pig |
| 137 | NZ_CP017088.1 | SRD478 | *Streptococcus suis* | ST785 | ＜100% | / | Healthy pig |
| 138 | NZ_CP135065.1 | ID24665 | *Streptococcus suis* | / | ＜100% | / | Patient |
| 139 | NZ_CP134477.1 | NLS40 | *Streptococcus suis* | ST13 | ＜100% | / | Diseased pig |
| 140 | CP017785.1 | ISU2912 | *Streptococcus suis* | ST1941 | ＜100% | / | Diseased pig |
| 141 | CP084908.1 | Ssuis_MA4 | *Streptococcus suis* | / | ＜100% | / | Healthy pig |
| 142 | CP082204.1 | FJSM5 | *Streptococcus suis* | ST1593 | ＜100% | / | Diseased pig |
| 143 | LT671674.1 | LS9N | *Streptococcus suis* | ST890 | ＜100% | / | / |
| 144 | NZ_CP102149.1 | DNC13 | *Streptococcus suis* | ST2840 | ＜100% | / | Healthy pig |
| 145 | NZ_CP097577.1 | 3112 | *Streptococcus suis* | ST2302 | ＜100% | / | Diseased fish |
| 146 | NZ_CP031970.1 | SFJ44 | *Streptococcus suis* | ST1087 | ＜100% | / | Healthy pig |
| 147 | NZ_CP139878.1 | Ss_46 | *Streptococcus suis* | ST17 | ＜100% | / | Diseased pig |
| 148 | NZ_CP139882.1 | Ss_08 | *Streptococcus suis* | ST24 | ＜100% | / | Diseased pig |
| 149 | NZ_CP139877.1 | Ss_48 | *Streptococcus suis* | ST2841 | ＜100% | / | Diseased pig |
| 150 | NZ_CP139876.1 | Ss_109 | *Streptococcus suis* | ST16 | ＜100% | / | Diseased pig |
| 151 | NZ_CP139163.1 | cnzyss2-311 | *Streptococcus suis* | ST1 | 100% | SS2 | Pig |
| 152 | NZ_CP152119.1 | Ss2301 | *Streptococcus suis* | ST7 | 100% | / | Human |
| 153 | NZ_CP102748.1 | cSFJ45 | *Streptococcus suis* | ST1 | 100% | SS2 | / |
| 154 | NC_017619.1 | SS12 | *Streptococcus suis* | ST1 | 100% | SS2 | / |
| 155 | NZ_CP139875.1 | Ss_134 | *Streptococcus suis* | ST1642 | 100% | / | Diseased pig |
| 156 | NZ_CP141904.1 | 8324 | *Streptococcus suis* | ST1 | 100% | SS2 | Human |
| 157 | NZ_CP102746.1 | cAKJ47-2 | *Streptococcus suis* | ST1 | 100% | SS2 | / |
| 158 | NZ_CP139879.1 | Ss_45 | *Streptococcus suis* | ST3 | 100% | SS2 | Diseased pig |
| 159 | NZ_CP139881.1 | Ss_21 | *Streptococcus suis* | ST1 | 100% | SS2 | Diseased pig |
| 160 | NZ_CP139880.1 | Ss_22 | *Streptococcus suis* | ST1 | 100% | / | Diseased pig |
| 161 | NZ_CP134469.1 | M104170_C2 | *Streptococcus suis* | ST2842 | ＜100% | / | Healthy pig |
| 162 | NZ_CP134474.1 | PH2016-081 | *Streptococcus suis* | ST15 | ＜100% | / | Diseased pig |
| 163 | CP134491.1 | 1628469 | *Streptococcus parasuis* | / | ＜100% | / | Pig |
| 164 | NZ_AP024276.1 | SUT-286 | *Streptococcus parasuis* | / | ＜100% | / | Healthy pig |
| 165 | NZ_CP128410.1 | 7500 | *Streptococcus parasuis* | / | ＜100% | / | Patient |
| 166 | NZ_CP102747.1 | SFJ45 | *Streptococcus parasuis* | / | ＜100% | / | Healthy pig |
| 167 | NZ_CP090522.1 | SS17 | *Streptococcus parasuis* | / | ＜100% | / | Pig |
| 168 | NZ_CP086728.1 | SS20 | *Streptococcus parasuis* | / | ＜100% | / | Pig |
| 169 | NZ_CP073632.1 | NN1 | *Streptococcus parasuis* | / | ＜100% | / | Patient |
| 170 | AP024280.1 | SUT-503 | *Streptococcus parasuis* | / | ＜100% | / | Healthy pig |
| 171 | AP024277.1 | SUT-380 | *Streptococcus parasuis* | / | ＜100% | / | Healthy pig |
| 172 | NZ_CP069079.1 | BS26 | *Streptococcus parasuis* | / | ＜100% | / | Patient |
| 173 | NZ_CP076721.1 | H35 | *Streptococcus parasuis* | / | ＜100% | / | Pig |
| 174 | NZ_CP137602.1 | 221006 | *Streptococcus parasuis* | / | ＜100% | / | Patient |
| 175 | NZ_AP025332.1 | GUT-184 | *Streptococcus ruminantium* | / | ＜100% | / | Diseased cattle |
| 176 | NZ_AP018400.1 | GUT-187 | *Streptococcus ruminantium* | / | ＜100% | / | / |
| 177 | NZ_AP025333.1 | GUT-189 | *Streptococcus ruminantium* | / | ＜100% | / | Diseased cattle |
| 178 | NZ_AP025331.1 | GUT-183 | *Streptococcus ruminantium* | / | ＜100% | / | Diseased cattle |
