# Supplementary material for: A small RNA from Streptococcus suis epidemic ST7 strain promotes bacterial survival in host blood and brain by enhancing oxidative stress resistance
Source: Virulence. 2025 Apr 16;16(1):2491635. doi: 10.1080/21505594.2025.2491635 (PMC12005413; doi:10.1080/21505594.2025.2491635)
Supplement: Table S7.docx [file KVIR_A_2491635_SM4071.docx]

# Table S7. The candidate targets obtained by proteome.

| **Number** | **Gene_ID** | **Annotation** | **Fold change*** | ***p*-value** |
| --- | --- | --- | --- | --- |
| 1 | NJAUSS_RS00380 | DNA-3-methyladenine glycosylase I | + | 0 |
| 2 | NJAUSS_RS03305 | ImmA/IrrE family metallo-endopeptidase | + | 0 |
| 3 | NJAUSS_RS05625 | mannonate dehydratase | + | 0 |
| 4 | NJAUSS_RS07870 | bifunctional biotin--[acetyl-CoA-carboxylase] synthetase/biotin operon repressor | + | 0 |
| 5 | NJAUSS_RS09285 | MurR family transcriptional regulator | + | 0 |
| 6 | NJAUSS_RS09450 | deoxyribonuclease | + | 0 |
| 7 | NJAUSS_RS00190 | rod shape-determining protein MreD | - | 0 |
| 8 | NJAUSS_RS01445 | alcohol dehydrogenase AdhP | - | 0 |
| 9 | NJAUSS_RS03075 | tetracycline resistance ribosomal protection protein Tet(O) | - | 0 |
| 10 | NJAUSS_RS07915 | peptidoglycan GlcNAc deacetylase | - | 0 |
| 11 | NJAUSS_RS08065 | pyruvate formate lyase-activating protein | - | 0 |
| 12 | NJAUSS_RS09005 | cysteine desulfurase | - | 0 |
| 13 | NJAUSS_RS09010 | Fe-S cluster assembly protein SufD | - | 0 |
| 14 | NJAUSS_RS09410 | aquaporin family protein | - | 0 |
| 15 | NJAUSS_RS10015 | hypothetical protein | - | 0 |
| 16 | NJAUSS_RS04340 | 50S ribosomal protein L21 | 0.614394 | 6.598E-05 |
| 17 | NJAUSS_RS06235 | glucosaminidase | 3.041999 | 0.0004722 |
| 18 | NJAUSS_RS08995 | Fe-S cluster assembly protein SufB | 0.493184 | 0.0005456 |
| 19 | NJAUSS_RS08650 | acetyl-CoA carboxylase carboxyl transferase subunit alpha | 1.706863 | 0.0009342 |
| 20 | NJAUSS_RS01035 | DUF536 domain-containing protein | 1.529222 | 0.0010621 |
| 21 | NJAUSS_RS05020 | peptidase M26 | 1.516495 | 0.0013405 |
| 22 | NJAUSS_RS08655 | acetyl-CoA carboxylase carboxyl transferase subunit beta | 1.57629 | 0.0014449 |
| 23 | NJAUSS_RS04010 | DeoR/GlpR transcriptional regulator | 2.100967 | 0.0023272 |
| 24 | NJAUSS_RS08770 | transcription antitermination factor NusB | 1.712859 | 0.0029767 |
| 25 | NJAUSS_RS06240 | cell wall hydrolase | 2.41566 | 0.0052308 |
| 26 | NJAUSS_RS05115 | galactose-6-phosphate isomerase subunit LacB | 1.771222 | 0.0052673 |
| 27 | NJAUSS_RS08730 | alanine racemase | 1.520704 | 0.0066288 |
| 28 | NJAUSS_RS01765 | universal stress protein | 2.154513 | 0.0069244 |
| 29 | NJAUSS_RS00960 | AraC family transcriptional regulator | 1.780563 | 0.0077891 |
| 30 | NJAUSS_RS08695 | acyl carrier protein | 1.791681 | 0.0078732 |
| 31 | NJAUSS_RS01890 | hypothetical protein | 1.622545 | 0.0084609 |
| 32 | NJAUSS_RS07610 | ATP-dependent helicase | 0.547869 | 0.0110489 |
| 33 | NJAUSS_RS08355 | transcriptional regulator NrdR | 1.583971 | 0.0114277 |
| 34 | NJAUSS_RS06365 | 5-methyltetrahydropteroyltriglutamate-homocysteine methyltransferase | 1.833273 | 0.0115538 |
| 35 | NJAUSS_RS03175 | toxin PezT | 1.688608 | 0.0133101 |
| 36 | NJAUSS_RS00885 | chaperonin GroEL | 0.65112 | 0.0139991 |
| 37 | NJAUSS_RS01245 | hypothetical protein | 1.512082 | 0.0187631 |
| 38 | NJAUSS_RS06055 | ABC transporter ATP-binding protein | 1.710528 | 0.0247724 |
| 39 | NJAUSS_RS10240 | sugar ABC transporter permease | 0.654051 | 0.0254212 |
| 40 | NJAUSS_RS01855 | ribosome assembly RNA-binding protein YhbY | 1.732482 | 0.025806 |
| 41 | NJAUSS_RS09835 | serine/threonine protein phosphatase | 1.862111 | 0.0265254 |
| 42 | NJAUSS_RS04015 | sugar-binding transcriptional regulator | 5.596805 | 0.0272231 |
| 43 | NJAUSS_RS01610 | TIGR01440 family protein | 1.530882 | 0.0290622 |
| 44 | NJAUSS_RS04200 | ABC transporter ATP-binding protein | 1.800863 | 0.0292798 |
| 45 | NJAUSS_RS01565 | ABC transporter substrate-binding protein | 0.661098 | 0.0310281 |
| 46 | NJAUSS_RS06125 | 50S ribosomal protein L20 | 0.597083 | 0.0311995 |
| 47 | NJAUSS_RS09000 | SUF system NifU family Fe-S cluster assembly protein | 0.439436 | 0.0324037 |
| 48 | NJAUSS_RS04735 | DNA-binding protein | 1.607378 | 0.0390035 |
| 49 | NJAUSS_RS00230 | phosphoribosylformylglycinamidine synthase | 0.383316 | 0.0412765 |
| 50 | NJAUSS_RS00500 | type Z 30S ribosomal protein S14 | 0.380989 | 0.0422216 |
| 51 | NJAUSS_RS10125 | nucleotide pyrophosphohydrolase | 1.986744 | 0.0437098 |
| 52 | NJAUSS_RS08270 | BAX inhibitor (BI)-1/YccA family protein | 0.626221 | 0.0456213 |
| 53 | NJAUSS_RS09265 | GNAT family acetyltransferase | 2.043295 | 0.046935 |
| 54 | NJAUSS_RS08145 | DNA-binding response regulator | 1.733346 | 0.0494929 |

* The order of the candidate targets is based on fold change (Δ*rss03*/WT). The red color indicates direct targets identified by gel retardation assays. The green color indicates indirect targets identified by gel retardation assays. “+” indicates that the protein is only expressed in Δ*rss03* strain. “-” indicates that the protein is only expressed in WT strain.
