# Supplementary material for: A small RNA from Streptococcus suis epidemic ST7 strain promotes bacterial survival in host blood and brain by enhancing oxidative stress resistance
Source: Virulence. 2025 Apr 16;16(1):2491635. doi: 10.1080/21505594.2025.2491635 (PMC12005413; doi:10.1080/21505594.2025.2491635)
Supplement: Table S2.docx [file KVIR_A_2491635_SM4069.docx]

# Table S2. Primers used in this study.

| **Primers** | **Sequence (5’-3’)** | **Comment** |
| --- | --- | --- |
| **Construction of deletion strains** | | |
| Δ*rss03*-A | GGTAAGCAAAATGAAGATTAACGAAT | Upstream of fusion fragment for Δ*rss03* mutant |
| Δ*rss03*-B | AAGGAGTTTTCAGCATTATCCGATTCTATTCTACCATAATCTCAAGGA |  |
| *sacB-spc*-F | GGATAATGCTGAAAACTCCTT | Sucrose sensitivity and spectinomycin resistance gene |
| *sacB-spc*-R | AATCTGATTACCAATTAGAATGAATAT |  |
| Δ*rss03*-C | ATATTCATTCTAATTGGTAATCAGATTGTATACTAAGTGAGGCTGGGC | Downstream of fusion fragment for Δ*rss03* mutant |
| Δ*rss03*-D | CGAAAACCTGAACCCATGCTT |  |
| Δ*rss03*-A | GGTAAGCAAAATGAAGATTAACGAAT | Upstream of fusion fragment for Δ*rss03* mutant |
| Δ*rss03*-B1 | GCCCAGCCTCACTTAGTATACGATTCTATTCTACCATAATCTCAAGGA |  |
| Δ*rss03*-C1 | GTATACTAAGTGAGGCTGGGC | Downstream of fusion fragment for Δ*rss03* mutant |
| Δ*rss03*-D | CGAAAACCTGAACCCATGCTT |  |
| Δ*rss03*-E | TAAGGGGAGGGTAAAGGTACA | Detection and sequencing of deletion of *rss03* gene |
| Δ*rss03*-F | TTGTTGAGCTGAAACAGTCTATC |  |
| Δ*rss03*-G | GCCGAGTAGTGGGGGGATTAA | Detection of deletion of *rss03* gene |
| Δ*rss03*-H | CCACCATAAAGGTGGGCAAGG |  |
| *sacB-spc*-G | CATACTATGGCAAAAGCACAT | Detection of *sacB-spc* cassette gene |
| *sacB-spc*-H | TGGTTTTAGTCCACTCTCAAC |  |
| Δ*glpF*-A | GCTGGACTGTCATTGACCTTAATCA | Upstream of fusion fragment for Δ*glpF* mutant |
| Δ*glpF*-B | AAGGAGTTTTCAGCATTATCCAATGACCTCCTATTTATTGATTGTCAGTTC |  |
| Δ*glpF*-C | ATATTCATTCTAATTGGTAATCAGATTTCATTTGAATTAACTTTGATACATCGTCAC | Downstream of fusion fragment for Δ*glpF* mutant |
| Δ*glpF*-D | TCAGGTCAATGATGTCAAGTTGAAA |  |
| Δ*glpF*-A | GCTGGACTGTCATTGACCTTAATCA | Upstream of fusion fragment for Δ*glpF* mutant |
| Δ*glpF*-B1 | AATGACCTCCTATTTATTGATTGTCAGTTC |  |
| Δ*glpF*-C1 | GAACTGACAATCAATAAATAGGAGGTCATTTCATTTGAATTAACTTTGATACATCGTCAC | Downstream of fusion fragment for Δ*glpF* mutant |
| Δ*glpF*-D | TCAGGTCAATGATGTCAAGTTGAAA |  |
| Δ*glpF*-E | GATGGTAGCATCGGTACCAGC | Detection and sequencing of deletion of *glpF* gene |
| Δ*glpF*-F | AGGTGATACTGTAGAAGTCCA |  |
| Δ*glpF*-G | ATCCTGCATCCTCTCTAAAACCAAG | Detection of deletion of *glpF* gene |
| Δ*glpF*-H | TTATAGAGGATAGCTCCTGCGACAC |  |
| Δ*rnc*-A | AATCTGTTCTGCCAACTGCTC | Upstream of fusion fragment for Δ*rnc* mutant |
| Δ*rnc*-B | AAGGAGTTTTCAGCATTATCCTGGTCGCTCTAAGAAATTAGCTG |  |
| Δ*rnc*-C | ATATTCATTCTAATTGGTAATCAGATTCTATATGCTCCTTTCTAACTTAAACGAAA | Downstream of fusion fragment for Δ*rnc* mutant |
| Δ*rnc*-D | GATCTGTGGTTAGGGTCCAGG |  |
| Δ*rnc*-A | AATCTGTTCTGCCAACTGCTC | Upstream of fusion fragment for Δ*rnc* mutant |
| Δ*rnc*-B1 | TTTCGTTTAAGTTAGAAAGGAGCATATAGTGGTCGCTCTAAGAAATTAGCTG |  |
| Δ*rnc*-C1 | CTATATGCTCCTTTCTAACTTAAACGAAA | Downstream of fusion fragment for Δ*rnc* mutant |
| Δ*rnc*-D | GATCTGTGGTTAGGGTCCAGG |  |
| Δ*rnc*-E | CTACCTTGATTTCTTTCTGTTTATTGG | Detection and sequencing of deletion of *rnc* gene |
| Δ*rnc*-F | TTTCAAATAACCTGCGGCAAT |  |
| Δ*rnc*-G | ATAGCAATCTCACCATTGACC | Detection of deletion of *rnc* gene |
| Δ*rnc*-H | CTCCAGTTGATGATTTCCAAA |  |
|  |  |  |
| **Construction of expression plasmids** | | |
| pSET2-*rss03*-F | GAGTCAgaattcAAGCAGGCAATATCTGTTATA | Fusion fragment for construction of pSET2-*rss03* |
| pSET2-*rss03*-R | GAGTCAggatccCTTTTTGCCCAGCCTCACTTAGTAT |  |
| pSET2-F | TAAGTTGGGTAACGCCAGGG | Detection of the fragments inserted into pSET2 |
| pSET2-R | ACACAACATACGAGCCGGAA |  |
| pET28a-*rnc*-F | GAGTCAggatccATGAAAGATTTACATGCAAAATTATTGGA | Fusion fragment for construction of pET28a-*RS05775* |
| pET28a-*rnc*-R | CCCaagcttCTATCCTCTAGCCTCCACAGCATTTTTGG |  |
| pET28a-*rnj1*-F | GAGTCAggatccATGTCATCAGTCAATCTAAAACCTCATG | Fusion fragment for construction of pET28a-*RS00935* |
| pET28a-*rnj1*-R | CCCaagcttTTATCGGTCAGGCGTCAGAA |  |
| pET28a-*rnj2*-F | GAGTCAggatccATGAGTACAATTAAAATTATGGCTCTTGG | Fusion fragment for construction of pET28a-*RS07180* |
| pET28a-*rnj2*-R | CCCaagcttTTACTTGACTTCCATAATCACCGG |  |
| pET28a-F | TAATACGACTCACTATAGGG | Detection of the fragments inserted into pET28a |
| pET28a-R | TAATACGACTCACTATAGGG |  |
| pSET2-MS2-*rss03*-1F | GAGTCAgaattcAAGCAGGCAATATCTGTTATA | Fusion fragment A for construction of pSET2-MS2-*rss03* |
| pSET2-MS2-*rss03*-1R | AAACAGACCCTGATGGTGTCTGAAAAACGTACCCTGATGGTGTACGGATTCTATTCTACCATAATCTCAAGG |  |
| pSET2-MS2-*rss03*-2F | CATCAGGGTCTGTTTATGCGATGAGCCGAGTAG | Fusion fragment B for construction of pSET2-MS2-*rss03* |
| pSET2-MS2-*rss03*-2R | GAGTCAggatccAAAAAGCCCACCATAAAGGT |  |
| pSET2-MS2-negative-F | GAGTCAgaattcAAGCAGGCAATATCTGTTATATTATTGG | Fusion fragment for construction of pSET2-MS2-negative |
| pSET2-MS2-negative-R | GAGTCAggatccAAAAAGCCCACCATAAAGGTGGGCAAGGGCATCGTAAACAGACCCTGATGGTGTCTGAAA |  |
| Promotor-*gyrA*-F | CCGgaattcTAGTTTTATTTGGGGATGACTTCA | *gyrA* promotor (P*gyrA*) for constrction of P*gyrA*-*X*-*lacZ* |
| Promotor-*gyrA*-R | TGTGCTTCTATTATACCATATTGGC |  |
| *glpF*-*lacZ*-F | GCCAATATGGTATAATAGAAGCACAAGGTTGCTATACTGCCGCAGCT | Fusion fragment B for construction of P*gyrA*-*glpF-lacZ* |
| *glpF*-*lacZ*-R | CGCggatccCTATAACATATTTAGTAGGATGTTATACCCCTAAC |  |
| *glpF*-*lacZ*-F | GCCAATATGGTATAATAGAAGCACAAGGTTGCTATACTGCCGCAGCT | Fusion fragment B for construction of P*gyrA*-*glpF-*300 nt-*lacZ* |
| *glpF*-300 nt-*lacZ*-R | CGCggatccGTGACAGCTGGATTTAGGTGGGCA |  |
| *glpF*-*lacZ*-F | GCCAATATGGTATAATAGAAGCACAAGGTTGCTATACTGCCGCAGCT | Fusion fragment B for construction of P*gyrA*-*glpF-*400 nt-*lacZ* |
| *glpF*-400 nt-*lacZ*-R | CGCggatccATAGAGGACGATGGATGCCAA |  |
| *glpF*-*lacZ*-F | GCCAATATGGTATAATAGAAGCACAAGGTTGCTATACTGCCGCAGCT | Fusion fragment B for construction of P*gyrA*-*glpF-*345 nt-*lacZ* |
| *glpF*-345 nt-*lacZ*-R | CGCggatccGGGACTTGAGCCCATGGTAGG |  |
| *glpF*-*lacZ*-F | GCCAATATGGTATAATAGAAGCACAAGGTTGCTATACTGCCGCAGCT | Fusion fragment B for construction of P*gyrA*-*glpF-*353 nt-*lacZ* |
| *glpF*-353 nt-*lacZ*-R | CGCggatccGGAAGCCTGGGACTTGAGC |  |
| pTCV-F | GTTGAATAACACTTATTCCTATC | Detection of the fragments inserted into pTCV |
| pTCV-R | CTTCCACAGTAGTTCACCACC |  |
|  |  |  |
| **Gel retardation assays** | | |
| EMSA-rss03-F | TAATACGACTCACTATAGGGATGCGATGAGCCGAGTAGTGG | The fragment of rss03 sRNA with T7 promoter |
| EMSA-rss03-R | AAAAAGCCCACCATAAAGGTGGGCAAGG |  |
| EMSA-*glpR*-F | TAATACGACTCACTATAGGGGAGGTGATGAAATGGAACGAT | The fragment of *RS04010* mRNA with T7 promoter |
| EMSA-*glpR*-R | CTAAACTAGCCTTACTGTGAC |  |
| EMSA-*celR*-F | TAATACGACTCACTATAGGGTCACAGTAAGGCTAGTTTAGGTTGGG | The fragment of *RS04015* mRNA with T7 promoter |
| EMSA-*celR*-R | TTACAAATTTGTATGGACATGCACATC |  |
| EMSA-*glpR-celR*-F | TAATACGACTCACTATAGGGGAGGTGATGAAATGGAACGAT | The fragment of *RS04010-4015* operon [[3](#_ENREF_3)] with T7 promoter |
| EMSA-*glpR-celR*-R | TTACAAATTTGTATGGACATGCACATC |  |
| EMSA-*metE2*-F | TAATACGACTCACTATAGGGATCACTAAAACGAAAGAGAGACACACA | The fragment of *RS06365* mRNA with T7 promoter |
| EMSA-*metE2*-R | TTAAGACCAAAACTTCTCTGCAATTTC |  |
| EMSA-*pflA2*-F | TAATACGACTCACTATAGGGTTATAAAATGATGAGGATGAGATGATGAC | The fragment of *RS08065* mRNA with T7 promoter |
| EMSA-*pflA2*-R | TTATCCCTTAATTCTATTCAAGTACTCCT |  |
| EMSA-*araC*-F | TAATACGACTCACTATAGGGAAGCCTTTACACTTTTGGTATGG | The fragment of *RS00960* mRNA with T7 promoter |
| EMSA-*araC*-R | TTATTTGTGGATTTTTCGCCAGC |  |
| EMSA-*adhP*-F | TAATACGACTCACTATAGGGATATAAAAGAACTATATTAACACATCCAAAAATGT | The fragment of *RS01445* mRNA with T7 promoter |
| EMSA-*adhP*-R | TTAGTGTGAGTGGCAGAAGTC |  |
| EMSA-*satD*-F | TAATACGACTCACTATAGGGATAATAGAAATTAGAGGATAAGTTATGAATTATAT | The fragment of *RS04735* mRNA with T7 promoter |
| EMSA-*satD*-R | TTATCCTTCTTTCTGACTGAGCTG |  |
| EMSA-rss01-F | TAATACGACTCACTATAGGGACAGTGATAAATGTTTGTCAACACTTG | The fragment of rss01 sRNA with T7 promoter |
| EMSA-rss01-R | CTAGGATAGAAATGCCCCTCAAAATAG |  |
| EMSA-*glpF*-F | TAATACGACTCACTATAGGGAGGTTGCTATACTGCCGCAGC | The fragment of *RS09410* mRNA with T7 promoter |
| EMSA-*glpF*-R | CTATAACATATTTAGTAGGATGTTATAGAGGATAGCTCC |  |
| EMSA-*glpF*-300 nt-F | TAATACGACTCACTATAGGGAGGTTGCTATACTGCCGCAGC | The fragment of *glpF*-300 nt mRNA with T7 promoter |
| EMSA-*glpF*-300 nt-R | GTGACAGCTGGATTTAGGTGGGCAGG |  |
| EMSA-*glpF*-400 nt-F | TAATACGACTCACTATAGGGAGGTTGCTATACTGCCGCAGC | The fragment of *glpF*-400 nt mRNA with T7 promoter |
| EMSA-*glpF*-400 nt-R | ATAGAGGACGATGGATGCCAACATTG |  |
| EMSA-*glpF*-500 nt-F | TAATACGACTCACTATAGGGAGGTTGCTATACTGCCGCAGC | The fragment of *glpF*-500 nt mRNA with T7 promoter |
| EMSA-*glpF*-500 nt-R | GCAAGTTTGAAGCTGTGTGACGAAGA |  |
| EMSA-*glpF*-F | TAATACGACTCACTATAGGGAGGTTGCTATACTGCCGCAGC | Step 1 for the fragment of *glpF*-400 nt-mut mRNA with T7 promoter |
| EMSA-*glpF*-400 nt-mut-1R | AAGTACCTCCAGAGGGACTTGAGCCCATGGTAGG |  |
| EMSA-*glpF*-F | TAATACGACTCACTATAGGGAGGTTGCTATACTGCCGCAGC | Step 2 for the fragment of *glpF*-400 nt-mut mRNA with T7 promoter |
| EMSA-*glpF*-400 nt-mut-2R | CCAGCAAATTGGGCAAGTACCTCCAGAGGGACTTG |  |
| EMSA-*glpF*-F | TAATACGACTCACTATAGGGAGGTTGCTATACTGCCGCAGC | Step3 for the fragment of *glpF*-400 nt-mut mRNA with T7 promoter |
| EMSA-*glpF*-400 nt-mut-3R | ATGCCAACATTGCTCCAGCAAATTGGGCAAGTAC |  |
| EMSA-*glpF*-F | TAATACGACTCACTATAGGGAGGTTGCTATACTGCCGCAGC | Step 4 for the fragment of *glpF*-400 nt-mut mRNA with T7 promoter |
| EMSA-*glpF*-400 nt-mut-4R | ATAGAGGACGATGGATGCCAACATTGCTCCAGCAAA |  |
| EMSA-*glpF*-F | TAATACGACTCACTATAGGGAGGTTGCTATACTGCCGCAGC | The fragment of *glpF*-345 nt mRNA with T7 promoter |
| EMSA-*glpF*-345 nt-R | GGGACTTGAGCCCATGGTAGG |  |
| EMSA-rss03-F | TAATACGACTCACTATAGGGATGCGATGAGCCGAGTAGTGG | The fragment of rss03-204 nt mRNA with T7 promoter |
| EMSA-rss03-204 nt-R | AGACCTCCCTAGCGTCGAAGG |  |
| EMSA-rss03-F | TAATACGACTCACTATAGGGATGCGATGAGCCGAGTAGTGG | The fragment of rss03-191 nt mRNA with T7 promoter |
| EMSA-rss03-191 nt-R | GTCGAAGGGAGTGGGAAAGAA |  |
| EMSA-rss03-F | TAATACGACTCACTATAGGGAGGTTGCTATACTGCCGCAGC | Step 1 for the fragment of rss03-mut mRNA with T7 promoter |
| EMSA-rss03-mut-1R | TCCGAAGGCTAGCGTCGAAGGGAGTGG |  |
| EMSA-rss03-F | TAATACGACTCACTATAGGGAGGTTGCTATACTGCCGCAGC | Step 2 for the fragment of rss03-mut mRNA with T7 promoter |
| EMSA-rss03-mut-2R | CCTGCGTTTCAATTAAATGATCCGAAGGCTAGCGTCGAA |  |
| EMSA-rss03-F | TAATACGACTCACTATAGGGAGGTTGCTATACTGCCGCAGC | Step 3 for the fragment of rss03-mut mRNA with T7 promoter |
| EMSA-rss03-mut-3R | ACAACTATTCAAGGTCCGCTCCTGCGTTTCAATTAAATGATCCGAA |  |
| EMSA-rss03-F | TAATACGACTCACTATAGGGAGGTTGCTATACTGCCGCAGC | Step 4 for the fragment of rss03-mut mRNA with T7 promoter |
| EMSA-rss03-mut-4R | GATGAGAATAGAAGGTTCACACAACTATTCAAGGTCCGCTC |  |
| EMSA-rss03-F | TAATACGACTCACTATAGGGAGGTTGCTATACTGCCGCAGC | Step 5 for the fragment of rss03-mut mRNA with T7 promoter |
| EMSA-rss03-mut-5R | TGGGCAAGGGCATCGTTTCCGATGAGAATAGAAGGTTCACACAACT |  |
| EMSA-rss03-F | TAATACGACTCACTATAGGGAGGTTGCTATACTGCCGCAGC | Step 6 for the fragment of rss03-mut mRNA with T7 promoter |
| EMSA-rss03-mut-6R | AAAAAGCCCACCATAAAGGTGGGCAAGGGCATCGTTTC |  |
| EMSA-*tet(O)*-F | TAATACGACTCACTATAGGGAATAAATATGCGGCAAGGTATTCTTAAAT | The fragment of *RS03075* mRNA with T7 promoter |
| EMSA-*tet(O)*-R | AAAAAATCCATATGGCCTGGCGTATCTAT |  |
| EMSA-*sufD*-F | TAATACGACTCACTATAGGGTCGTGGTTTCCTAGGCTCTGT | The fragment of *RS09005* mRNA with T7 promoter |
| EMSA-*sufD*-R | CTAGATAGGGCCATTGAAAAACTCC |  |
| EMSA-*sufB*-F | TAATACGACTCACTATAGGGTAAAAGCTTTTGAAAAAAGATAAATCTCC | The fragment of *RS08995* mRNA with T7 promoter |
| EMSA-*sufB*-R | CTATCCCACTGAACCCTCC |  |
| EMSA-*RS01870*-F | TAATACGACTCACTATAGGGTGCACTAGAACCTTGCCC | The fragment of *RS01870* mRNA with T7 promoter |
| EMSA-*RS01870*-R | TCATAGCTGAACACCAGGC |  |
| EMSA-*RS10575*-F | TAATACGACTCACTATAGGGAAAAGGCTATAAAATATCTTGATGAACG | The fragment of *RS10575* mRNA with T7 promoter |
| EMSA-*RS10575*-R | CCCATTCTCTCTATATAACCAGG |  |
| EMSA-*mmuM*-F | TAATACGACTCACTATAGGGACATCCGCTTCAAGAAGTGGT | The fragment of *RS09725* mRNA with T7 promoter |
| EMSA-*mmuM*-R | TTATTTCAAACCTGCAACTAGGTCAG |  |
| EMSA-*tadA*-F | TAATACGACTCACTATAGGGGTAAAAATCAATATTGCTCCCCCTAC | The fragment of *RS09850* mRNA with T7 promoter |
| EMSA-*tadA*-R | CTAGCTATCCGAAGTTGCTTCCTCTT |  |
| EMSA-*RS01895*-F | TAATACGACTCACTATAGGGAAAAAATAGGAGAAATGCATGTCTATTTCAGGGA | The fragment of *RS01895* mRNA with T7 promoter |
| EMSA-*RS01895*-R | TCACTCCACCCTCACCGGCAC |  |
| EMSA-*RS07570*-F | TAATACGACTCACTATAGGGATTTCCTTCTTTGTATTGTGAGGATT | The fragment of *RS07570* mRNA with T7 promoter |
| EMSA-*RS07570*-R | TTAAACAAAAAATTCTACCCAAGCTC |  |
| EMSA-*RS09035*-F | TAATACGACTCACTATAGGGAATAGCATTTTAAAGGAGACACTATGAA | The fragment of *RS09035* mRNA with T7 promoter |
| EMSA-*RS09035*-R | TTAGTCATGTGGCACCTCGAC |  |
| EMSA-*acoA*-F | TAATACGACTCACTATAGGGAGAAAGAGAAAAACCTTTGGAGGAA | The fragment of *RS08855* mRNA with T7 promoter |
| EMSA-*acoA*-R | CTAGTCTACAAACACATCCTCATAAGCT |  |
| EMSA-*ychF*-F | TAATACGACTCACTATAGGGGATTGAATTTTTAATTGGAGAGTAAAAAAAACATGTCTTTA | The fragment of *RS00030* mRNA with T7 promoter |
| EMSA-*ychF*-R | TCAAACGTTAAAGCGGAACTCCATGA |  |
| EMSA-*RS06900*-F | TAATACGACTCACTATAGGGAGATTACAAGGAGGTTCATATGAGA | The fragment of *RS06900* mRNA with T7 promoter |
| EMSA-*RS06900*-R | TTAGACCCTATCGCCCTCCTTAACT |  |
| EMSA-*xerD*-F | TAATACGACTCACTATAGGGAAAATAGAGAATTTATGGGAATCATTACAC | The fragment of *RS08230* mRNA with T7 promoter |
| EMSA-*xerD*-R | TTATCTAAATTTTTCCAAACTCATGCT |  |
| EMSA-*RS05510*-F | TAATACGACTCACTATAGGGCTCACTCTATTGGGTAGTAAATTCAC | The fragment of *RS05510* mRNA with T7 promoter |
| EMSA-*RS05510*-R | TTACCGAATGCTCAATTCACC |  |
| EMSA-*fepD*-F | TAATACGACTCACTATAGGGGACTATAACCTCTTCTACTTCTCCGA | The fragment of *RS03755* mRNA with T7 promoter |
| EMSA-*fepD*-R | TCAAAAGTGTTTCTCCTTTCTAATCA |  |
| EMSA-rss26-F | TAATACGACTCACTATAGGGAAAGCTTACAGGTGCCTTGCTTTTTA | The fragment of rss26 sRNA with T7 promoter |
| EMSA-rss26-R | ACGCTCACTTTATCAATTTTAATGTT |  |
| EMSA-*xerC*-F | TAATACGACTCACTATAGGGCATAAAGAGAGCCAATTCTTCTAGTT | The fragment of *RS08625* mRNA with T7 promoter |
| EMSA-*xerC*-R | TTAACAAAGTCCGGAGAGTGA |  |
| EMSA-*rsuA*-F | TAATACGACTCACTATAGGGATGAGATTGGACAAATGTTTAGAGAAAG | The fragment of *RS03740* mRNA with T7 promoter |
| EMSA-*rsuA*-R | CTAGTCAAAATAAGTGAATAAATGTTCCATT |  |
| EMSA-*nanR*-F | TAATACGACTCACTATAGGGAGAAGCTATCAGAGGAATGGAAAATGTT | The fragment of *RS06815* mRNA with T7 promoter |
| EMSA-*nanR*-R | TTATTCTCTAATGGTTTGCTTGAAAATA |  |
| EMSA-*RS00345*-F | TAATACGACTCACTATAGGGGAAGCTTACGGGAGCCTGGCTTTTT | The fragment of *RS00345* mRNA with T7 promoter |
| EMSA-*RS00345*-R | TGGGTTGACTCATTTTCATGCGAAA |  |
| EMSA-*amiABC*-F | TAATACGACTCACTATAGGGGTACTAGGACTCATAACAATTTTTGC | The fragment of *RS06240-6235* operon with T7 promoter |
| EMSA-*amiABC*-R | TTAATTATCTAAAAGATAAAGCTTGT |  |
| EMSA-*usp*-F | TAATACGACTCACTATAGGGATGTAAGGAACTACTCTACCTATTTCGG | The fragment of *RS01765* mRNA with T7 promoter |
| EMSA-*usp*-R | TTATAAACTTTTTTCTCCATCGCGAACA |  |
| EMSA-*pezT*-F | TAATACGACTCACTATAGGGTTTACCTTGTCGCTACTTTCGA | The fragment of *RS03175* mRNA with T7 promoter |
| EMSA-*pezT*-R | TTATTTATTTTTCTCAAGTAATTCCTTAAGTAGG |  |
| EMSA-*RS00375*-F | TAATACGACTCACTATAGGGGAATCCTACATATCACTGAGTAACGC | The fragment of *RS00375* mRNA with T7 promoter |
| EMSA-*RS00375*-R | TTAGGATTCAGTTTGACAATTTTTCA |  |
| EMSA-*lacR*-F | TAATACGACTCACTATAGGGAAAAAAACAACAAAGGATTAAACATGAATA | The fragment of *RS05130* mRNA with T7 promoter |
| EMSA-*lacR*-R | TTATGATTTTGTAGAAATAATTTCAATGTG |  |
| EMSA-*fmt*-F | TAATACGACTCACTATAGGGATAGTATAATTGATATGAATCAGAAAATGA | The fragment of *RS02075* mRNA with T7 promoter |
| EMSA-*fmt*-R | TTATTGATCACCAAATTGATCTCCT |  |
| EMSA-*gpsA*-F | TAATACGACTCACTATAGGGAAATTACACCAACTAGAAAGGATTTTTTAT | The fragment of *RS09795* mRNA with T7 promoter |
| EMSA-*gpsA*-R | TCAATGCCATTCATTTTCATGTCGGAA |  |
| EMSA-*purM*-F | TAATACGACTCACTATAGGGAGCCTAGAAGAGAAAACGAGTTTCTAC | The fragment of *RS00240* mRNA with T7 promoter |
| EMSA-*purM*-R | TCATTTGATGACCACACTCTTGTCT |  |
| EMSA-*murR*-F | TAATACGACTCACTATAGGGATACAACTTTTGTATTATTATTTCAATATTTTT | The fragment of *RS09285* mRNA with T7 promoter |
| EMSA-*murR*-R | CTATTTGATTACTTCTGTAAGATGTT |  |
| EMSA-*ssnA*-F | TAATACGACTCACTATAGGGGTATATAACAACTGCAGACGTTTTTA | The fragment of *RS09450* mRNA with T7 promoter |
| EMSA-*ssnA*-R | CTGATGTTGACTTACTATTAGCAGAG |  |
| EMSA-*RS03305*-F | TAATACGACTCACTATAGGGTTTCTTGTAATAGAATAATGTGCTGA | The fragment of *RS03305* mRNA with T7 promoter |
| EMSA-*RS03305*-R | TTACTTAGTAGAAATTCCTCTTTTTG |  |
| EMSA-*araE*-F | TAATACGACTCACTATAGGGAGACTAAAACCAAAGGAGCCCTC | The fragment of *RS09150* mRNA with T7 promoter |
| EMSA-*araE*-R | TTAAATTGACGGTTCTTGGTCTTTATAGACT |  |
|  |  |  |
| **3' RACE** |  |  |
| GSP2-rss03 | GATTACGCCAAGCTTAGTGGTTGATTGGCAGATTTGTT | The fragment of first round of PCR for rss03-3' RACE |
| UPM-Long | CTAATACGACTCACTATAGGGCAAGCAGTGGTATCAACGCAGAGT |  |
| NGSP2-rss03 | ATTTTATACTCCAAATCTGACCTAATCA | The fragment of second round of PCR for rss03-3' RACE |
| UPM-Short | AAGCAGTGGTATCAACGCAGAGT |  |
| M13F | CGCCAGGGTTTTCCCAGTCACGAC | Detection of the fragments inserted into pMD 19-T |
| M13R | AGCGGATAACAATTTCACACAGGA |  |
|  |  |  |
| **RT-qPCR** | | |
| qRT-*rss03*-F | TCAGCGCAGTGGTTGATT | Detection of *rss03* sRNA transcriptional level |
| qRT-*rss03*-R | TCGTTTCCGATGAGAATAGAAGG |  |
| qRT-*glpF*-F | CCATCGTCCTCTATCTCCATTTC | Detection of *RS09410* mRNA transcriptional level |
| qRT-*glpF*-R | GCAACTGGGATCCAGCTATAA |  |
| qRT-16S rRNA-F | TGTCGTGAGATGTTGGGTTAAG | Detection of 16S rRNA transcriptional level |
| qRT-16S rRNA-R | CCACCTTCCTCCGGTTTATTAC |  |
| qRT-*parC*-F | AGGACTGGAAGAACCGCGAGATTT | Detection of *RS04270* mRNA transcriptional level |
| qRT-*parC*-R | TCCGTATCGTCAAAGTTCCAGGCA |  |

Underlined nucleotides denote reverse complement. Lowercase nucleotides denote restriction enzyme sites. Blue color nucleotides denote T7 promoter sequence.

# References

3. Liang Z, Lu J, Bao Y, et al. Glycerol metabolic repressor GlpR contributes to Streptococcus suis oxidative stress resistance and virulence. Microbes Infect. 2024 Feb 2:105307.
