# Supplementary material for: A small RNA from Streptococcus suis epidemic ST7 strain promotes bacterial survival in host blood and brain by enhancing oxidative stress resistance
Source: Virulence. 2025 Apr 16;16(1):2491635. doi: 10.1080/21505594.2025.2491635 (PMC12005413; doi:10.1080/21505594.2025.2491635)
Supplement: Table S9.docx [file KVIR_A_2491635_SM4065.docx]

# Table S9. Conservation analysis of *glpF* and its interaction region with rss03 in *S. suis* ST1 and ST7 strains.

|  |  |  |  | ***glpF*** | | **Interaction region with rss03 within *glpF*** | |
| --- | --- | --- | --- | --- | --- | --- | --- |
| **Number** | **Genbank accession** | **Strain** | **Sequence types** | **Coverage(%)** | **Homology(%)** | **Coverage(%)** | **Homology(%)** |
| 1 | NC_017618.1 | JS14 | ST7 | 100 | 100 | 100 | 100 |
| 2 | NZ_CP065431.1 | yp20190405 | ST1 | 100 | 100 | 100 | 100 |
| 3 | NZ_CP082198.1 | cNJ3 | ST1 | 100 | 100 | 100 | 100 |
| 4 | NZ_CP082199.1 | cFJSM5 | ST1 | 100 | 100 | 100 | 100 |
| 5 | NZ_CP082200.1 | cDY107 | ST1 | 100 | 100 | 100 | 100 |
| 6 | NZ_CP082201.1 | cAKJ18 | ST1 | 100 | 100 | 100 | 100 |
| 7 | NZ_CP102137.1 | M104300_S20 | ST1 | 100 | 100 | 100 | 100 |
| 8 | NZ_CP102154.1 | SS15055_N2_C15 | ST1 | 100 | 100 | 100 | 100 |
| 9 | NZ_CP095463.1 | TJS56 | ST1 | 100 | 100 | 100 | 100 |
| 10 | NZ_LS483418.1 | NCTC10234 | ST1 | 100 | 100 | 100 | 100 |
| 11 | NZ_CP082948.1 | SZ1908 | ST7 | 100 | 100 | 100 | 100 |
| 12 | NZ_CP030022.1 | ISU2714 | ST1 | 100 | 100 | 100 | 100 |
| 13 | NZ_CP030017.1 | ISU1606 | ST1 | 100 | 100 | 100 | 100 |
| 14 | NZ_CP024050.1 | CS100322 | ST7 | 100 | 100 | 100 | 100 |
| 15 | NC_012926.1 | BM407 | ST1 | 100 | 100 | 100 | 100 |
| 16 | NZ_LR738721.1 | S10 | ST1 | 100 | 100 | 100 | 100 |
| 17 | CP000408.1 | 98HAH33 | ST7 | 100 | 100 | 100 | 100 |
| 18 | NZ_CP058742.1 | 10 | ST1 | 100 | 100 | 100 | 100 |
| 19 | NC_018526.1 | S735 | ST1 | 100 | 100 | 100 | 100 |
| 20 | NC_012925.1 | P1/7 | ST1 | 100 | 100 | 100 | 100 |
| 21 | NC_017617.1 | GZ1 | ST1 | 100 | 100 | 100 | 100 |
| 22 | NC_017622.1 | A7 | ST7 | 100 | 100 | 100 | 100 |
| 23 | NZ_CP018908.1 | SS2-1 | ST7 | 100 | 100 | 100 | 100 |
| 24 | NZ_CP007497.1 | ZY05719 | ST7 | 100 | 100 | 100 | 100 |
| 25 | NC_012924.1 | SC84 | ST7 | 100 | 100 | 100 | 100 |
| 26 | NZ_CP020863.1 | SC19 | ST7 | 100 | 100 | 100 | 100 |
| 27 | NC_020526.1 | SC070731 | ST7 | 100 | 100 | 100 | 100 |
| 28 | NZ_CP085088.1 | Ssuis_MA1 | ST1 | 100 | 100 | 100 | 100 |
| 29 | NZ_CP139163.1 | cnzyss2-311 | ST1 | 100 | 100 | 100 | 100 |
| 30 | NZ_CP152119.1 | Ss2301 | ST7 | 100 | 99.86 | 100 | 100 |
| 31 | NZ_CP102748.1 | cSFJ45 | ST1 | 100 | 100 | 100 | 100 |
| 32 | NC_017619.1 | SS12 | ST1 | 100 | 100 | 100 | 100 |
| 33 | NZ_CP141904.1 | 8324 | ST1 | 100 | 100 | 100 | 100 |
| 34 | NZ_CP102746.1 | cAKJ47-2 | ST1 | 100 | 100 | 100 | 100 |
| 35 | NZ_CP139881.1 | Ss_21 | ST1 | 100 | 99.86 | 100 | 100 |
| 36 | NZ_CP139880.1 | Ss_22 | ST1 | 100 | 100 | 100 | 100 |
