# Supplementary material for: A small RNA from Streptococcus suis epidemic ST7 strain promotes bacterial survival in host blood and brain by enhancing oxidative stress resistance
Source: Virulence. 2025 Apr 16;16(1):2491635. doi: 10.1080/21505594.2025.2491635 (PMC12005413; doi:10.1080/21505594.2025.2491635)
Supplement: Table S5.docx [file KVIR_A_2491635_SM4064.docx]

# Table S5. The candidate targets obtained by CopraRNA.

| **Number** | **Gene_ID** | **Annotation** | ***p*-value*** |
| --- | --- | --- | --- |
| 1 | NJAUSS_RS00375 | hypothetical protein | 0 |
| 2 | NJAUSS_RS01870 | N-acetyltransferase | 0 |
| 3 | NJAUSS_RS10575 | histone acetyltransferase | 2.06E-05 |
| 4 | NJAUSS_RS09725 | homocysteine S-methyltransferase | 2.76E-05 |
| 5 | NJAUSS_RS09850 | tRNA adenosine(34) deaminase TadA | 6.29E-05 |
| 6 | NJAUSS_RS01895 | nucleotidyltransferase | 0.000658458 |
| 7 | NJAUSS_RS07570 | YSIRK-type signal peptide-containing protein | 0.001656018 |
| 8 | NJAUSS_RS09035 | glutamine ABC transporter substrate-binding protein | 0.001730383 |
| 9 | NJAUSS_RS08855 | pyruvate dehydrogenase E1 component subunit alpha | 0.001784009 |
| 10 | NJAUSS_RS00030 | redox-regulated ATPase YchF | 0.001839135 |
| 11 | NJAUSS_RS05130 | DeoR/GlpR transcriptional regulator | 0.002036901 |
| 12 | NJAUSS_RS09365 | aminopeptidase | 0.002274016 |
| 13 | NJAUSS_RS03825 | DNA replication initiation control protein YabA | 0.002537953 |
| 14 | NJAUSS_RS09665 | ribonuclease M5 | 0.002699624 |
| 15 | NJAUSS_RS02075 | methionyl-tRNA formyltransferase | 0.003020193 |
| 16 | NJAUSS_RS08475 | polyprenyl synthetase | 0.003030652 |
| 17 | NJAUSS_RS03150 | DNA primase | 0.003061456 |
| 18 | NJAUSS_RS02455 | amino acid ABC transporter ATP-binding protein | 0.003135785 |
| 19 | NJAUSS_RS09145 | DUF1761 domain-containing protein | 0.00314238 |
| 20 | NJAUSS_RS00985 | branched-chain amino acid ABC transporter permease | 0.003205033 |
| 21 | NJAUSS_RS09585 | ribosome small subunit-dependent GTPase A | 0.003343873 |
| 22 | NJAUSS_RS02065 | DNA-directed RNA polymerase subunit omega | 0.00355449 |
| 23 | NJAUSS_RS04040 | fructose-bisphosphate aldolase | 0.003622626 |
| 24 | NJAUSS_RS08050 | YSIRK-type signal peptide-containing protein | 0.003700718 |
| 25 | NJAUSS_RS06365 | 5-methyltetrahydropteroyltriglutamate-homocysteine methyltransferase | 0.00391677 |
| 26 | NJAUSS_RS03375 | lactate dehydrogenase | 0.004060842 |
| 27 | NJAUSS_RS04230 | ATP-dependent nuclease subunit B | 0.004369155 |
| 28 | NJAUSS_RS01685 | histidine triad protein | 0.004389145 |
| 29 | NJAUSS_RS09350 | 3-isopropylmalate dehydrogenase | 0.004524149 |
| 30 | NJAUSS_RS05195 | NAD kinase | 0.004567277 |
| 31 | NJAUSS_RS09795 | glycerol-3-phosphate dehydrogenase (NAD(P)(+)) | 0.004778246 |
| 32 | NJAUSS_RS06950 | DNA primase | 0.005068624 |
| 33 | NJAUSS_RS09930 | CHAP domain-containing protein | 0.005208955 |
| 34 | NJAUSS_RS04420 | KH domain-containing protein | 0.006018005 |
| 35 | NJAUSS_RS09420 | metalloprotease RseP | 0.006257929 |
| 36 | NJAUSS_RS06400 | UMP kinase | 0.006670681 |
| 37 | NJAUSS_RS04400 | SAM-dependent methyltransferase | 0.00677797 |
| 38 | NJAUSS_RS04515 | tRNA (guanosine(37)-N1)-methyltransferase TrmD | 0.006920307 |
| 39 | NJAUSS_RS00240 | phosphoribosylformylglycinamidine cyclo-ligase | 0.007185229 |
| 40 | NJAUSS_RS05370 | phosphate ABC transporter permease protein PstA | 0.00722714 |
| 41 | NJAUSS_RS05045 | amino acid ABC transporter ATP-binding protein | 0.007509237 |
| 42 | NJAUSS_RS08980 | ABC transporter ATP-binding protein | 0.007599895 |
| 43 | NJAUSS_RS02435 | NUDIX hydrolase | 0.007733513 |
| 44 | NJAUSS_RS07125 | hypothetical protein | 0.007837365 |
| 45 | NJAUSS_RS08590 | PTS mannose transporter subunit IID | 0.00788844 |
| 46 | NJAUSS_RS04380 | bifunctional pyrimidine operon transcriptional regulator/uracil phosphoribosyltransferase | 0.008129913 |
| 47 | NJAUSS_RS07650 | hypothetical protein | 0.00831544 |
| 48 | NJAUSS_RS05555 | dihydropteroate synthase | 0.0084395 |
| 49 | NJAUSS_RS04975 | LysR family transcriptional regulator | 0.008443801 |
| 50 | NJAUSS_RS10260 | ribonucleotide reductase assembly protein NrdI | 0.008456831 |
| 51 | NJAUSS_RS05640 | FadR family transcriptional regulator | 0.008535985 |
| 52 | NJAUSS_RS04525 | DeoR/GlpR transcriptional regulator | 0.008595612 |
| 53 | NJAUSS_RS02620 | hypothetical protein | 0.008700366 |
| 54 | NJAUSS_RS01230 | alpha alpha-phosphotrehalase | 0.009302562 |
| 55 | NJAUSS_RS09975 | leucine--tRNA ligase | 0.009411838 |
| 56 | NJAUSS_RS02625 | elongation factor Tu | 0.009780543 |
| 57 | NJAUSS_RS02600 | lipoprotein | 0.009808989 |
| 58 | NJAUSS_RS05145 | integrase | 0.010080034 |
| 59 | NJAUSS_RS06355 | DUF3042 domain-containing protein | 0.010121792 |
| 60 | NJAUSS_RS00045 | RNA-binding S4 domain-containing protein | 0.010191064 |
| 61 | NJAUSS_RS04805 | ABC transporter ATP-binding protein | 0.010222784 |
| 62 | NJAUSS_RS07575 | tRNA1(Val) (adenine(37)-N6)-methyltransferase | 0.010724754 |
| 63 | NJAUSS_RS06750 | peptidase C69 | 0.011049714 |
| 64 | NJAUSS_RS04940 | uracil-DNA glycosylase | 0.011079001 |
| 65 | NJAUSS_RS06980 | ADP-ribose pyrophosphatase | 0.011175404 |
| 66 | NJAUSS_RS00800 | type II secretion system protein | 0.011461525 |
| 67 | NJAUSS_RS06495 | peptide chain release factor 3 | 0.01191131 |
| 68 | NJAUSS_RS07990 | arginine repressor | 0.012086075 |
| 69 | NJAUSS_RS09165 | PucR family transcriptional regulator | 0.012194008 |
| 70 | NJAUSS_RS02310 | hypothetical protein | 0.012205416 |
| 71 | NJAUSS_RS06240 | cell wall hydrolase | 0.01230079 |
| 72 | NJAUSS_RS09010 | Fe-S cluster assembly protein SufD | 0.012790417 |
| 73 | NJAUSS_RS01415 | surface-anchored protein | 0.012816932 |
| 74 | NJAUSS_RS07075 | uracil-DNA glycosylase | 0.012844092 |
| 75 | NJAUSS_RS08015 | neutral zinc metallopeptidase | 0.013114206 |
| 76 | NJAUSS_RS05685 | ATP synthase subunit beta | 0.013410238 |
| 77 | NJAUSS_RS04010 | DeoR/GlpR transcriptional regulator | 0.01361084 |
| 78 | NJAUSS_RS09170 | DNA-binding response regulator | 0.014061038 |
| 79 | NJAUSS_RS02385 | cell division protein FtsA | 0.014080989 |
| 80 | NJAUSS_RS00340 | hypothetical protein | 0.014161947 |
| 81 | NJAUSS_RS09255 | serine O-acetyltransferase | 0.014229863 |
| 82 | NJAUSS_RS08535 | ABC transporter permease | 0.014967823 |
| 83 | NJAUSS_RS09320 | hypothetical protein | 0.015048837 |
| 84 | NJAUSS_RS06560 | DNA-binding response regulator | 0.015152287 |
| 85 | NJAUSS_RS08220 | segregation/condensation protein B | 0.015206592 |
| 86 | NJAUSS_RS05935 | CPBP family intramembrane metalloprotease | 0.015281141 |
| 87 | NJAUSS_RS08880 | translation initiation factor IF-2 | 0.015491724 |
| 88 | NJAUSS_RS02935 | 3-phosphoshikimate 1-carboxyvinyltransferase | 0.01560664 |
| 89 | NJAUSS_RS08800 | DUF1129 domain-containing protein | 0.016733515 |
| 90 | NJAUSS_RS01605 | membrane protein | 0.016831409 |
| 91 | NJAUSS_RS03900 | site-specific DNA-methyltransferase | 0.016932086 |
| 92 | NJAUSS_RS09360 | GlsB/YeaQ/YmgE family stress response membrane protein | 0.017887957 |
| 93 | NJAUSS_RS06795 | formamidopyrimidine-DNA glycosylase | 0.017979198 |
| 94 | NJAUSS_RS06600 | glycosyltransferase family 4 protein | 0.01802264 |
| 95 | NJAUSS_RS03820 | DNA polymerase III subunit delta' | 0.018196969 |
| 96 | NJAUSS_RS03680 | arginine regulator | 0.018619169 |
| 97 | NJAUSS_RS03050 | glucan-binding protein | 0.01867578 |
| 98 | NJAUSS_RS01565 | ABC transporter substrate-binding protein | 0.018702757 |
| 99 | NJAUSS_RS06810 | endoribonuclease YbeY | 0.018706128 |
| 100 | NJAUSS_RS01770 | pyridoxal phosphate-dependent aminotransferase | 0.018779061 |
| 101 | NJAUSS_RS03140 | hypothetical protein | 0.018933705 |
| 102 | NJAUSS_RS06985 | acyl-ACP thioesterase | 0.018947488 |
| 103 | NJAUSS_RS00925 | MerR family transcriptional regulator | 0.019010935 |
| 104 | NJAUSS_RS07115 | ABC transporter ATP-binding protein | 0.01904289 |
| 105 | NJAUSS_RS04935 | dihydroorotase | 0.019259095 |
| 106 | NJAUSS_RS07550 | pyridoxal phosphate-dependent aminotransferase | 0.019553195 |
| 107 | NJAUSS_RS06460 | sugar ABC transporter permease | 0.019640927 |
| 108 | NJAUSS_RS10915 | hypothetical protein | 0.019674149 |
| 109 | NJAUSS_RS06290 | DNA replication protein DnaD | 0.019727737 |
| 110 | NJAUSS_RS06310 | DUF3114 domain-containing protein | 0.019842051 |
| 111 | NJAUSS_RS01395 | aquaporin family protein | 0.020156999 |
| 112 | NJAUSS_RS08380 | thioredoxin-disulfide reductase | 0.020291729 |
| 113 | NJAUSS_RS07920 | lactoylglutathione lyase | 0.020427012 |
| 114 | NJAUSS_RS05390 | myo-inositol-1(or 4)-monophosphatase | 0.020438647 |
| 115 | NJAUSS_RS05580 | hypothetical protein | 0.020658144 |
| 116 | NJAUSS_RS01440 | histidine--tRNA ligase | 0.02080829 |
| 117 | NJAUSS_RS08920 | ABC transporter ATP-binding protein | 0.02091969 |
| 118 | NJAUSS_RS01070 | YbaB/EbfC family nucleoid-associated protein | 0.020997859 |
| 119 | NJAUSS_RS08260 | diaminopimelate decarboxylase | 0.021027812 |
| 120 | NJAUSS_RS05670 | UDP-N-acetylglucosamine 1-carboxyvinyltransferase | 0.021130089 |
| 121 | NJAUSS_RS04015 | DNA-binding transcriptional regulator | 0.021163357 |
| 122 | NJAUSS_RS07750 | MarR family transcriptional regulator | 0.021362857 |
| 123 | NJAUSS_RS05575 | VTC domain-containing protein | 0.021370956 |
| 124 | NJAUSS_RS03165 | hypothetical protein | 0.021395779 |
| 125 | NJAUSS_RS01975 | DNA-directed RNA polymerase subunit delta | 0.021432712 |
| 126 | NJAUSS_RS00770 | DNA-directed RNA polymerase subunit beta' | 0.021546765 |
| 127 | NJAUSS_RS09735 | glutamate--tRNA ligase | 0.021826635 |
| 128 | NJAUSS_RS01970 | YebC/PmpR family DNA-binding transcriptional regulator | 0.022254819 |
| 129 | NJAUSS_RS05340 | DNA-binding response regulator | 0.022364002 |
| 130 | NJAUSS_RS06145 | membrane protein | 0.022712611 |
| 131 | NJAUSS_RS02275 | helicase | 0.022800166 |
| 132 | NJAUSS_RS03760 | ferrichrome ABC transporter substrate-binding protein | 0.023030806 |
| 133 | NJAUSS_RS04110 | NADH oxidase | 0.023132614 |
| 134 | NJAUSS_RS10150 | GNAT family acetyltransferase | 0.023533736 |
| 135 | NJAUSS_RS02490 | MerR family transcriptional regulator | 0.024162843 |
| 136 | NJAUSS_RS04770 | DNA-binding response regulator | 0.024857374 |
| 137 | NJAUSS_RS02405 | YggT family protein | 0.025095942 |
| 138 | NJAUSS_RS06375 | YozE family protein | 0.025244461 |
| 139 | NJAUSS_RS04960 | orotidine-5'-phosphate decarboxylase | 0.026038859 |
| 140 | NJAUSS_RS02740 | bifunctional DnaQ family exonuclease/ATP-dependent helicase | 0.026046959 |
| 141 | NJAUSS_RS08620 | type I restriction endonuclease subunit S | 0.026192099 |
| 142 | NJAUSS_RS05755 | signal recognition particle-docking protein FtsY | 0.026587787 |
| 143 | NJAUSS_RS00725 | metal ABC transporter permease | 0.027068808 |
| 144 | NJAUSS_RS03620 | RidA family protein | 0.027392635 |
| 145 | NJAUSS_RS04500 | guanosine monophosphate reductase | 0.027717854 |
| 146 | NJAUSS_RS06190 | glycosyltransferase family 8 protein | 0.027920815 |
| 147 | NJAUSS_RS08425 | transcriptional regulator | 0.02855037 |
| 148 | NJAUSS_RS06370 | PhoH family protein | 0.028683465 |
| 149 | NJAUSS_RS06605 | glycosyl transferase | 0.028947166 |
| 150 | NJAUSS_RS03210 | DNA-binding response regulator | 0.029482462 |
| 151 | NJAUSS_RS01630 | NADP-dependent oxidoreductase | 0.029730837 |
| 152 | NJAUSS_RS08715 | HAD family phosphatase | 0.029800141 |
| 153 | NJAUSS_RS00860 | DUF4479 domain-containing protein | 0.030244439 |
| 154 | NJAUSS_RS03735 | glutathione S-transferase family protein | 0.030874393 |
| 155 | NJAUSS_RS01265 | thioredoxin | 0.031353395 |
| 156 | NJAUSS_RS05645 | beta-glucuronidase | 0.031626861 |
| 157 | NJAUSS_RS05605 | glycosyl hydrolase | 0.031794365 |
| 158 | NJAUSS_RS08950 | dihydroxyacetone kinase | 0.032015769 |
| 159 | NJAUSS_RS06815 | RpiR family transcriptional regulator | 0.032304571 |
| 160 | NJAUSS_RS03385 | DNA (cytosine-5-)-methyltransferase | 0.032511643 |
| 161 | NJAUSS_RS00265 | 5-(carboxyamino)imidazole ribonucleotide synthase | 0.032639221 |
| 162 | NJAUSS_RS06300 | adenine phosphoribosyltransferase | 0.032983874 |
| 163 | NJAUSS_RS02110 | Cof-type HAD-IIB family hydrolase | 0.033138724 |
| 164 | NJAUSS_RS03275 | Msr family ABC-F type ribosomal protection protein | 0.033321699 |
| 165 | NJAUSS_RS01815 | LacI family transcriptional regulator | 0.03343526 |
| 166 | NJAUSS_RS04590 | dihydrofolate reductase | 0.03343766 |
| 167 | NJAUSS_RS00835 | bifunctional folylpolyglutamate synthase/dihydrofolate synthase | 0.033819445 |
| 168 | NJAUSS_RS06285 | SAM-dependent methyltransferase | 0.034062858 |
| 169 | NJAUSS_RS09830 | hypothetical protein | 0.034348794 |
| 170 | NJAUSS_RS10415 | energy-coupling factor transporter ATPase | 0.034554643 |
| 171 | NJAUSS_RS08605 | type I restriction-modification system subunit M | 0.034915498 |
| 172 | NJAUSS_RS05360 | phosphate ABC transporter ATP-binding protein | 0.034930676 |
| 173 | NJAUSS_RS04055 | aspartate-semialdehyde dehydrogenase | 0.035124917 |
| 174 | NJAUSS_RS04125 | bifunctional hydroxymethylpyrimidine kinase/phosphomethylpyrimidine kinase | 0.035275473 |
| 175 | NJAUSS_RS04190 | VOC family protein | 0.035403896 |
| 176 | NJAUSS_RS07295 | phosphoserine phosphatase SerB | 0.035729311 |
| 177 | NJAUSS_RS01520 | ACT domain-containing protein | 0.035960459 |
| 178 | NJAUSS_RS00965 | sugar ABC transporter substrate-binding protein | 0.035970978 |
| 179 | NJAUSS_RS05595 | N-acetyltransferase | 0.036152945 |
| 180 | NJAUSS_RS06610 | cell surface protein | 0.036347554 |
| 181 | NJAUSS_RS04620 | elongation factor 4 | 0.036397016 |
| 182 | NJAUSS_RS09950 | PTS sugar transporter subunit IIB | 0.036565227 |
| 183 | NJAUSS_RS08875 | ribosome-binding factor A | 0.036698193 |
| 184 | NJAUSS_RS00950 | ribosomal-protein-alanine N-acetyltransferase | 0.036718367 |
| 185 | NJAUSS_RS05395 | UPF0223 family protein | 0.036951134 |
| 186 | NJAUSS_RS00475 | 50S ribosomal protein L29 | 0.037075734 |
| 187 | NJAUSS_RS05680 | ATP synthase epsilon chain | 0.037078265 |
| 188 | NJAUSS_RS09575 | thiamine diphosphokinase | 0.037355869 |
| 189 | NJAUSS_RS04145 | uridine phosphorylase | 0.037451311 |
| 190 | NJAUSS_RS09445 | bifunctional glutamate-cysteine ligase GshA/glutathione synthetase GshB | 0.037513647 |
| 191 | NJAUSS_RS10225 | 4-alpha-glucanotransferase | 0.038135988 |
| 192 | NJAUSS_RS00205 | aminotransferase A | 0.0383918 |
| 193 | NJAUSS_RS08065 | pyruvate formate lyase-activating protein | 0.038522221 |
| 194 | NJAUSS_RS08030 | DNA-binding response regulator | 0.038686087 |
| 195 | NJAUSS_RS07230 | acetolactate decarboxylase | 0.038779853 |
| 196 | NJAUSS_RS06550 | MBL fold metallo-hydrolase | 0.038960611 |
| 197 | NJAUSS_RS03025 | CHAP domain-containing protein | 0.038968962 |
| 198 | NJAUSS_RS00485 | 50S ribosomal protein L14 | 0.039098622 |
| 199 | NJAUSS_RS03845 | 3-phosphoglycerate dehydrogenase | 0.039155944 |
| 200 | NJAUSS_RS09815 | acetyldiaminopimelate deacetylase | 0.039320276 |

* The order of the candidate targets is based on *p*-value. The red color indicates the direct targets identified by gel retardation assays. The green color indicates the indirect targets identified by gel retardation assays.
