# Supplementary material for: A small RNA from Streptococcus suis epidemic ST7 strain promotes bacterial survival in host blood and brain by enhancing oxidative stress resistance
Source: Virulence. 2025 Apr 16;16(1):2491635. doi: 10.1080/21505594.2025.2491635 (PMC12005413; doi:10.1080/21505594.2025.2491635)
Supplement: Table S3.docx [file KVIR_A_2491635_SM4062.docx]

# Table S3. Information on sRNA rss03 used in CopraRNA.

| **Number** | **NCBI reference genome number** | **Conserved sequence** |
| --- | --- | --- |
| 1 | >NC_020526 | CACTTCTAAGAGTTCGTGTCAACATCTCAGCGCAGTGGTTGATTGGCAGATTTGTTCGTATTTTATACTCCAAATCTGACCTAATCAACTGTGCGGGGGTGGGAAGACGAACTCTTGGTTGACAAGTTGAGTTCTTTCCCACTCCCT |
| 2 | >NC_012925 | CACTTCTAAGAGTTCGTGTCAACATCTCAGCGCAGTGGTTGATTGGCAGATTTGTTCGTATTTTATACTCCAAATCTGACCTAATCAACTGTGCGGGGGTGGGAAGACGAACTCTTGGTTGACAAGTTGAGTTCTTTCCCACTCCCT |
| 3 | >NZ_CP039462 | CACTTCTAAGAGTTCGTGTCAACATCTCAGCGCAGTGGTTGATTGGCAGATTTGTTCGTATTTTATACTCCAAATCTGACCTAATCAACTGTGCGGGGGTGGGAAGACGAACTCTTGGTTAACAAGTTGAGTTCTTTCCCACTCCCT |
| 4 | >NZ_CP030124 | ACACTTCTAAGAGTTCGCGTCAACATCTCAGCGCAGTGGTTGATTGGCAGATTTGTTCGTATTTTATACTCCAAATCTGACCTAATTAACTGTGCGGGGGTGGGAAGACGAACTCTTGGTTGACAAGTTGAGTTCTTTCCCACTCCCT |
| 5 | >NZ_CP017142 | CACTACTCAGAGTTCGTGTCAACATCTCAGCGCAGTGGTTGATTGGCAGATTTGTTCGTGTTTTGCACTCCAAATCTGACCTAATCAACTGTGCGGGGGTGGGAAGACGAACTCTTTTTGACTTAGTCGAGTTCTTTCCCACTCCCCTC |
| 6 | >NZ_LT906439 | CACACCTCTCATAGTTTGCGTCAACATCTCAGCGCAGTGGTTGATTGGCGGATTTGTTCGTGTTTTACACTCCAAATCCGACCTAATCAACTGTGCGGGGGTGGGAAAACGAACTCTTTTTTTGATTGCTGAGTTCTTTCCCACTCCCT |
| 7 | >NZ_CP025536 | AGAGTTTGTTCTAAGATCCTAACGCAGTGGTTGAATGGCTGTTCCGCTCGCATTCTATGCTTCGAACCAGACTTATTCAACTGTGCGGGGGTGGGAAGACGAACTCTGTTTTATTATCGGGAGTTCTTTCCCACTCCCTT |
| 8 | >NZ_CP043405 | AGAGTTCGTTTTAACATCATAACGCAGTGGTTGAATGGCAGTTTCGTTCGCGTTTTACGCTCTGAACCTGACCTATTCAACTGTGCGGGGGTGGGAAGACGAACCCTTTTGTTATTGTTGGGTTCTTTCCCACTCCCTT |
