# Supplementary material for: A small RNA from Streptococcus suis epidemic ST7 strain promotes bacterial survival in host blood and brain by enhancing oxidative stress resistance
Source: Virulence. 2025 Apr 16;16(1):2491635. doi: 10.1080/21505594.2025.2491635 (PMC12005413; doi:10.1080/21505594.2025.2491635)
Supplement: Supplementary Figure Legends.docx [file KVIR_A_2491635_SM4060.docx]

**Supplementary Figure legends**

**Figure S1. Flowchart of animal infection assays.**

**Figure S2. Information on the length and structure of rss03.** (a) 3' RACE results of rss03. Lane M: DNA marker. Lanes 1: The 3' RACE product with an expected length of 250 bp. (b) The secondary structure of rss03 homologs is predicted in representative *S. suis* strain by RNAfold. Numbers indicate nucleotide positions relative to the 5' end of the transcript. For these sRNAs, the regions conserved within *S. suis* have been shown by different colors: red indicating nucleotide Identity 100%, blue indicating nucleotide Identity ≥50%, and black indicating nucleotide Identity <50%.

**Figure S3. rss03 is conserved in *S. parasuis* and *S. ruminantium*.** (a) Map of the location of rss03 homologs in genomes of representative *S. parasuis* and *S. ruminantium* strains. Homologous genes are depicted using the same colors. Red arrows represent rss03 and its homologs. (b) Alignment of rss03 homologous sequences, including the promoter regions from representative *S. parasuis* and *S. ruminantium* strains. (c) Prediction of the secondary structure of rss03 homologs in representative *S. parasuis* and *S. ruminantium* strains. Red indicates nucleotide Identity 100%, blue indicates nucleotide Identity ≥50%, and black indicates nucleotide Identity <50%.

**Figure S4. Source images of gel retardation assays reported in Figure 2b and 2c.** The red dashed box outlines the source images corresponding to the each gel retardation assay results in Figure 2b (a) and Figure 2c (b). Red annotations depict the radiolabeled sRNA rss03 (rss03*) and its candidate target RNAs under validation.

**Figure S5. rss03 can bind multiple targets.** Gel retardation assays using rss03 and 15 candidate targets from the top 40 identified by MAPS (a), proteome (b), and CopraRNA (c), respectively. The genes marked red in the box indicate the direct targets based on gel retardation assays. Genes marked with underline mean the results of gel retardation assays were shown in Figure 2b and 2c. For gel source data, see Figure S6.

**Figure S6. Source images of gel retardation assays reported in Figure S5.** The red or dark dashed box outlines the source images corresponding to the each gel retardation assay results in Figure S5a (a), Figure S5b (b), and Figure S5c (c). Red or dark annotations depict the radiolabeled sRNA rss03 (rss03*) and its candidate target RNA under validation.

**Figure S7. GlpF belongs to aquaglyceroporins.** Evolutionary tree and amino acid sequences comparison of GlpF homologs from various species. Protein secondary structures were predicted by PSIPRED. Protein transmembrane regions were predicted by Deep TMHMM. Asterisks indicate substrate-selective residues in different aquaporin subfamilies.

**Figure S8. Source images of gel retardation assays reported in Figure 3.** The red or dark dashed box outlines the source images corresponding to the each gel retardation assay results in Figure 3c (a), Figure 3d (b), Figure 3h (c) and Figure 3j (d). Red or dark annotations depict the radiolabeled sRNA rss03 (rss03*) and different glpF mRNA variants (a, b), as well as the radiolabeled glpF-400 nt (glpF-400 nt*) and different sRNA rss03 variants (c, d) under validation.

**Figure S9. Source images of RNases *in vitro* degradation assay reported in Figure 4.** The red or dark dashed box outlines the source images corresponding to the each RNases in vitro degradation assay results in Figure 4g. Red or dark annotations indicate the radiolabeled glpF-400 nt (glpF-400 nt*), sRNA rss03 and the corresponding RNase under validation.

**Figure S10. rss03 had no effect on the bacterial load of *S. suis* in the kidney, spleen, and liver of mice.** Bacterial load of WT, Δ*rss03*, and CΔ*rss03* in the kidney, spleen, and liver after 12 h of intraperitoneal injection in mice. Data represent the mean ± SEM (n = 5, ns indicates insignificance, two-tailed unpaired t test).

**Figure S11. The predicated secondary structure of *glpF*-400 nt transcript.** The secondary structure of *glpF*-400 nt is predicted in *S. suis* SC070731 strain by RNAfold. Numbers indicate nucleotide positions relative to the 5' end of the transcript. The red color indicates stem-loop structure within *glpF*-400 nt transcript. The initiation codon is marked with a blue circle. The Shine-Dalgarno (SD) sequence is marked with a green circle. rss03 binding region is marked with an orange circle.

**Figure S12. Schematic of predicted binding regions between rss03 and its direct targets.** Numbers denote nucleotide positions relative to the start codon of target mRNA or the TSS of rss03. Nucleotides marked in green indicate the potential seed region of rss03.
